# Moderated Poster Abstracts for the 17th Asia Pacific Heart Rhythm Society (APHRS) Scientific Sessions

**DOI:** 10.1002/joa3.70001

**Published:** 2025-03-14

## 1 PULSED FIELD ABLATION FOR AF IN PATIENTS WITH CARDIAC IMPLANTABLE ELECTRONIC DEVICES

### 1.1 **MOHAMED ABBAS** ^1^, MEHRDAD EMAMI^1^, SURAYA KAMASANI^1^, RAJEEV PATHAK^2^, PRASH SANDERS^3^

#### 1.1.1 ^1^Royal Adelaide Hospital, Adelaide, Australia,^2^Canberra Heart Rhythm centre, Canberra, Australia,^3^Royal Adelaide Hospital, Do not wish to disclose, Australia

**Introduction:** Pulsed‐field ablation (PFA) is an innovative energy source used for pulmonary vein isolation (PVI) in patients with atrial fibrillation (AF). While multiple studies have demonstrated the safety of first‐generation PFA systems; however, patients with cardiac implantable electronic devices (CIEDs) have been excluded due to concerns about the impact of strong electrical fields on device function and integrity. This study assesses the function and integrity of CIEDs before and after PFA using the CathRx adjustable loop mapping and ablation catheter.

**Methods:** Patients with paroxysmal or persistent AF undergoing ablation were studied at two sites. The CathRx system utilizes an 8F variable loop catheter with 10 electrodes, delivering a train of seven biphasic and bipolar pulses with a peak‐to‐peak field strength of 5600 volts. Ablation was performed to achieve pulmonary vein (PV) and posterior left atrial isolation (PWI). CIEDs were examined before and after PFA to assess function (threshold, sensing), integrity (impedance), and arrhythmia episodes.

**Results:** We performed PFA on 9 patients with CIEDs using the CathRx system (mean age 73.2 ± 4.5 years, 55.5% male) for PVI and PWI. One patient had a cardiac resynchronization device, while eight had dual‐chamber pacemakers. In all cases, the right ventricular (RV) lead position was mid‐RV septum, and the right atrial (RA) lead was in the RA appendage. Each patient received an average of 60 ± 6.8 PFA applications, achieving complete PVI and PWI in all cases. Post‐procedural testing showed no changes in impedance, pacing threshold, or sensing of intrinsic activity. No lead damage or dislocation occurred, and no device malfunctions were observed during the procedures. Additionally, there were no major periprocedural complications.

**Conclusions:** The function and integrity of pacemakers were not compromised by PFA in our patient sample with the CathRx system. Further research with larger patient cohorts is necessary to confirm these findings.

## 2 P‐WAVE‐PR SEGMENT DURATION RATIO AND OCCURRENCE OF ATRIAL FIBRILLATION

### 2.1 **RISHI ADA** ^1^, SAMUEL GEORGE^1^, CLAUDIA LUCAS^2^, USMAN HUSSAIN^1^, MARIA KLESEWETTER^1^, ANDRE TAYLOR^1^, MOEEN ABEDIN^1,2^, ZAINUL ABEDIN^1,2^

#### 2.1.1 ^1^Paul L. Foster School of Medicine, El Paso, TX,^2^University Medical Center of El Paso, El Paso, TX

**Introduction:** P‐wave duration (PWD) is a reflection of atrial conduction time. Slowing of the atria conduction whether due to scarring, fibrosis or chamber enlargement could contribute to the occurrence of atrial fibrillation. The ratio of PWD and PR segment duration (PRSD) and its relationship to the occurrence of atrial fibrillation has not been previously reported.

**Methods:** This was a retrospective study. 742 consecutive electrocardiograms, recorded using MUSE ECG diagnostic software (GE healthcare system, California USA) were analyzed. Patients who had both sinus rhythm and atrial fibrillation ECGs available for analysis were included. PWD, from the onset of the P‐wave to its termination and PRSD from the onset of the P‐wave to the onset of the QRS complex was measured in milliseconds in Lead II of the 12 lead surface electrocardiogram. P‐PR ratio was calculated. Patients whose sinus rhythm ECG showed P‐wave duration of more than 60% of total PR segment were further analyzed for occurrence of atrial fibrillation. Statistical analysis was performed.

**Results:** Out of 742 ECGs reviewed, 567 patients had ECG available both in sinus rhythm and atrial fibrillation (flowchart1, Table 1) Sensitivity was 32.2%, specificity was 83.2%, positive predictive value was 80.6%. Using the chi‐square statistics the P value was ≤ 0.00001.

**Conclusions:** A PWD‐PRSD ratio that exceeds 60% was found to have high degree of specificity and positive predictive value and low sensitivity for occurrence of atrial fibrillation.

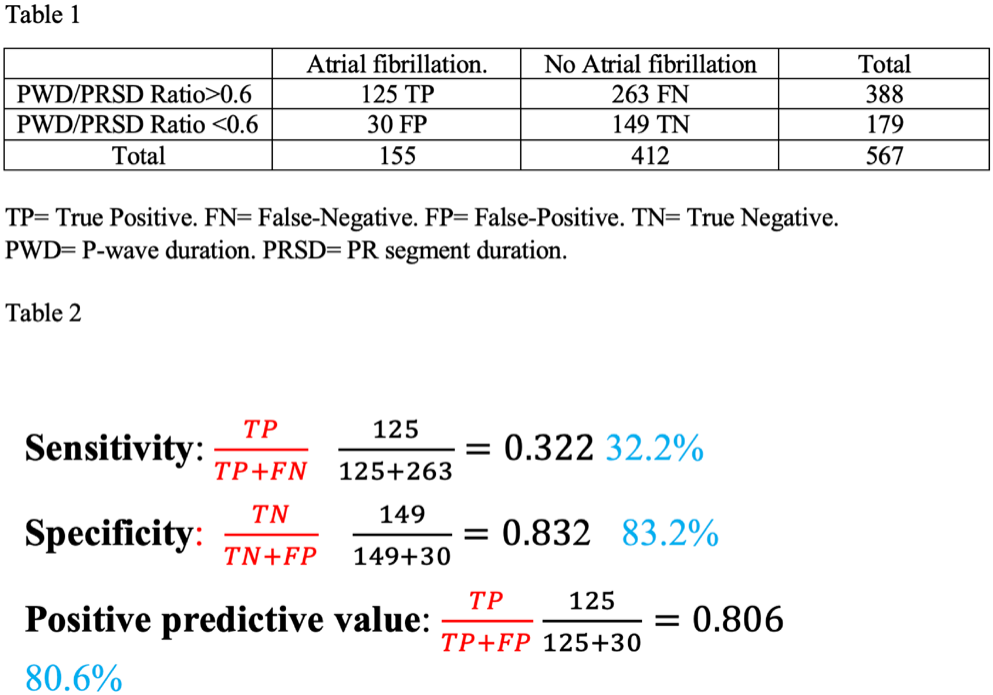

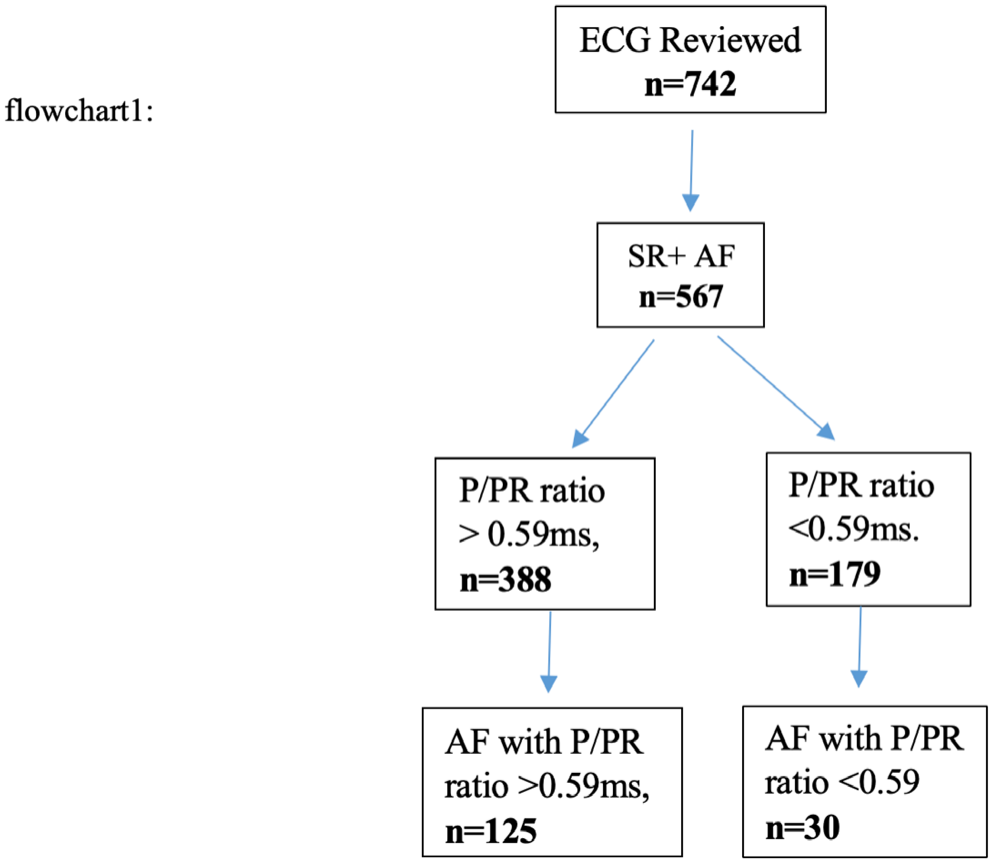

## 3 HYPERTROPHIC CARDIOMYOPATHY IN OMAN

### 3.1 **MOHAMED AL RAWAHI** ^1^, TASNEEM AL RASHDI^2^, NUHA AL HABSI^3^, MUHAMMAD SADIQ^1^, NAJIB AL RAWAHI^2^, ISMAIL AL ABRI^2^, ADIL AL RIYAMI^1^

#### 3.1.1 ^1^Sultan Qaboos University Hospital, Muscat, Oman,^2^National Heart Center, Royal Hospital, Muscat, Oman,^3^Oman Medical Speciality Board, Muscat, Oman

**Introduction:** Hypertrophic cardiomyopathy (HCM) is a common yet often under‐diagnosed monogenic cardiac disorder characterized by diverse phenotypic presentations. There is a lack of studies on HCM in Oman, a country with a population of 4.6 million. The worldwide prevalence of HCM is estimated to be around 1:200, with only 10% of cases clinically diagnosed. This study aims to evaluate the prevalence, clinical presentation, imaging features and predictors of arrhythmias in patients with HCM in Oman.

**Methods:** This retrospective cohort study included all patients aged 16 years and above diagnosed with HCM at Oman's two largest cardiology between January 2016 and December 2023.

**Results:** A total of 110 patients with HCM were enrolled during the study period, with a mean age at diagnosis of 35 years and a male predominance (78%). Most patients (75%) were sporadic cases with no family history of HCM. The most common clinical presentations included dyspnea (31%), chest pain (24%), palpitations (33%), and syncope (12%). AF was present in 16% of patients, all of whom were anticoagulated regardless of age. The mean left ventricular thickness on echocardiography was 22 ± 6 mm, with the septal variant being the most common (68%). Septal reduction therapy was performed in 7 patients due to symptomatic left ventricular outflow tract obstruction (5 patients underwent myomectomy, and 2 patients underwent alcohol septal ablation). According to the 2020 AHA/ACC HCM guidelines, 62 patients had an indication for ICD therapy, but only 27 patients had an ICD implanted. Compared to 10 patients eligible for defibrillator therapy with the ESC HCM risk calculator of more than 6%, only 7 patients had an ICD implanted. Seven patients had appropriate therapies for ventricular tachycardia. Six patients died from arrhythmic etiology, all without an ICD and before undergoing cardiac MRI.

**Conclusions:** This study represents the first comprehensive analysis of HCM in Oman and highlights its severe underdiagnosis in the country. The relatively young age at diagnosis and the high prevalence of risk factors for ventricular arrhythmias underscore the need for greater awareness and diagnostic vigilance. The study led to a genetic cardiomyopathy clinic in Oman.

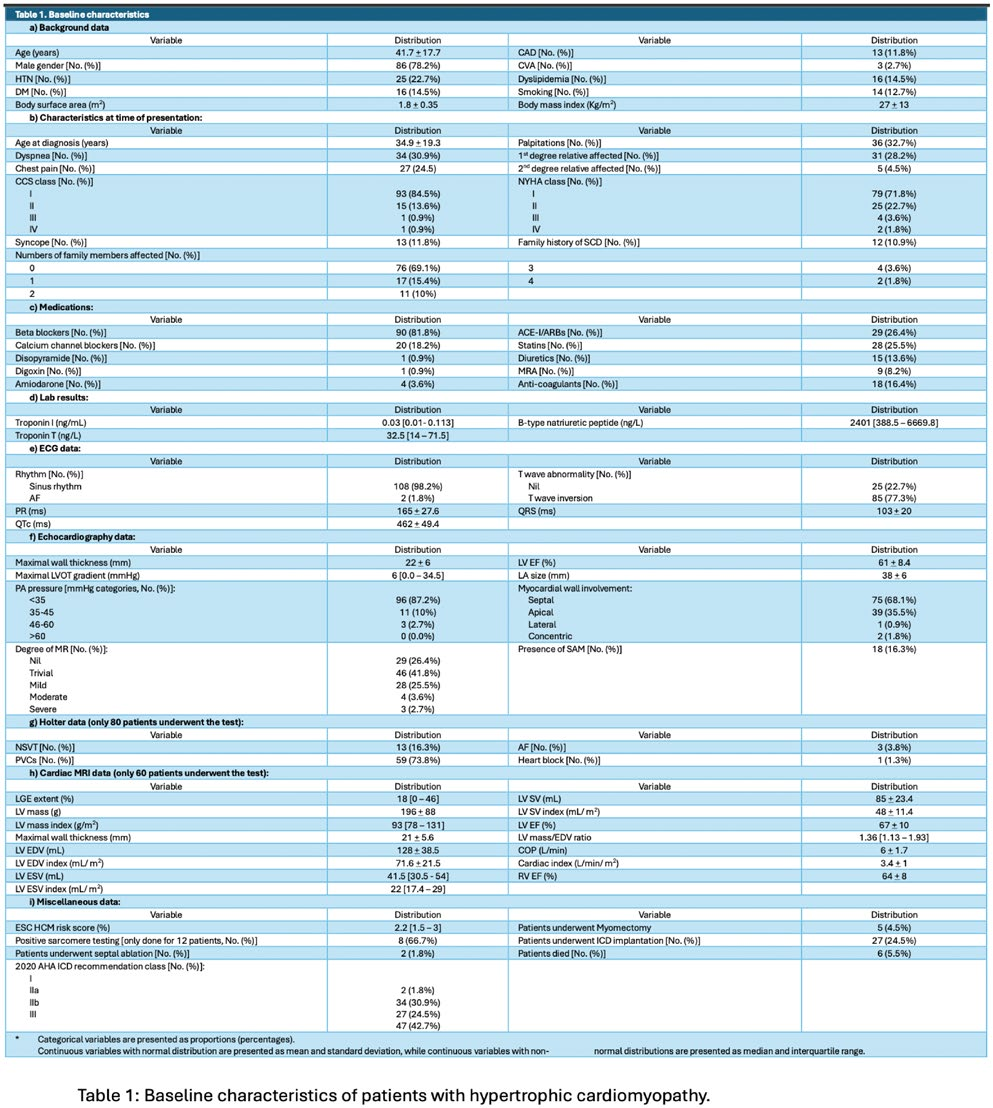

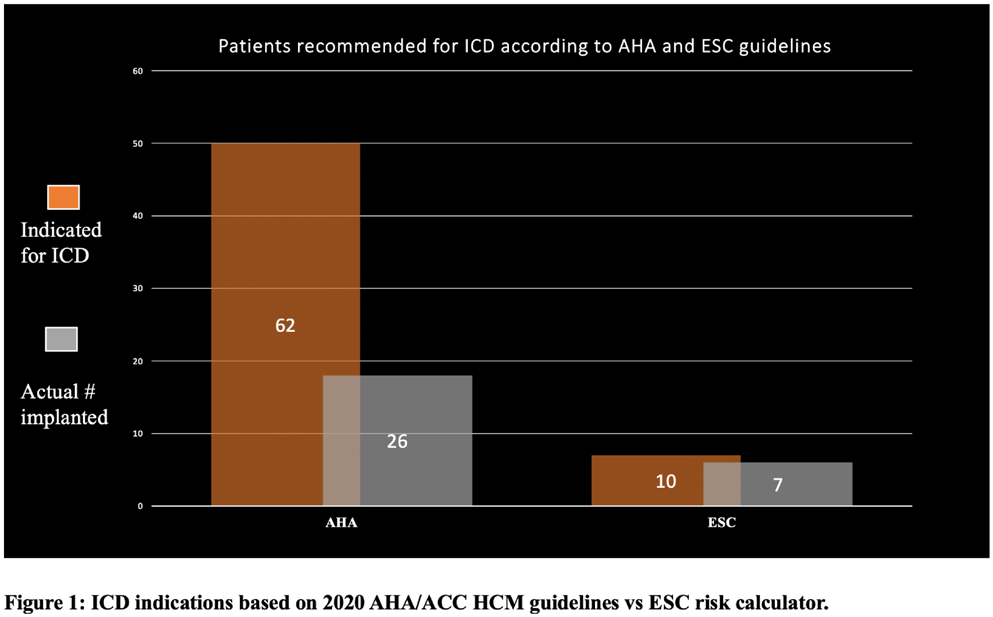

Chair

**A. Al Sinan**;

., New Zealand

## 4 VALUE OF DIAGNOSIS OF LEFT SEPTAL FASCICULAR BLOCK TO PREDICT NLBBB AND AVB POST‐TAVR

### 4.1 **SAMAH ALKHARJI**, KRISHNA KUMAR MOHANAN NAIR, AHMED ALSHATTI

#### 4.1.1 Dabbous Cardiac Center, Kuwait city, Kuwait

**Introduction:** TAVR is associated with a risk of high‐grade AV block and permanent pacemaker implantation (PPI)^2^. First degree AVB, LAFB or RBBB are known predictors of high‐grade AVB (HGAVB) and PPI ^3^. Loss of septal Q waves (SQ) is an infrequently reported conduction abnormality. Activation of LSF produces Q waves on in leads I, V5, V6. Loss of SQ is one of the proposed ECG criteria of LSFB ^7^. This study highlights the value of a subtle pre‐TAVR ECG abnormality in the development of new BBB (nBBB) or HGAVB requiring PPI in a single center experience.

**Methods:** We reviewed medical records of TAVR procedures performed in our center from 2014 to 2024. Kuwait ministry of health research committee approval was obtained prior to data collection. Demographic and clinical data were collected from patients' electronic medical records. Data analysis performed using JASP. The total number cases were 154, we only included cases with available 12‐lead ECG and complete medical records (60% of patients). Pre,post‐procedure and pre‐discharge 12‐lead ECGs were interpreted.

Loss of SQ was diagnosed if more than 3 leads ( I,aVL,V5,V6) showed an initial R. In cases of loss of Q in V 5/6 and presence in I /aVL, we decided to base the diagnosis according to prominence of Q waves present. In case of baseline LBBB and LVH, Q waves were not accounted for as pathological.

**Results:** A total of 97 patients (44% females) who underwent TAVR in our institution were included. Baseline characteristics and conduction disease are shown in Table‐1. 26% of patients developed nLBBB or required PPI. Loss of Q wave in lead I was less commonly observed compared to V5 or V6 and it showed a +LR 5.6 (P0.018) for developing nLBBB or PPI. Pre‐procedure loss of septal Q waves was significanlty associated with post‐procedure BBB/CHB (+LR13.5 (P 0.009) and pre‐discharge nBBB ( +LR 8.7 (P 0.013). There was no statistically significant difference between valve types and risk of PPI/nBBB.

**Conclusions:** Among patients with "normal" or subtle ECG findings, loss of SQ increased the likelihood of nBBB and PPI post‐TAVR. A large scale study is necessary to illustrate this association and assist physicians to further risk stratify patients undergoing TAVR.

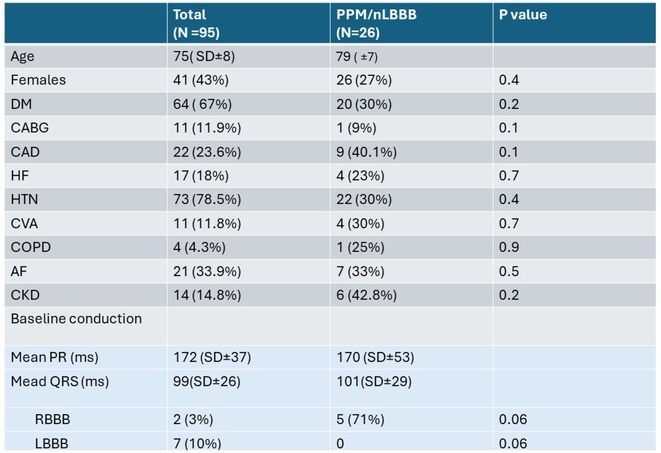

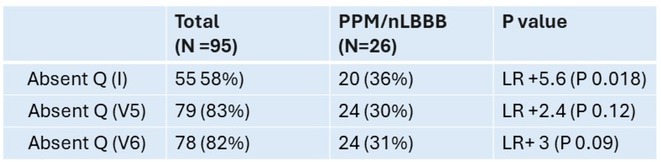

## 5 CARDIAC SYMPATHETIC NERVOUS EXCITATION MONITORING DURING PULMONARY VEIN ISOLATION BY USING SKIN POTENTIAL RESPONSE

### 5.1 **HIDEYUKI AOKI**, YUICHI HORI, HIROTSUGU SATO, REIKO FUKUDA, SHIRO NAKAHARA

#### 5.1.1 Dokkyo Medical University Saitama Medical Center, Koshigaya, Japan

**Introduction:** Vagal responses during pulmonary vein isolation (PVI) have been reported, suggesting cardiac neuromodulation in atrial fibrillation (AF) patients. On the other hand, the sympathetic nervous system's response during PVI is unclear and the precise method to evaluate the cardiac sympathetic reflex is still not established. Skin potential response; SPR occurs during sympathetic excitation and may be useful to represent cardiac sympathoexcitation during PVI.

**Methods:** Thirty‐four persistent AF patients were enrolled and the SPR was monitored during balloon‐based ablation (Cryoballoon ablation 17case, Hot balloon ablation 17case) of each PV. The SPR was measured by the difference in two skin potentials recorded from skin electrodes. The reduction in the activation recovery interval (ARI), suggesting sympathetic excitation, was examined from the ventricle unipolar electrogram of the great cardiac vein recorded from an electrode catheter placed inside the coronary sinus.

**Results:** In total 34% of the PV ablation showed an SPR, (LSPV = 47%, RSPV = 47%, LIPV = 21%, RIPV = 21%). Regarding the hemodynamic changes during the PV ablation, PV ablation with SPR showed a significant increase in HR (SPR (+) vs. SPR (‐) = 16.0 ± 15.1 vs 1.3±5.7%, p<0.01) and systolic BP (7.0 ± 6.2 vs 0.3±6.6%, p<0.01), and a significant shortening of ARI (‐7.0 ± 5.7 vs ‐0.7±2.9%, p<0.01). The AF recurrence within 1 year after the balloon‐based ablation was documented in 17.6% of the enrolled patients. The patients that showed SPR 2 or more times (SPR ≧ 2 times) during the 4PV ablation, showed 12.5% AF recurrence, while the patients that had an SPR less than 2 times (SPR < 2) was 22.2% (p = 0.467).

**Conclusions:** The SPR was strongly related to cardiac sympathoexcitation during PVI. The SPR was suggested to represent the cardiac sympathetic reflex during PV antrum ablation, which may lead to an investigation of the cardiac sympathetic neuromodulation during PVI.

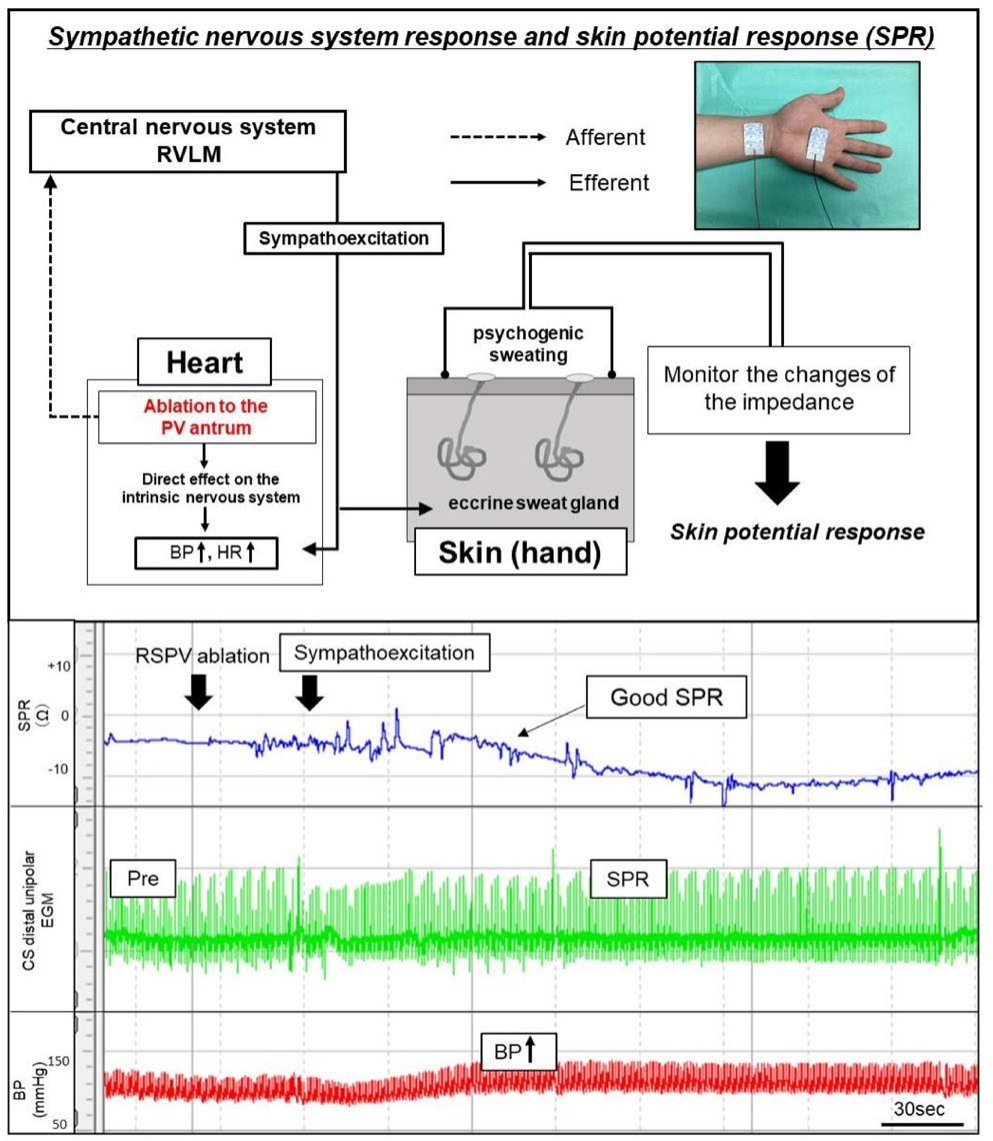

## 6 PRELIMINARY EVIDENCE ON EV‐ICD WITH CONCOMITANT MICRA

### 6.1 **ALFONSO ARANDA HERNANDEZ** ^1^, G. STUART MENDENHALL^2^, MATTHEW HOFFMAN^1^, VLADIMIR NIKOLSKI^1^, ANNA KAROS^1^

#### 6.1.1 ^1^Medtronic, Mounds View, MN,^2^Scripps Memorial Hospital, La Jolla, CA

**Introduction:** The Aurora™ extravascular ICD, with the lead substernal and out of the vasculature, reduces certain risks associated with transvenous ICDs and enables the use of ICD therapies for patients whose vasculature does not support the placement of transvenous leads. For Aurora patients who require pacing therapy, a Micra™ leadless pacemaker is being studied as a potential option for pacing support without placing leads in the vasculature. However, the interactions between Aurora and Micra remain unexplored.This research aims to provide insights into the interactions between Aurora and Micra using both in vitro and in vivo models.

**Methods:** Saline tank experiments were conducted simulating Micra and Aurora's concomitant locations in human physiology to evaluate the effect of Micra distance and orientation on the pacing pulses sensed by Aurora and assess the risk of VF undersensing and oversensing. Anesthetized acute animal experiments (3 sheep and 2 pigs) were also performed to corroborate the saline tank results and evaluate the effect of ATP and defibrillation therapies on pacing thresholds.Key assessments involved checking for VF undersensing or VF oversensing due to Micra's pacing at pacing amplitudes of 1.5V, 3V and 5V with pulse widths of 0.24ms, 0.40ms and 1ms. Also, Micra post‐shock pacing success and pacing thresholds stability for shock energies of 40J, 67J, 79J and 126J.

**Results:** Pacing pulse amplitudes sensed by Aurora were dependent on the distance between Micra and Aurora. No VF undersensing occurred at any pacing amplitude for pulse widths of 0.24 ms or less. Aurora did not double count pacing pulses with Micra programmed at maximum output. Finally, no increase in the pacing thresholds was observed as a result of ATP or consecutive shocks.

**Conclusions:** Our findings suggest that the use of Aurora with a concomitant Micra does not negatively impact the performance of either device in vitro or in acute animal models under certain conditions.Despite the encouraging results, the study's limitations, including the absence of human data and a small sample size, necessitate further research to generalize these findings.

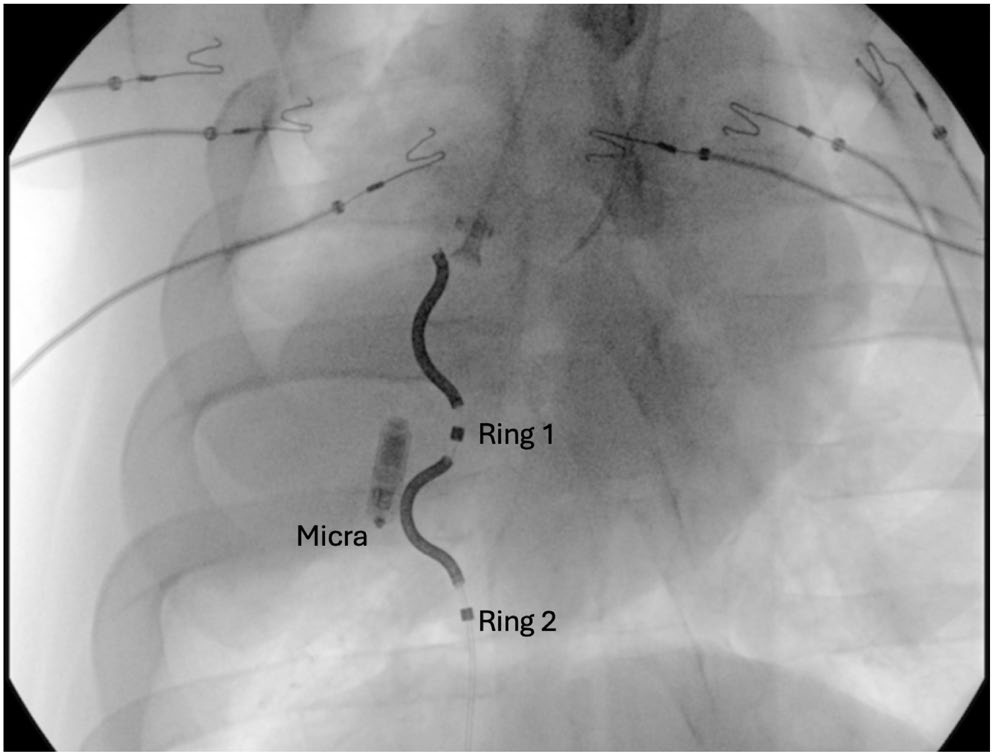

## 7 PERFORMANCE OF SLEEP APNEA DETECTION ALGORITHM USING INSERTABLE CARDIAC MONITOR IN PATIENTS WITH HEALTHY AND UNHEALTHY HEART RATE VARIABILITY INDICES

### 7.1 **JONATHAN ARIYARATNAM** ^1^, GAUTHAM RAJAGOPPAL^2^, JOHN FITZGERALD^1^, KADHIM KADHIM^1^, DOMINIK LINZ^1^, YONG CHO^2^, DANNY ECKERT^3^, PRASH SANDERS^1^

#### 7.1.1 ^1^University of Adelaide, Adelaide, Australia,^2^Medtronic, Inc., Minneapolis, United States of America, Minneapolis, MN,^3^Flinders University, Adelaide, Australia

**Introduction:** Sleep disordered breathing (SDB) is implicated in the development of AF. However, screening for SDB is suboptimal due to significant night‐to‐night variability. Implantable cardiac monitors (ICM) are frequently used for AF management. We used an automated sleep apnea detection algorithm to diagnose SDB severity using ICMs.

**Methods:** We developed a heart rate‐based sleep apnea detection algorithm using LINQ ICMs. The Sleep Disordered Heart Rate (SDHR) index was calculated as the average number of SDHR events detected by the algorithm per hour of sleep. Consecutive patients with ICMs undergoing overnight PSG were recruited. During PSG, SDHR was simultaneously assessed from the ICM. Agreement between AHI and SDHR was assessed by means of Bland‐Altman test. The HRV index was computed as standard deviation of R‐R intervals (SDNN) measured from ICM ECG with SDNN indices <100 msec being considered as unhealthy range.

**Results:** 18 patients with implanted ICM were enrolled to undergo a sleep study. Mean age of the cohort was 72.5±7.96. Ten patients had HRV indices in the healthy range (160.9±33.8 msec) and 8 patients in the unhealthy range (84.6±5 msec). In patients with healthy HRV, the overall agreement between SDHR index and AHI was good with a mean difference of 4.7±6.6 (Figure 1a). Using an AHI cutoff of 15 for diagnosis of SDB, 8 patients (80%) were correctly diagnosed using the SDHR index with underestimation of the AHI in 2 patients (20%) (Figure 1b). For patients with unhealthy HRV, the overall agreement between the two methods was less good (mean difference 12.9±27.2) with underestimation of the AHI in 3 patients (37.5%) and overestimation in 1 patient (12.5%) (Figure 1c).

**Conclusions:** The SDHR index calculated from overnight ICM ECG data demonstrated good agreement with AHI from PSG for patients with HRV in the healthy range. Novel ICM detected SDB represents a potentially effective screening strategy for SDB in patients with AF.

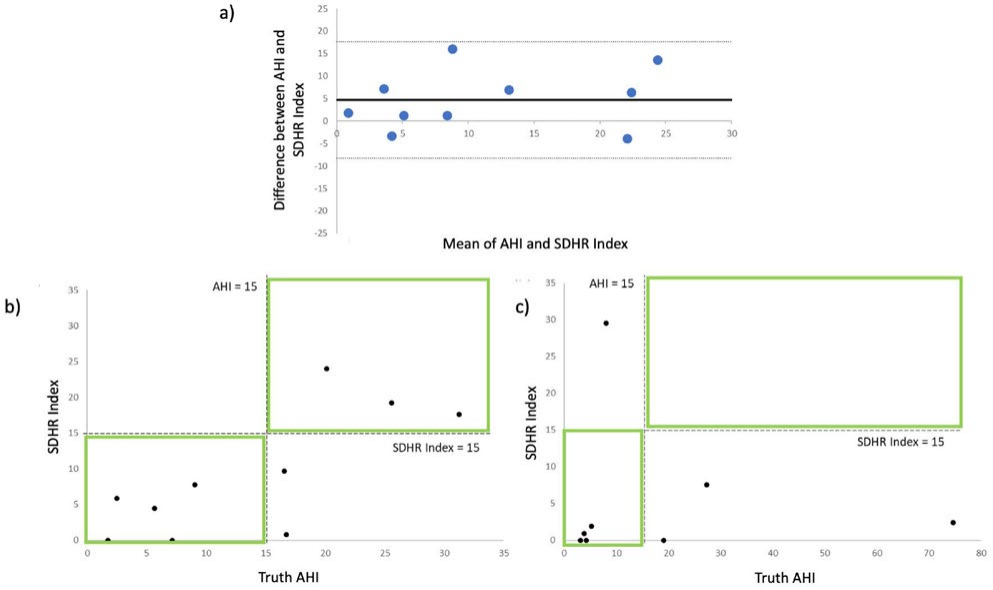

## 8 ELECTROCARDIOGRAPHIC MANIFESTATIONS OF HOSPITALIZED ADULT PATIENTS WITH CORONAVIRUS DISEASE 19 (COVID‐19): UP DCVM ECG STUDY

### 8.1 **TAM ADRIAN AYA‐AY**, FELIX EDUARDO PUNZALAN, PAUL ANTHONY ALAD, KAYE EUNICE LUSTESTICA, NIGEL JERONIMO SANTOS, JAIME ALFONSO AHERRERA, ELMER JASPER LLANES, GISELLE GERVACIO, EUGENE REYES, JOHN AÑONUEVO

#### 8.1.1 University of the Philippines‐Philippine General Hospital, Manila City, Philippines

**Introduction:** COVID‐19 has been reported to cause cardiac injury that can manifest in the electrocardiogram (ECG). This study aims to describe the cardiovascular and electrocardiographic profile of adult patients with COVID‐19.

**Methods:** This was a cohort study involving adult patients with confirmed COVID‐19 from June 2021 to June 2022. Clinical profiles and 12L‐ECG tracings were retrieved from electronic medical records, and subsequently adjudicated by three cardiologists. Descriptive analysis was used to summarize the cardiovascular and electrocardiographic profile among these patients.

**Results:** A total of 998 COVID‐19 patients (mean age 54; men 51.2%) were included. The most common comorbidities were hypertension, diabetes, and dyslipidemia. Majority of patients had severe COVID‐19 infection (53.7%). On admission, 6.4% needed intubation and 14.6% died. The most common ECG abnormalities on admission were non‐specific ST‐T wave changes (41.8%) and sinus tachycardia (22.8%). Other findings were ST segment depression (2.91%), T wave inversion (1.70%), ST segment elevation (2.71%), premature ventricular complexes (4.31%).

**Conclusions:** In our study, the baseline electrocardiographic profile of patients with COVID‐19 were mostly normal. ECG patterns related to ischemia and arrhythmias were found to be infrequent. Nevertheless, a follow‐up 12L‐ECG may still be noteworthy in patients with high risk factors and signs of deterioration.

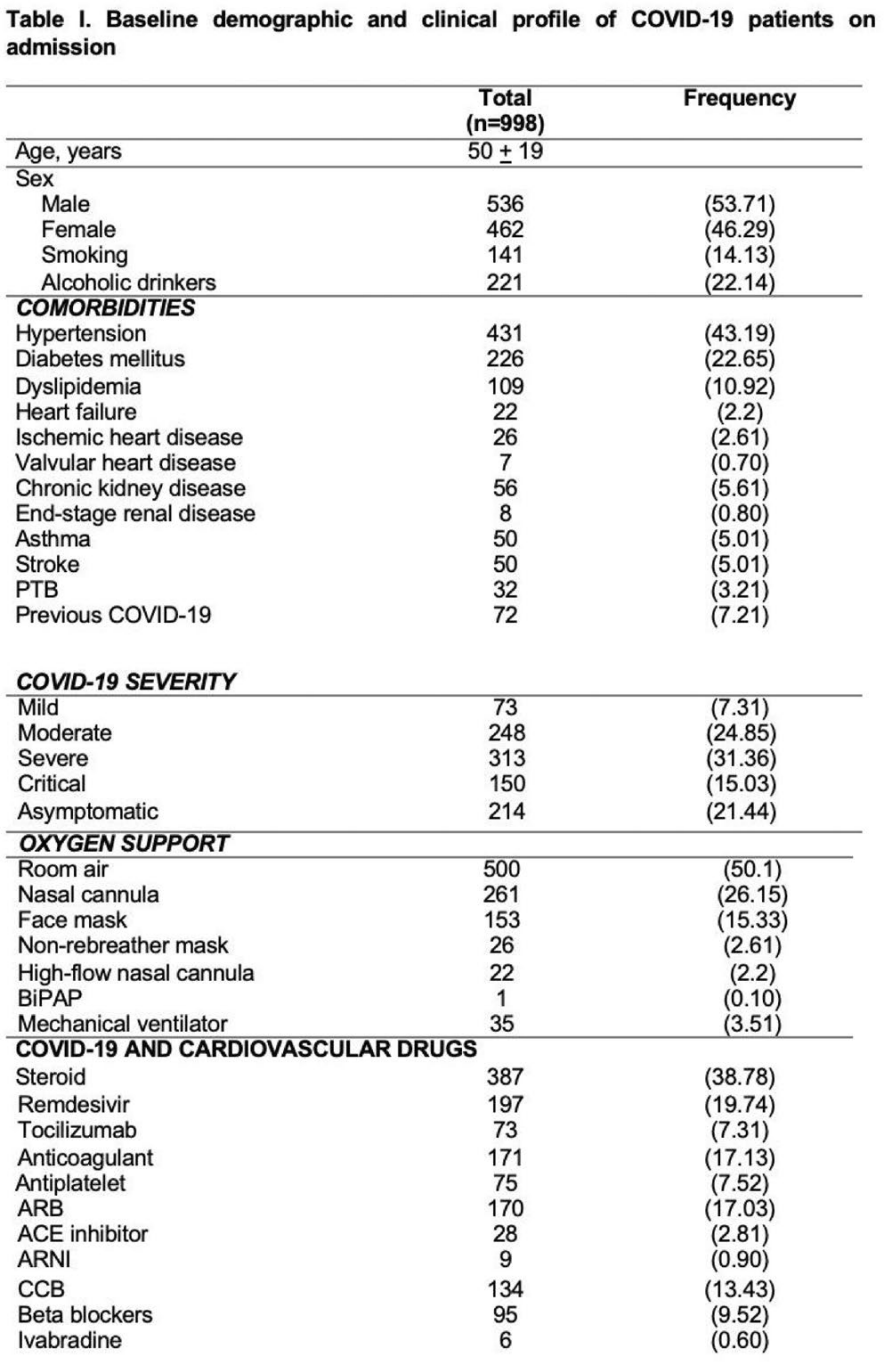

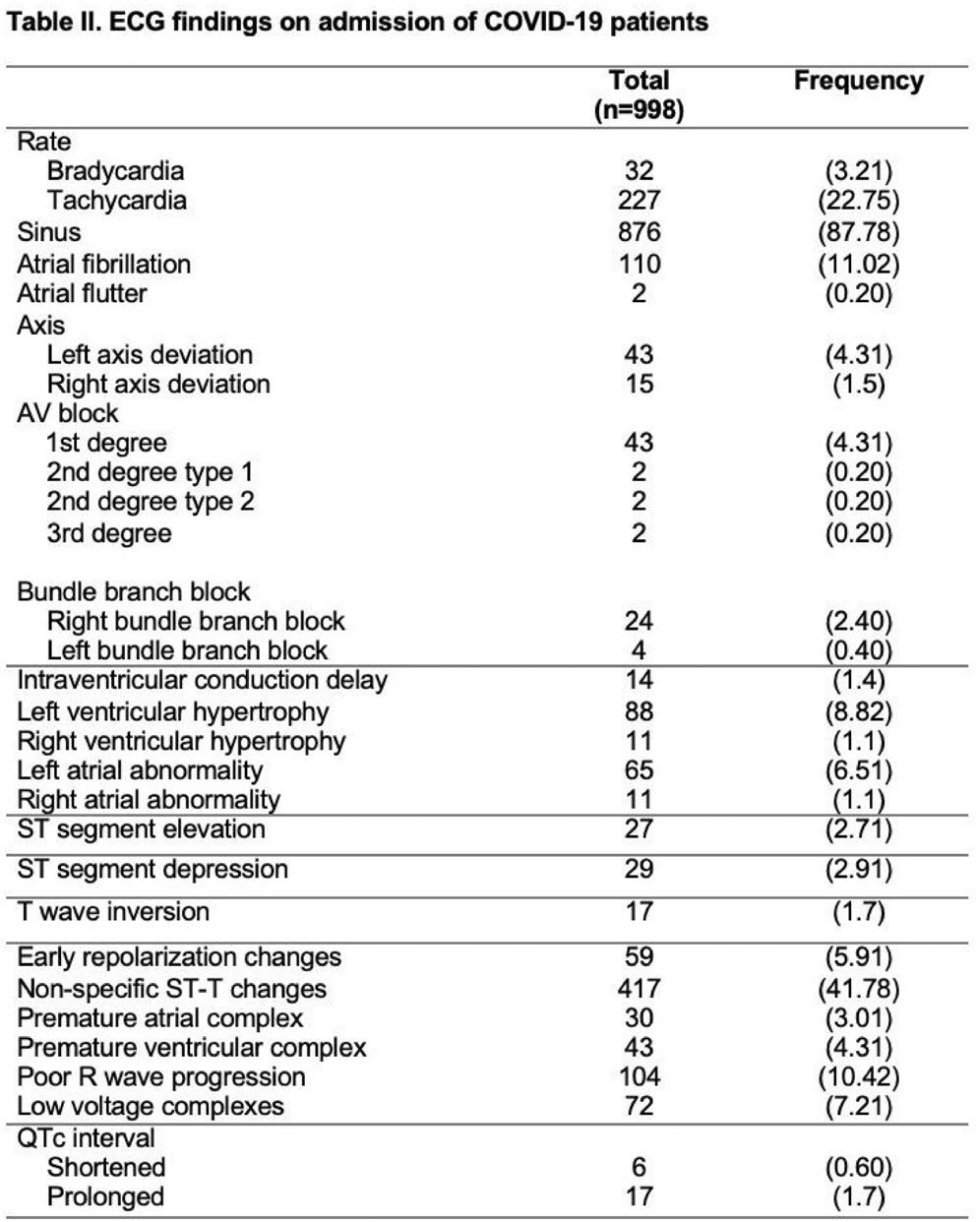

## 9 THE PROGNOSTIC SIGNIFICANCE OF EXERCISE‐INDUCED RIGHT BUNDLE BRANCH BLOCK: A SYSTEMATIC REVIEW

### 9.1 **MAYA QUROTA AYUN**, BAGUS HERY KUNCAHYO

#### 9.1.1 Ngudi Waluyo General Hospital, Kab. Blitar, Indonesia

**Introduction:** Exercise‐Induced Right Bundle Branch Block (EI‐RBBB) during exercise stress test is an uncommon electrocardiographic finding with debatable clinical significance. Although information about EI‐LBBB is available and written in the guidelines, fewer studies of EI‐RBBB have been published. This paper aims to identify the prognostic relevance of EI‐RBBB to determine the appropriate steps for better management.

**Methods:** A systematic search in 3 databases, Pubmed, Scopus, and ScienceDirect using the keywords "Right Bundle Branch Block OR RBBB AND Exercise stress test AND prognostic OR clinical significance". A total of 457 articles meet the keywords. The inclusion criteria were: 1) study including normal sinus patients undergoing exercise stress test and there was data about EI‐RBBB; 2) Publication between 2000‐2024; 3) Fulltext in English.

**Results:** Three studies were included. A total of 11.315 patients undergoing exercise stress test records were identified. Two studies were prospective with the observation period being 2‐14.2 years. All of the studies have shown EI‐RBBB correlated with all‐cause mortality (OR 1.5 [CI, 1.1 to 1.9]; P < 0.017; HR 1.13 (95% confidence interval 0.51 to 2.5, p < 0.75); p < 0.021). One study has shown EI‐RBBB was an independent risk for cardiovascular death HR 1.57 (95% confidence interval 0.51 to 4.8, p < 0.4). Patients with EI‐RBBB also showed qualitatively and quantitatively lower EF (p < 0.001) and larger infarction and scar burden (p < 0.001) based on perfusion imaging.

**Conclusions:** The number of studies that examined the clinical significance of EI‐RBBB is still limited. The studies identified were diverse in design and the results were conflicting. This should be a focus for future research and determination in guidelines. Further evaluation may still be warranted depending on the clinical scenario to exclude coronary abnormality and assess ventricular function. Consider doing an echocardiogram, perfusion imaging, and/or angiography for better prognosis and management.

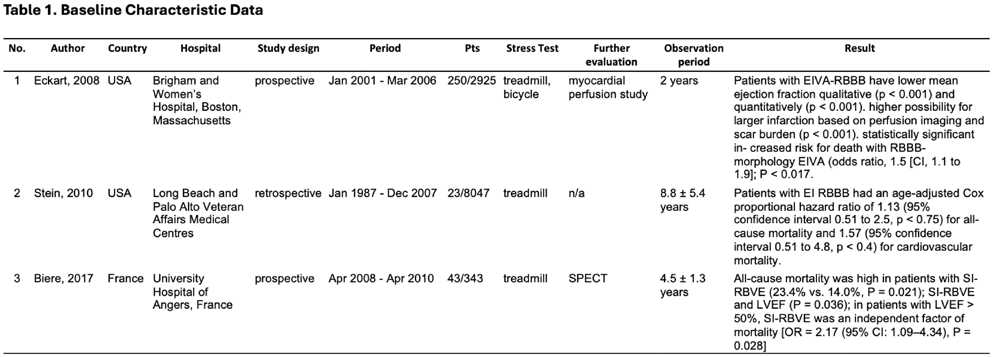

## 10 CLINICAL MANIFESTATION AND OUTCOME OF IDIOPATHIC VENTRICULAR FIBRILLATION IN PEDIATRIC PATIENTS : A MULTICENTER STUDY

### 10.1 **SEUNG MIN BAEK** ^1^, JAE SUK BAEK^2^, MI KYOUNG SONG^1^, JOOWON LEE^3^, JA KYOUNG YOON^4^, MI JIN KIM^2^, JAE‐SUN UHM^4^, JUNE HUH^5^, CHANG SIN KIM^4^, AH YOUNG KIM^4^, SO YUN JUN^1^, EUN JUNG BAE^1^

#### 10.1.1 ^1^Seoul National University Children's Hospital, Seoul, Korea, Republic of,^2^University of Ulsan College of Medicine, Seoul, Korea, Republic of,^3^Seoul National University Bundang Hospital, Bundang, Korea, Republic of,^4^Severance Cardiovascular Hospital, Yonsei University College of Medicine, Seoul, Korea, Republic of,^5^Samsung Medical Center, Sungkyunkwan University school of Medicine, Seoul, Korea, Republic of

**Introduction:** Reports on idiopathic ventricular fibrillation (IVF) in the pediatric population are limited. We investigates the clinical manifestations and characteristics of IVF in pediatric patients from a nationwide Korean multicenter cohort study.

**Methods:** We retrospectively analyzed data from 41 patients diagnosed with IVF across six hospitals from 2001 to 2022. End‐QRS notch or slur on the down slope of a R wave with amplitude greater than 0.1mV in two or more contiguous leads, excluding V1~3 was defined as early repolarization (ER) pattern. Life‐threatening arrhythmic events (LAE) were defined as ventricular tachycardia (VT), ventricular fibrillation (VF), appropriate implantable cardioverter defibrillator (ICD) shock, sudden cardiac death (SCD), or aborted cardiac arrest (ACA). Factors influencing the occurrence of LAE were statistically assessed.

**Results:** The mean age at presentation was 16.0 ± 2.3 years, with a significant male predominance (92.7%). The most common precipitating factor was exercise (10 cases, 24.4%), followed by rest (8 cases, 19.5%), sleep (5 cases, 12.2%), and emotional arousal (4 cases, 9.8%). Twelve (29.3%) patients had a family history of SCD or ACA. Early repolarization (ER) pattern was observed in 9 (22.0%) patients. No patient had VT/VF on treadmill test or drug provocation test. During the mean follow‐up of 4.9±4.0 years, 26 patients (63.4%) experienced LAE (appropriate ICD shock: 20 [among 36 patients with ICD implantation], ACA: 4, documented VF/VT: 1, SCD: 1). Cox regression analysis indicated that the presence of ER pattern significantly increased the risk of LAE (HR: 3.468, 95% CI:1.096‐10.967, p = 0.034). Additionally, Kaplan‐Meier analysis showed that pateints with ER patern had a significantly lower event‐free survival compared to patients without ER pattern (Log‐Rank X^2^ =15.875, P<0.001, Figure).

**Conclusions:** Pediatric patients with IVF patients, despite their heterogeneity, have an elevated risk of recurrent ventricular arrhythmias, necessitating aggressive management. Particularly, patients with an ER pattern are at a heightened risk for LAE.

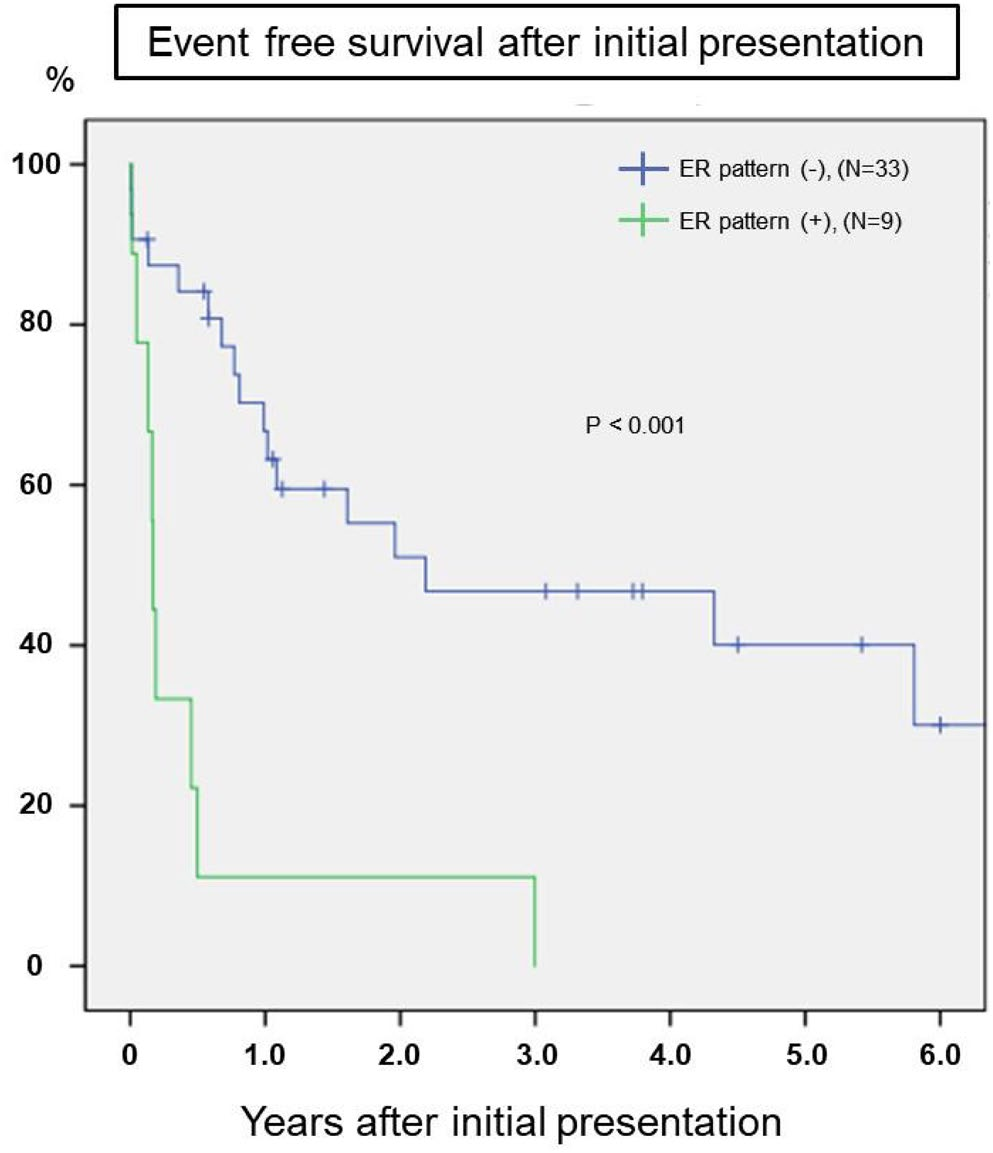

## 11 USE OF TUMESCENT LOCAL ANAESTHESIA AND DEEP SEDATION PROTOCOL AS AN ALTERNATIVE TO GENERAL ANAESTHESIA FOR THE IMPLANTATION OF SUBCUTANEOUS IMPLANTABLE CARDIOVERTER DEFIBRILLATOR

### 11.1 **JAMIE CHAM** ^1,2^, KEN CHO^1^, DANIEL AKRAWI^1^, JONATHAN HOOPER^1^, KAT BATE^1^, TUAN NGUYEN^1,2^, HANY DIMITRI^1^, ANDREW HOPKINS^1^, ADAM LEE^1^

#### 11.1.1 ^1^Liverpool Hospital, Sydney, Australia,^2^Campbelltown Hospital, Sydney, Australia

**Introduction:** Sub‐cutaneous implantable cardioverter defibrillator (S‐ICD) implant has traditionally been performed under general anaesthesia (GA). However, lack of GA availability has prompted consideration of alternate strategies. Use of tumescent local anaesthesia (TLA) has been described [1]. We present a case series (n=13) of de‐novo S‐ICD implantation using TLA with deep procedural sedation.

**Methods:** 14 S‐ICDs were implanted using a TLA and deep sedation protocol. TLA was composed of 0.9% sodium chloride (1L), 1% lidocaine (50mL), epinephrine (1mg of 1/1,000), and 8.4% sodium bicarbonate (12.5mL). The sedation protocol was an age and weight adjusted fentanyl (15‐50microg/hr) and midazolam (3‐10mg/hr) infusion, with additional boluses as required.

**Results:** All implants were successful (n=14/14). Average age of patients was 46 +/‐ 14.7 years. Average body mass index was 29.8 +/‐ 6.2kg/m2. Average midazolam requirement was 19 +/‐ 5.4mg, and average fentanyl requirement was 216+/‐ 51microg. All patients had no recall of intraprocedural pain. Average visual analogue pain score post operatively was 3/10 (+/‐ 2). There were no immediate procedural complications, though 3/14 patients required administration of flumazenil for benzodiazepine reversal in recovery. The average HV impedance was 42 +/‐ 11 ohms (range 30‐73). Defibrillator threshold testing (DFT) was conducted in one patient, and was poorly tolerated. In the 6 months prior to this protocol's introduction, 4 S‐ICDs were implanted, compared to 15 in the following 6 months.

**Conclusions:** In settings of low GA availability, TLA and sedation provides a safe and effective option for S‐ICD implantation (though without DFT), facilitating increased S‐ICD implantation rates.

References: 1. Romero, J., et al., Tumescent local anesthesia for subcutaneous implantable cardioverter‐defibrillator implantation: An alternative for general anesthesia. HeartRhythm Case Rep, 2021. 7(5): p. 286‐291.

## 12 AVEIR VR IMPLANTATION IN SMALL HEARTS ‐ A RETROSPECTIVE COHORT STUDY HIGHLIGHTING SPECIAL TECHNIQUES USED

### 12.1 **CHUN YIN, VICTOR CHAN** ^1^, YUET WONG CHENG^2^, YAT SUN, JOSEPH CHAN^3^, CHIN PANG CHAN^3^, LING LING IP^1^, YEE MAN, ANITA POON^3^, TSZ KIN, MARK TAM^4^

#### 12.1.1 ^1^Tuen Mun Hospital, Tuen Mun, Hong Kong,^2^Queen Elizabeth Hospital, Kowloon, Hong Kong,^3^Prince of Wales Hospital, Shatin, Hong Kong,^4^The Chinese University of Hong Kong, Shatin, Hong Kong

**Introduction:** The implantation of Aveir VR pacemaker requires a large‐calibre delivering system. Compared to Micra, the longer device length in Aveir VR may hinder its manoeuvrability in patients with small body build. This study aims to explore the incidence and predicting factors for difficult Aveir VR implantation, and special techniques employed in successful implantation.

**Methods:** This study is a retrospective analysis comparing usual and difficult Aveir VR implantation groups. Consecutive patients undergoing Aveir VR implantation from 3/2023 to 4/2024 in 4 centres in Hong Kong were included. Difficult Aveir VR implantation was defined as a failure to deliver device into right ventricle by merely using the Aveir delivery system via standard transfemoral approach. Patient's demographics and comorbidities were analysed for association with difficult implantation using Student t‐test and Pearson's chi‐square test.

**Results:** 186 consecutive patients were included in this analysis. Comparing with Leadless II Phase I study (BMI 28.7 ± 6.8 kg/m^2^), this cohort was of smaller body size (mean body weight 58.2 ± 10.8 kg, body height 159.2 ± 8.7 cm, body surface area (BSA) 1.60 ± 0.17 m^2^, body mass index (BMI) 22.9 ± 3.7 kg/m^2^). Implantation success rate was 98.9%. 8 cases (4.3%) were classified as difficult implantation, which was associated with lower body height, body weight and BSA (p = 0.002, p = 0.011 and p = 0.004 respectively), but not BMI (p = 0.257). A BSA cut‐off of 1.45 m^2^ identified difficult implant with 80.2% sensitivity and 85.7% specificity. Among the 8 difficult cases, Aveir VR was successfully implanted with special techniques in 7 patients (5 with snare‐assisted technique, 2 with trans‐jugular venous approach). For the remaining case, upon patient's preference, conventional pacemaker was used without attempt of special technique.

**Conclusions:** Difficult Aveir VR implantation was occasionally encountered especially in patients of small body size. These patients may be challenging for new implanters. Special techniques such as snare‐assisted technique and trans‐jugular venous approach can be employed to achieve a high implant success rate.

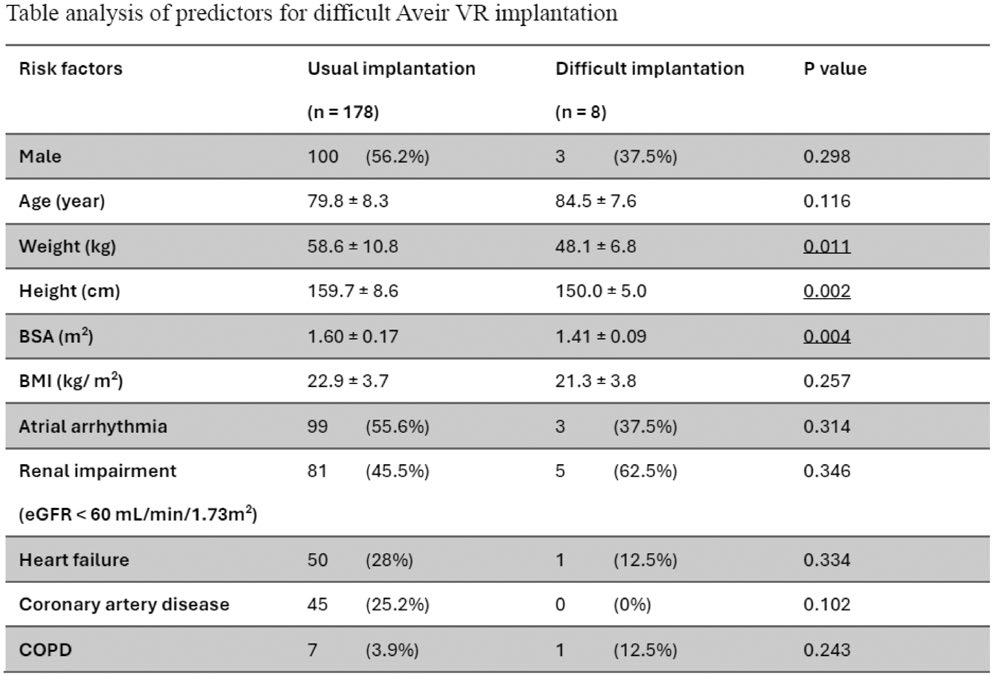

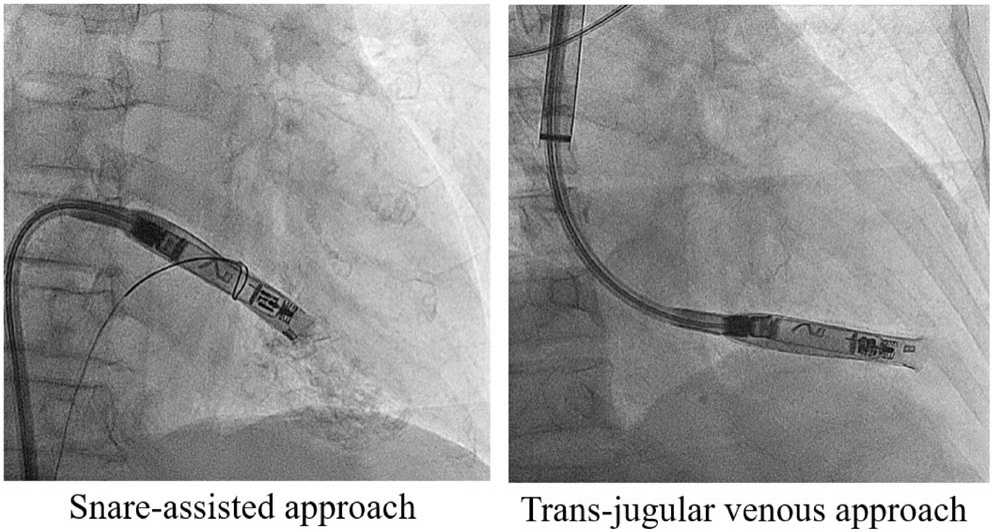

## 13 CARDIAC DYSFUNCTION IN PATIENTS POST PERMANENT PACEMAKER IMPLANTATION IN A SINGAPORE PAEDIATRIC CARDIAC CENTRE

### 13.1 **JIAHUI CHARMAINE CHAN**, DYAN ZHEWEI ZHANG, SREEKANTHAN SUNDARARAGHAVAN, JONATHAN TZE LIANG CHOO, WAN TING CHAN, NURHAFIZAH BINTE ABDUL AZIZ, MONIKA K KOTECHA, TENG HONG TAN

#### 13.1.1 KKH, Singapore, Singapore

**Introduction:** Permanent cardiac pacing may have deleterious effects on left ventricular (LV) function. This is more pronounced in children, who are subject to decades of pacing. This study aimed to investigate the characteristics of patients with cardiac dysfunction post permanent pacemaker implantation (PPI).

**Methods:** Paediatric patients under 18 years old who were followed up at our centre post PPI from January 1994‐April 2024 were identified. Patients with normal cardiac function prior to PPI, and any cardiac dysfunction (LVEF <50%) on follow up were included.

**Results:** 11 out of 53 PPI patients (20%) had cardiac dysfunction on follow up. The majority of patients had post‐operative complete heart block (CHB) (7/11; 63.6%), followed by congenital CHB (3/11; 27%). 83% of patients had an epicardial system. All patients had chronic ventricular pacing; 8 were RV paced, 1 LV paced, and 2 septal paced. Mean follow‐up period was 16.3 years (1.25 to 30 years). On average, cardiac dysfunction was first identified 10 years post PPI (3 months‐21 years). Mean age at diagnosis was 13.4 years (1.5‐21 years old). 9/11 patients had pacing‐induced cardiomyopathy (PIC). 1 patient had left circumflex (LCx) artery stenosis due to compression from calcification from an abandoned epicardial lead. 1 patient had dilated cardiomyopathy from other cause. 7 patients were treated with heart failure medications. 5 patients had a change in pacing strategy: 3 converted to biventricular (BiV) pacing, 1 patient converted from RV to LV pacing, 1 patient converted to multisite pacing of single ventricle. 3 patients had full recovery of cardiac function. Of these, 2 were associated with change in pacing strategy while the patient with LCx stenosis underwent coronary stenting. There were 3 deaths in this study period, all had underlying congenital heart disease.

**Conclusions:** PIC is a major cause of cardiac dysfunction post PPI. Postoperative CHB is a risk factor for PIC, and may be associated with higher mortality. Change in pacing strategy, and not heart failure medications, may reverse cardiac dysfunction. Reversible causes of reduced cardiac function should always be considered.

## 14 HIGH SKIN SYMPATHETIC NERVE ACTIVITY IN NOISE ASSOCIATED VENTRICULAR ARRHYTHMIAS

### 14.1 **CHAO‐YI CHEN** ^1^, CHIA‐HAO KUO^1^, RUO‐YUN SHIH^1^, PIN‐CHIEH HUANG^1^, XIN‐HUI CHEN^1^, YI‐HSIUNG LIN^1^, WEN‐TER LAI^1^, SHIEN‐FONG LIN^2^, BIN‐NAN WU^3^, WEI‐CHUNG TSAI^1,4^

#### 14.1.1 ^1^Division of Cardiology, Department of Internal Medicine, Kaohsiung Medical University Hospital, Kaohsiung Medical University, Kaohsiung, Taiwan,^2^Institute of Biomedical Engineering, National Chiao‐Tung University, Hsin‐Chu, Taiwan,^3^Department of Pharmacology, College of Medicine, Kaohsiung Medical University, Kaohsiung, Taiwan,^4^Department of Internal Medicine, School of Medicine, College of Medicine, Kaohsiung Medical University, Kaohsiung, Taiwan

**Introduction:** Long‐term noise exposure leads to disease through the mechanism of autonomic nervous system (ANS) activation, causing arrhythmias, certain cardiovascular disease and mortality. We use neuECG, a reliable and reproducible non‐invasive method for ANS monitoring, to simultaneously record electrocardiogram (ECG), skin sympathetic nerve activity (SKNA), and heart rate variability (HRV) to study autonomic function in noise‐exposed (NOE) mice. We hypothesize that noise exposure induces ventricular arrhythmias (VA) via dysregulation of the ANS, including changes in SKNA and HRV in mice.

**Methods:** We used C57BL/6 (B6) mice divided into four groups: (1) NOE +vehicle, (2) NOE +6‐hydroxydopamine (6‐OHDA), (3) control (CTL) +vehicle, (4) CTL +6‐OHDA. The NOE model was created with B6 mice exposed to broadband noise 20‐20k Hz, 85dB for 28 days. neuECG used electrodes in the traditional lead I configuration for simultaneous ECG, SKNA, and HRV measurements. The cold pressor test (CPT) was used to induce sympathetic activation. All measurements were performed with three phases including baseline, CPT, and recovery, each lasting 3 minutes. VA was defined as spontaneous ventricular premature beats (VPB). SKNA burst ratio was defined as SKNA bursts within the first 0.2 seconds before VPB out of 100 heartbeats.

**Results:** In the within‐group comparison, SKNA increased significantly in NOE +vehicle (p=0.003) and NOE +6‐OHDA group (p=0.028) in CPT phase, while no significant increase was observed in both CTL groups. When comparing between groups, NOE +vehicle had higher SKNA than NOE +6‐OHDA group in all phases (p≤0.019). NOE +vehicle had higher SKNA than CTL +vehicle group in all phases (p≤0.037). Large SKNA bursts in NOE +vehicle mice preceded the onset of VA (SKNA burst ratio=0.81). During CPT, the NOE groups showed a significant increase in VA (p=0.0047), whereas the CTL groups had no VA. No HRV differences were observed between four groups.

**Conclusions:** Noise exposure increases SKNA and induces the occurrence of VA, which is associated with sympathetic activation and can be inhibited by 6‐OHDA. ANS dysregulation partially explains the mechanism of noise‐induced VA.

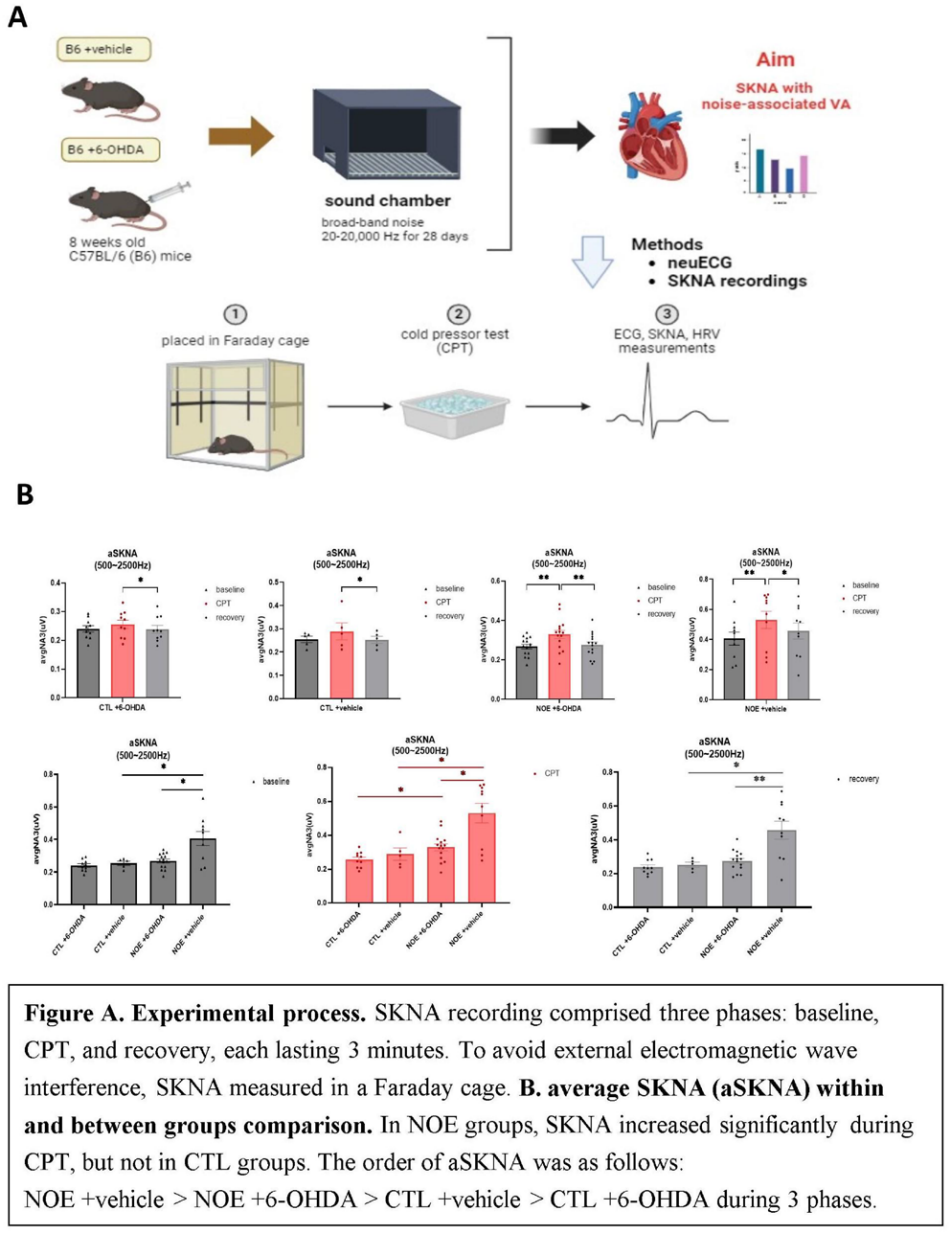

## 15 THE CORRELATION BETWEEN CHANGES IN BODY MASS INDEX AND THE LONG‐TERM RISK OF CARDIAC CONDUCTION BLOCK IN HYPERTENSIVE POPULATIONS: EVIDENCE FROM THE KAILUAN COHORT STUDY

### 15.1 GUANZHI CHEN

#### 15.1.1 Peking Union Medical College, National Center for Cardiovascular Diseases, Fuwai Hospital, Beijing, China

**Introduction:** Cardiac conduction block (CCB) is a common bradyarrhythmia that significantly increases cardiovascular and mortality risk. Each 5 kg/m^2^ increase in BMI raises the CCB risk by over 20%. While high BMI is linked to higher CCB risk in the general population, no studies have examined weight changes' impact on CCB risk in hypertensive patients. We hypothesized that BMI changes affect CCB risk in individuals with hypertension. To test this, we used data from the Kailuan Study cohort in China to explore BMI changes and CCB incidence in hypertensive patients.

**Methods:** The follow‐up period began immediately after the 2010 health examination, during which a total of 38,907 observations were made, until a CCB event occurred or December 31, 2021. Poisson regression within a generalized linear model was used to calculate the relative risk (RR) and 95% confidence interval (95% CI) for CCB that was associated with BMI changes. Participants with normal BMIs in both 2006 and 2010 were used as the reference group.

**Results:** We studied 38,907 individuals with a mean age of 56.11 ± 11.31 years. The median duration of follow‐up was 10.06 years. Poisson regression analysis of the generalized linear model, after adjustment for confounding factors, showed that the Overweight/obesity‐to‐normal weight, Normal weight‐to‐overweight/obesity, and Overweight/obesity‐to‐overweight/obesity groups had higher risks of CCB (relative ratio: 1.33, 95% confidence interval: 1.32‐1.34; 1.76, 1.73‐1.79; and 1.74, 1.70‐1.77; respectively) vs. the Normal weight‐to‐normal weight group.

**Conclusions:** In hypertensive populations, changes in BMI are closely linked to the incidence of CCB. Patients with high BMI have an increased CCB risk. Reducing BMI from overweight/obese to normal lowers but doesn't eliminate this risk. Increasing BMI from normal to overweight/obese raises CCB risk more than the reduction achieved by losing weight. Notably, the CCB risk increase from gaining weight is higher than maintaining a high BMI continuously.

## 16 THE IMPACT OF PULMONARY VEINS ANATOMY ON THE CRYOKINETICS DURING CRYOBALLOON ABLATION FOR ATRIAL FIBRILLATION

### 16.1 **CHENG‐HUNG CHIANG** ^1,2^, KUO‐MING YANG^1^, CHUN‐WANG CHIOU^1,2^, HSIANG‐CHIANG HSIAO^1,2^, TUNG‐CHEN YEH^1^

#### 16.1.1 ^1^Kaohsiung Veterans General Hospital, Kaohsiung City, Taiwan,^2^School of Medicine, National Yang Ming Chao Tung University, Taipei City, Taiwan

**Introduction:** The effectiveness of cryoballoon ablation (CBA) for atrial fibrillation (AF) depends on the anatomy of pulmonary veins (PVs). This study is aimed to evaluate the impact of PV ovality index (OI) and PV ostium area (OA) on cryokinetic parameters during PV isolation with CBA.

**Methods:** During January 2021 and December 2023, 120 consecutive patients in a tertiary hospital were enrolled. Among them, the average age was 58.63±9.65 years, 12 (10%) patients were female, and 18 (15%) patients were in persistent AF status. Prior to CBA, all patients underwent computed tomography (CT) scans to evaluate the anatomy of PVs and left atrium (LA). Three‐dimensional PVs and LA were reconstructed from CT image by Ensite Precision system and the maximal (D_max_) and minimal diameters (D_min_) of all PV ostium were measured. The PV OI was defined as the ratio of D_max_ and D_min_ and the PV OA was calculated as D_max_ × D_min_ × π ÷ 4. During CBA, Time‐to‐isolation (TTI), temperature at 1 minute, and lowest temperature were measured.

**Results:** The mean D_max_ and D_min_ of all PVs were 18.93±4.08 mm and 13.84±3.18 mm. The mean OI of all PVs was 1.41±0.33. The mean OA of all PVs was 210.94±79.92 mm^2^. The mean TTI of all PVs was 35.05±21.94 seconds. The mean lowest temperature of all PVs was ‐53.13±7.36°C. The mean temperature at 1 minute of all PVs was ‐46.14±5.31°C. All PVs were divided into two groups depending on the mean OA of all PVs. During CBA, the group of PV OA ≥ 210mm^2^ had a lower lowest temperature (‐54.35±8.29°C vs. ‐51.6±5.65°C, *p* <0.001) and a lower temperature at 1 minute (‐47.14±5.28°C vs. ‐44.88±5.09°C, *p* <0.001) than the group of PV OA < 210mm^2^. All PVs were divided into two groups according to the mean OI of all PVs. During CBA, the group of PV OI ≥ 1.4 had a longer duration for TTI (37.07±21.23 seconds vs. 32.4±22.62 seconds, *p* = 0.045) than the group of PV OI < 1.4. About different PVs, only in RSPV subgroup, PV OA ≥ 210mm^2^ had a lower lowest temperature (‐56.38±4.83°C vs. ‐54.02±5.1°C, *p* =0.014) than PV OA < 210mm^2^ during CBA.

**Conclusions:** During CBA, PV OA ≥ 210mm^2^ had a lower lowest temperature and a lower temperature at 1 minute than PV OA < 210mm^2^, and PV OI ≥ 1.4 had a longer duration for TTI than PV OI < 1.4.

Chair

**D. E. Chieng**;

St John of God Hospital, Subiaco, Perth, Australia

Chair

**W. W. B. Chik**;

Westmead Hospital, Westmead, New South Wales, Australia

## 17 UTILITY OF ROUTINE SEPTOGRAM TO DELINEATE OCCULT INTERVENTRICULAR SEPTAL PERFORATION AFTER LEFT BUNDLE AREA PACING

### 17.1 **SAROJ KUMAR CHOUDHURY**, DEBABRATA BERA

#### 17.1.1 NH‐RTIICS, KOLKATA, India

**Introduction:** Confirmation of depth of septal lead during left bundle (LB) area pacing (LBAP) is not mandatory and is more of academic interest. We analyzed whether it can detect any occult perforation of left bundle pacing lead among stylet driven lead (SDL) cases of Abbott TENDRIL vs lumen‐less 3830lead (LLL) of Medtronic.

**Methods:** It is a multi‐center observational study where consecutive 40 cases of successful SDL (Abbott) and 22cases of LLL Medtronic leads were included. IVS perforation is suspected while advancement of LB lead when ≥2 of the following 4 was noted: 1. 1. Sudden rise in LB capture threshold. 2. 2. Sudden reduction in current of injury (COI) 3. 3. Reduction of unipolar impedance value drops below 400 ohms or impedance dropped by >100 ohms. 4. 4. Unipolar COI was more in ring than tip electrode. After successful lead placement all cases underwent septogram with gentle injection of 2‐3 ml undiluted contrast.

**Results:** Mean age 64±9 yrs, 35 males. 6 out of 62caseshad evident lead perforation (4/40 SDL and 2/20 LLL) from lead parameters. In addition, 2 more cases(1 male, 1 Female) werenoted where lead parameters were all within acceptable limit but septogram revealed subclinical lead perforation into left ventricle with trickling of contrast into LV and staining of LV endocardium [ Fig 1 and 2]. In all 8 cases, successful lead repositioning was done at alternate site. No major complication observed.

**Conclusions:** Routine Septogram is a procedure without any additional risk to assess the lead depthas well as occult IVS perforation. This shall be routinely performed after successful LBAP implant as 3% cases have occult perforation not detectable by lead parameters. The finding was noted among SDL cases in our cohort.

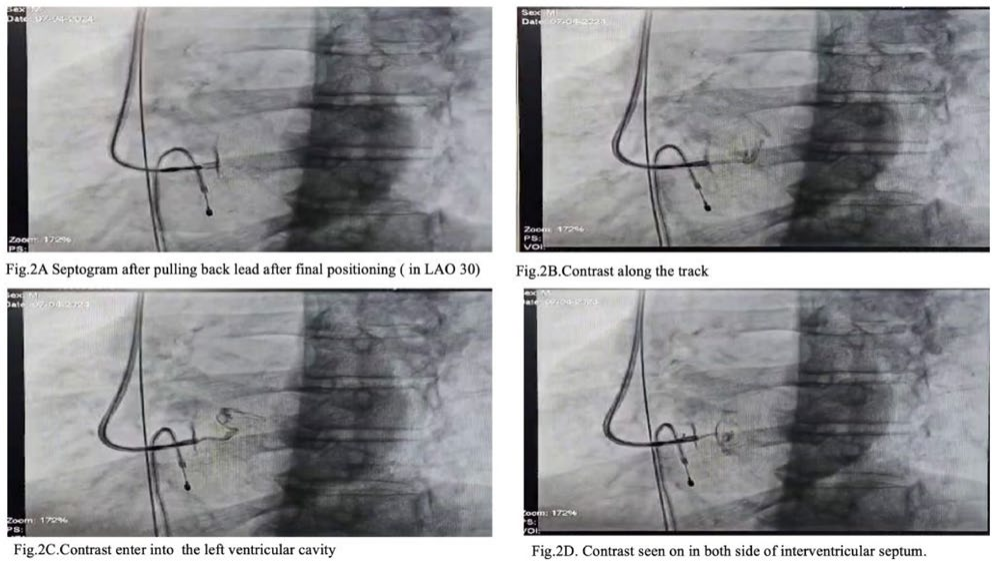

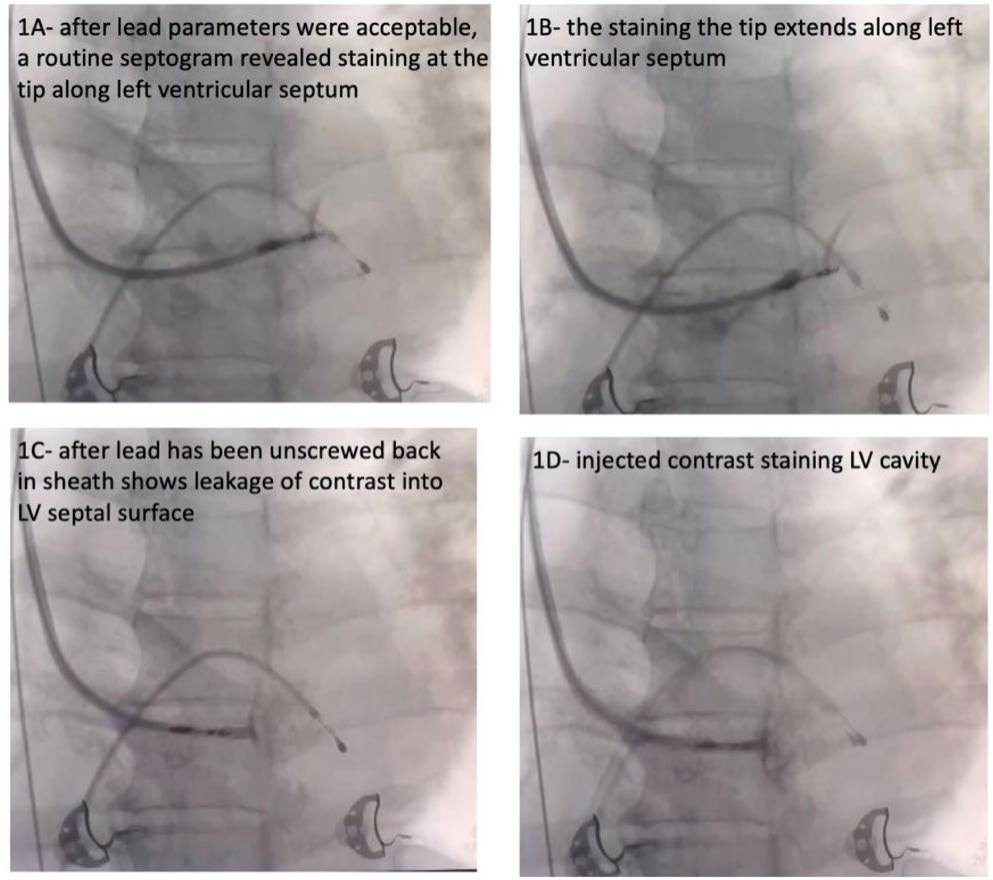

## 18 SUPRAVENTRICULAR TACHYCARDIA ABLATION IN OLDER PATIENTS ‐ CHARACTERISTICS AND OUTCOMES

### 18.1 **YI YI CHUA**, JULIAN TAY, ERIC LIM, XUANMING PUNG, DANIEL CHONG, KAH LENG HO, CHI KEONG CHING

#### 18.1.1 National Heart Centre Singapore, Singapore, Singapore

**Introduction:** Catheter ablation is an effective treatment for symptomatic recurrent supraventricular tachycardia (SVT), a common dysrhythmia that affects patients of all ages. While most studies and guidelines target the general adult population, data older patients are less robust. We studied the differences in clinical and procedural characteristics, and outcomes in older patients (defined by age ≥70 years) undergoing SVT ablation.

**Methods:** All patients of atrioventricular nodal re‐entry tachycardia (AVNRT), atrioventricular re‐entry tachycardia (AVRT), and/or atrial tachycardia (AT) ablation between May 2011 ‐ May 2022 at a tertiary centre were included. Cases with concurrent ablation of atrial flutter, atrial fibrillation and ventricular arrhythmias were excluded. Patients were divided into 2 groups: patients age <70 years and patients ≥70 years.

**Results:** 1758 cases of SVT ablation were included; 1608 were <70 years old, and 150 patients were ≥70 years old. Youngest and oldest patient was aged 8 and 92 respectively. Clinical characteristics differed: older patients were more likely to have underlying structural heart disease and/or ischaemic heart disease, more likely to have AVNRT and less likely to have AVRT (p<0.001). Older patients were more likely to undergo right‐sided ablation and less likely to undergo left‐sided ablation (p<0.001). The use of stereotaxis, intracardiac echocardiography, and electroanatomical mapping did not differ. Procedure time, radiofrequency application time and fluoroscopy time were also significantly shorter in older patients (p<0.05) though fluoroscopy dose area product and total skin dose did not differ. Immediate complication and success rates did not differ.

**Conclusions:** SVT remains an important cause of morbidity amongst older patients. We have shown the acute success rates are high and complication rates are low across all groups despite differences in the clinical and procedural characteristics. SVT ablation should be considered for symptomatic patients regardless of age, though further data including patient comorbidities and longer‐term outcomes may help refine the patient selection process.

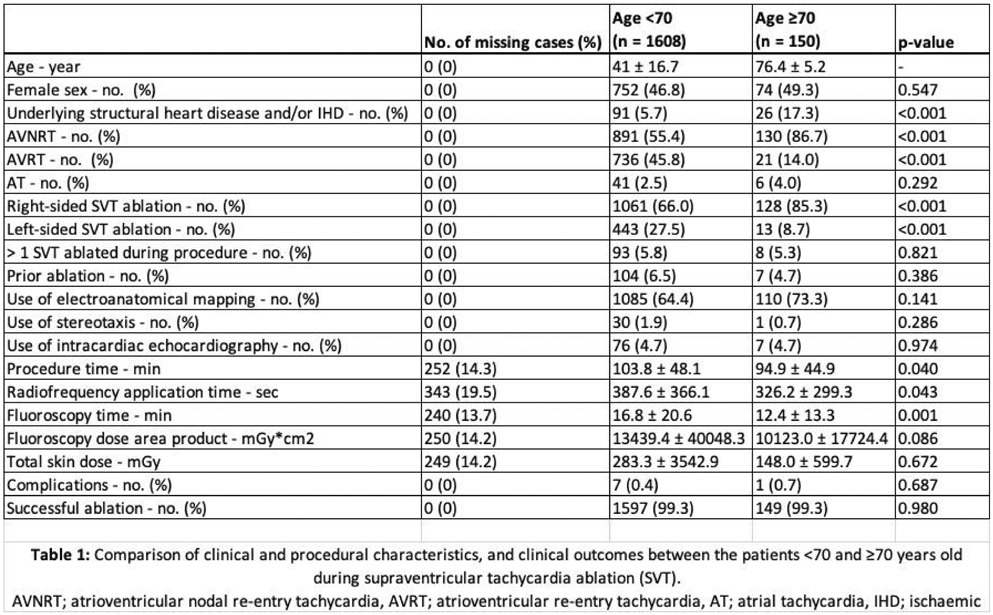

## 19 DIAGNOSIS TO ABLATION TIME IN PERSISTENT AF PATIENTS IN THE CAPLA STUDY

### 19.1 **ROSE CROWLEY** ^1^, MICHAEL LIM^2^, DAVID CHIENG^1^, LOUISE SEGAN^1^, JEREMY WILLIAM^1^, JOSEPH MORTON^2^, GEOFFREY LEE^2^, PAUL SPARKS^2^, ALEX MCLELLAN^2^, HARIHARAN SUGUMAR^1^, SANDEEP PRABHU^1^, LIANG‐HAN LING^1^, ALEKSANDR VOSKOBOINIK^1^, RAJEEV PATHAK^3^, LAURENCE STERNS^4^, MATTHEW GINKS^5^, PRASHANTHAN SANDERS^6^, PETER KISTLER^1^, JONATHAN KALMAN^2^

#### 19.1.1 ^1^Alfred Health, Melbourne, Australia,^2^Royal Melbourne Hospital, Melbourne, Australia,^3^Canberra Heart Rhythm, Canberra, Australia,^4^Royal Jubilee Hospital, Vancouver Island, BC, Canada,^5^John Radcliffe Hospital, Oxford, United Kingdom,^6^Royal Adelaide Hospital, Adelaide, Australia

**Introduction:** Non‐randomised data suggests longer diagnosis to ablation time is associated with poorer outcomes; however, a recent randomised study found no difference in recurrences when ablation was delayed by 12 months. The aim of this analysis was to assess the impact of diagnosis to ablation time on AF recurrence in patients undergoing catheter ablation for persistent AF.

**Methods:** CAPLA was a multicentre trial that randomised patients with PsAF to PVI+ posterior wall isolation or PVI alone. Follow up was 12 months. Outcomes were assessed after a three‐month blanking period.

**Results:** Median DAT in the 334 patients was 28 months (IQR12‐66). Patients were divided into quartile groups. Q1:DAT 0‐12 months (n=84, median DAT 7 months), Q2:13‐28 months (n=85, median DAT 20 months), Q3:29‐66 months (n=84, median DAT 41 months), Q4:DAT ≥67 months (n= 81, median DAT 119 months). Rate of AF recurrence was; Q1:36.9%, Q2:44.7%, Q3:47.6%, Q4:56.8% (p=0.082). On multivariate analysis, DAT Q4 was the only factor significantly associated with risk of recurrence (HR1.607 95%CI 1.005‐2.570 p=0.048). Median AF burden was 0%(0‐0.47) in Q1 and 0.33%(0‐4.6) in Q4 (p=0.002). Quality of life (assessed by AFEQT) improved markedly in all quartiles from baseline to 12 months (Q1:Δ28.8±24, Q2:Δ24.4±23.4, Q3:Δ21.7±26.6, Q4:Δ24.6±21.4,p=0.331).

**Conclusions:** In a cohort of patients with persistent AF undergoing ablation in a prospective trial with standardised entry criteria and intensive ECG monitoring, those with shorter DAT had lower rates of AF recurrence. However, differences were modest and all quartiles demonstrated marked reductions in AF burden and improvements in QoL.

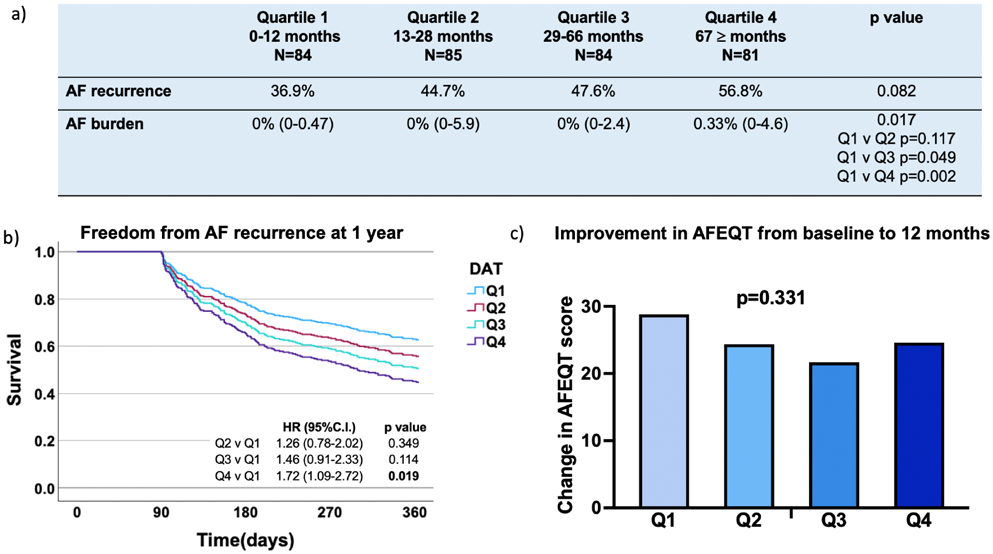

## 20 EFFECT OF PATTERN OF AF AT FIRST DIAGNOSIS ON OUTCOMES OF ABLATION FOR PERSISTENT AF

### 20.1 **ROSE CROWLEY** ^1^, DAVID CHIENG^1^, LOUISE SEGAN^1^, JEREMY WILLIAM^1^, HARIHARAN SUGUMAR^1^, SANDEEP PRABHU^1^, ALEKSANDR VOSKOBOINIK^1^, LIANG‐HAN LING^1^, JOSEPH MORTON^2^, GEOFFREY LEE^2^, PAUL SPARKS^2^, ALEX MCLELLAN^2^, RAJEEV PATHAK^3^, LAURENCE STERNS^4^, MATTHEW GINKS^5^, PRASHANTHAN SANDERS^6^, PETER KISTLER^1^, JONATHAN KALMAN^2^

#### 20.1.1 ^1^Alfred Health, Melbourne, Australia,^2^Royal Melbourne Hospital, Melbourne, Australia,^3^Canberra Heart Rhythm, Canberra, Australia,^4^Royal Jubilee Hospital, Vancouver Island, BC, Canada,^5^John Radcliffe Hospital, Oxford, United Kingdom,^6^Royal Adelaide Hospital, Adelaide, Australia

**Introduction:** Many patients with persistent AF (PsAF) have progressed from initial paroxysmal AF (PAF), however not infrequently patients present with PsAF from the outset. This sub‐analysis of the CAPLA study aimed to assess the impact of pattern of AF at first diagnosis on outcomes of catheter ablation for PsAF.

**Methods:** CAPLA was a multicentre trial that randomised patients with PsAF to pulmonary vein isolation (PVI) plus posterior wall isolation or PVI alone. Patients were followed up for a minimum of 12 months. All outcomes were assessed after a three‐month blanking period.

**Results:** Of the 334 patients (median age 65.6 yrs, 23.1% female), 194(58.1%) had PsAF at time of first diagnosis, and 140(41.9%) had PAF at first diagnosis, in these patients the median time to progression to PsAF was 35.5 months (IQR 14‐94.8). Patients with PsAF from outset were younger (64.0 yrs vs 67.7 yrs p=0.005) and had lower rates of hypertension (40.7% vs 55.7% p=0.007) and ischaemic heart disease (8.8% vs 17.1% p=0.021). They had higher rates of heart failure (51.0% vs 31.4% p<0.001) and heart failure with reduced ejection fraction (34.0% vs 23.6% p=0.039) and lower median LVEF (54.5% vs 60%, p=0.007). AF recurrence occurred in 86(44.1%) patients with PsAF at first AF diagnosis and 70(50%) with PAF at time of diagnosis (p=0.305). PsAF at first diagnosis was not associated with increased risk of AF recurrence on univariate (HR 0.802 95% CI 0.585‐1.101, p=0.173) or multivariate analysis (HR 0.921 95%CI 0.647‐1.312, p=0.650). There was no significant difference in baseline left atrial size (PAF at outset: 46.3+/‐13 ml/m^2^ vs PsAF at outset: 48.4+/‐15.9 ml/m^2^ p=0.337) or amount of left atrial low voltage area(PAF at outset: 31.8%+/‐17.3 vs PsAF at outset: 32.4%+/‐12.6 p=0.530).

**Conclusions:** Pattern of AF at first diagnosis did not impact rate of AF recurrence in patients with PsAF who underwent catheter ablation. Patients with PsAF at first AF diagnosis were younger, with higher rates of heart failure compared to those with initial PAF that progressed to PsAF.

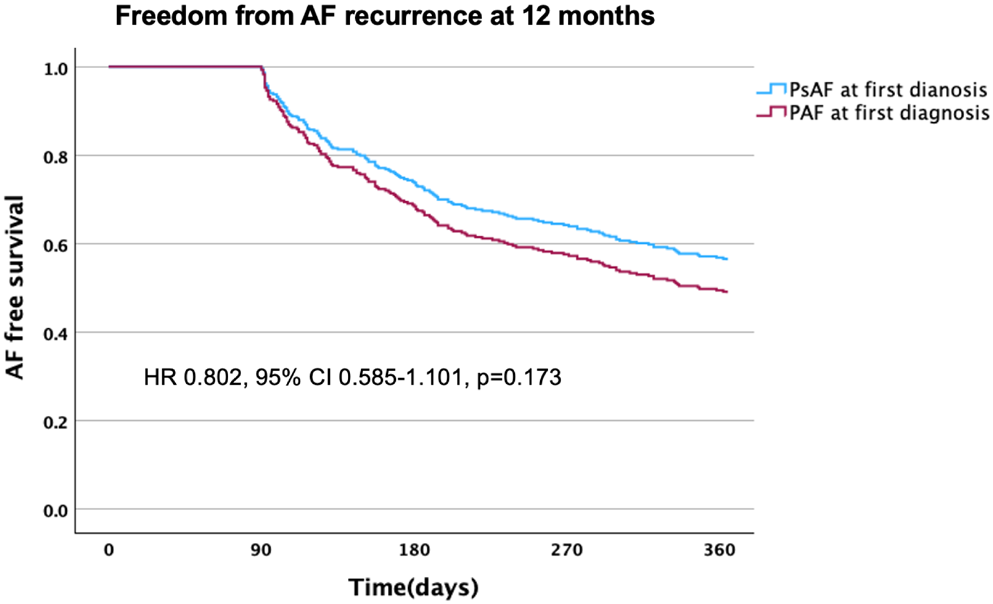

## 21 ARRHYTHMIAS & REMODELLING IN LIFELONG & RETIRED MASTER ATHLETES VS CONTROLS

### 21.1 **PAOLO D'AMBROSIO** ^1,2,3^, JARNE DE PAEPE^4^, KRISTEL JANSSENS^1,5^, AMY MITCHELL^1^, JOSHUA MCDONALD^1^, TIM VAN PUYVELDE^4^, STEPHANIE ROWE^1,6^, OSCAR CULLEN^1^, LUKE SPENCER^1^, SOFIE VAN SOEST^4^, RIK WILLEMS^4^, HEIN HEIDBUCHEL^7^, PETER KISTLER^3,8,9^, GUIDO CLAESSEN^10^, JONATHAN KALMAN^2,3^, ANDRE LA GERCHE^1,6,11^

#### 21.1.1 ^1^St Vincent's Institute, Melbourne, Australia,^2^Department of Cardiology, Royal Melbourne Hospital, Melbourne, Australia,^3^Department of Medicine, University of Melbourne, Melbourne, Australia,^4^Department of Cardiovascular Diseases, University Hospitals Leuven, Leuven, Belgium,^5^The Mary MacKillop Institute for Health Research, Melbourne, Australia,^6^Department of Cardiology, St Vincent's Hospital, Melbourne, Australia,^7^Department of Cardiology, University Hospital Antwerp, Antwerp, Belgium,^8^Department of Cardiology, The Alfred Hospital, Melbourne, Australia,^9^Department of Medicine, Monash University, Melbourne, Australia,^10^Department of Cardiology, Hartcentrum, Jessa Ziekenhuis, Hasselt, Belgium,^11^HEART Lab, Victor Change Cardiovascular Research Institute, Sydney, Australia

**Introduction:** Endurance athletes are predisposed to atrial arrhythmias (AAs) but the association between intensive endurance exercise & ventricular arrhythmias (VAs) is less well established. We aimed to define the prevalence of AAs and VAs in master endurance athletes vs matched controls and assess associations between arrhythmias, fitness and measures of cardiac remodelling.

**Methods:** Holter monitors in 185 athletes and 81 controls were analysed for the presence of AAs and VAs. Athletes were categorised as active lifelong (n=144, ≥5hr/wk & ≥120%) vs retired (n=41, <5hr/wk & <120%) based on amount of high intensity exercise ≤ 5 years of enrolment and % of predicted VO_2_ Max. Exercise volume during active years (MET hrs/wk) was quantified by multiplying MET‐equivalents for each sport & intensity by hrs/wk. Cardiopulmonary exercise testing, echocardiography and contrast CMR were performed. Those < 40 yrs and with known heart disease were excluded.

**Results:** Athletes (57yr, 83% male) were significantly fitter (VO_2_ Max: 41 vs 35 ml/kg/min, *p*<0.001) and exercised for longer (36 vs 13 yrs, *p*<0.001) at higher volumes (MET hrs/wk: 96 vs 18, *p*<0.001) vs controls (53yr, 96% male). Athletes had significantly more non‐sustained ventricular tachycardia (NSVT: 15% vs 1%, *p*<0.001) and sustained AAs (17% vs 0%, *p*<0.001) with AF accounting for 87%. Neither group had sustained VAs. Athletes had larger RVEDV*i* (114 vs 100ml/m^2^, *p*<0.001) with lower RVEF (48% vs 53%, *p*<0.001), larger LAV*i* (46 vs 32ml/m^2^, *p*<0.001) and more hinge (27% vs 9%, *p*<0.001) and non‐hinge late gadolinium enhancement (LGE; 28% vs 9%, *p*<0.001). Despite evidence of reverse remodelling (Figure 1), there was no difference in prevalence of NSVT, sustained AAs or non‐hinge LGE between lifelong and retired athletes.

**Conclusions:** Master athletes have a significantly higher prevalence of NSVT and sustained AAs vs matched controls. In older athletes, the prevalence of arrhythmias is similar regardless of whether they are actively engaged in training or retired suggesting that pro‐arrhythmic remodelling in athletes is sustained, not immediately reversible and may be minimally responsive to detraining.

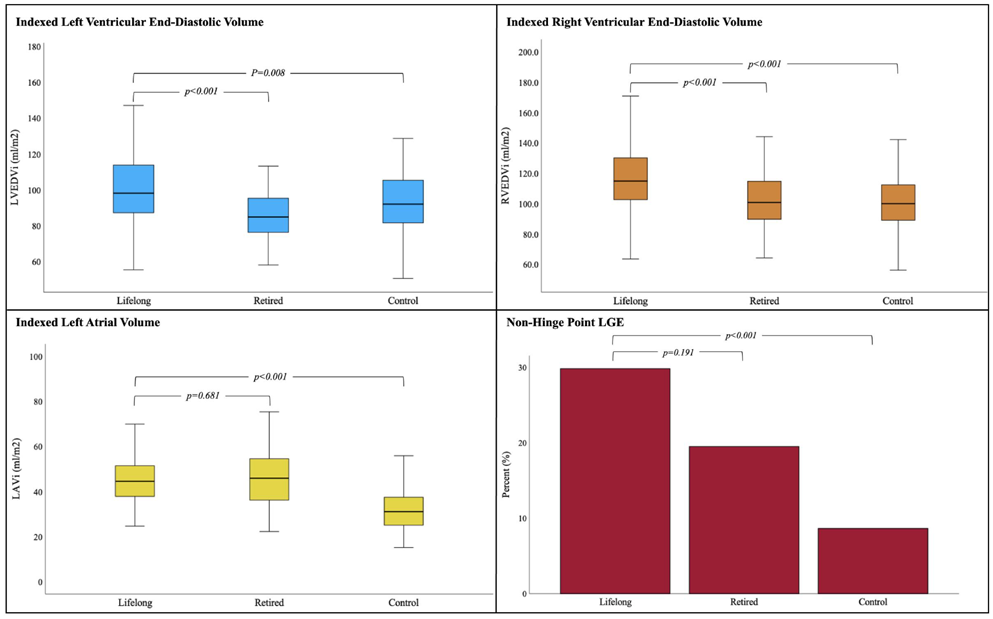

## 22 PERCLOSE PROSTYLE VASCULAR CLOSURE DEVICE FOR EXPEDITED AMBULATION FOLLOWING ATRIAL FIBRILLATION ABLATION UTILISING 16.8 FRENCH DIAMETER SHEATH

### 22.1 **ALEXANDER DASHWOOD** ^1^, STEWERT HEALY^1^, ROBERT PUCHALSKI^2^, SING HUEY CHENG^1^, BRENDAN TIAN^3^, EMILY KOTSCHET^1^

#### 22.1.1 ^1^Victorian Heart Hospital, Melbourne, Australia,^2^The Cardiac Centre, Gold Coast, Australia,^3^Monash University, Melbourne, Australia

**Introduction:** Atrial fibrillation (AF) ablation often involves large‐diameter catheters, posing challenges for post‐procedure hemostasis and discharge. The impact of Perclose Prostyle™ closure devices on time to ambulation is uncertain.

**Methods:** 50 patients undergoing AF ablation with 16.8 French sheaths were randomly assigned in a 1:1 ratio to Prostyle or standard closure with a figure‐of‐eight suture. A maximum of three Prostyles were allowed. Pre‐closure was achieved with deployment at 2 and 10 o’clock positions and a final Prostyle secured the second 7 French short sheath. Baseline data and post‐procedure ambulation times were collected. Follow up was two weeks.

**Results:** Ultrasound access was utilised for all 50 participants, mean age 62 ± 12 years. No significant differences were noted in age, BMI, CHADsVASC score, time since last anticoagulation or final activated clotting time between the two groups. Mean time to ambulation was significantly shorter in the Prostyle group compared to the standard of care (217 ± 120 vs. 320 ± 70 minutes; p = 0.0018). All Prostyles for the 16.8 sheaf closures were deployed successfully. Two Prostyles for the 7 French sheaths were unsuccessful. Three Prostyle patients required additional manual pressure or FemoStop. The standard of care group required three FemoStops and one patient developed a large hematoma. No serious adverse events were reported.

**Conclusions:** In this small, randomised control trial Prostyle utilisation shortened ambulation time post‐AF procedures with large catheters, suggesting benefits for recovery and same‐day discharge.

Chair

**D. Davis**;

University of Ottawa, Heart Institute, ON, Canada

## 23 SUTURE SLEEVE IMPROVEMENTS TO HOLD FORCE AND LEAD CRUSH RESISTANCE IMPROVES OVERALL SYSTEM NOISE PERFORMANCE

### 23.1 WESLEY ALLEMAN, STEVE CHANTASIRIVISAL, **MATT DESMOND**, KEITH VICTORINE

#### 23.1.1 Abbott, Sylmar, CA

**Introduction:** Suture sleeves secure implanted transvenous leads at the venous entry sites. Low holding force between the sleeve and lead body can cause the need for overtightening when securing the sleeve, and low resistance to crush can damage both the sleeve and lead body. Abbott SurGrip™ suture sleeves are designed to improve performance, yielding improved reliability.

**Methods:** Engineering assessed the hold force between the sleeve and the lead, and protection of the lead body against overtightening, comparing SurGrip technology vs. the predicate Tendril™ STS 2088TC suture sleeve. A surgeon knot using a non‐resorbable braided size 0 suture was used, with forces of 0.9‐3.6 kg and soaked in water prior to testing. Holding force test quantitatively determined the force required to slip the tied sleeve from the lead body. Lead body crush testing quantitatively determined the compressive force applied to the lead body during tie‐down. Following implementation of the new sleeve, a complaint comparison was performed on 86,000 leads with a mix of SurGrip suture sleeves and predicate sleeves implanted over a two year timeframe.

**Results:** The hold force showed improvement on 2‐groove tie down forces. At 1.8 kg of tie‐down force, the hold force of the SurGrip sleeve is 2x greater than the predicate sleeve (Figure 1A). For crush resistance, the SurGrip sleeve had a 1.6x improvement at 3.6 kg tie‐down vs. the predicate sleeve (Figure 1B). X‐ray scans confirm the crush resistance provided by the SurGrip sleeve vs. the predicate sleeve (Figure 1C). Product performance analysis after two years show a 46% reduction in system noise and a 55% reduction in general sleeve complaints, when assessing sleeves of both designs implanted since 2022.

**Conclusions:** Bench testing demonstrates improvements in key performance metrics of the SurGrip suture sleeve compared to the predicate design. SurGrip sleeves provide higher holding force with less tie‐down effort, while providing greater crush resistance at higher tie‐down forces. A review of real‐world data shows a reduction of associated complaint rates relative to the predicate devices within a two year timeframe.

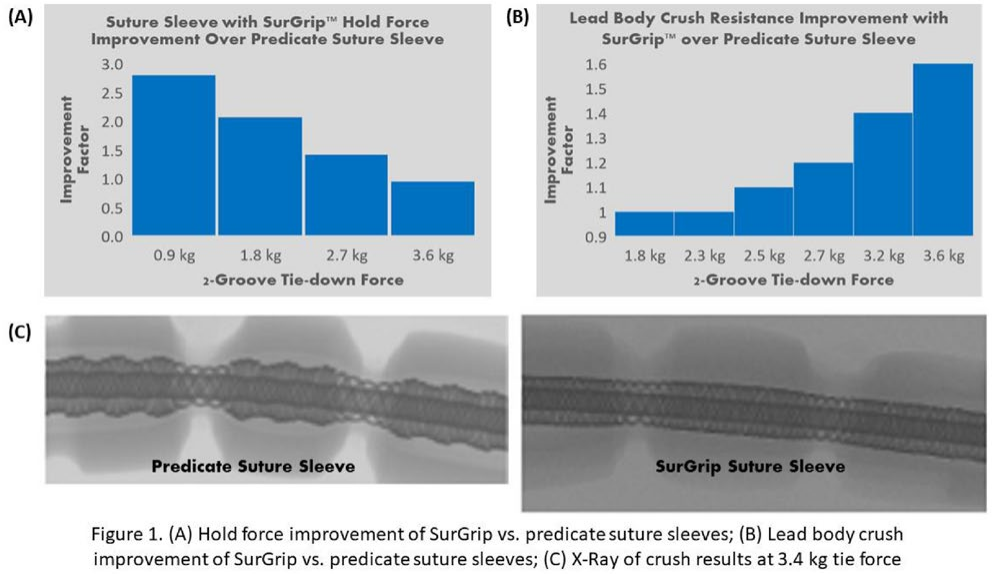

## 24 CATHETER ABLATION FOR PEDIATRIC PATIENTS WITH ARRHYTHMIA INDUCED CARDIOMYOPATHY

### 24.1 **YUJI DOI** ^1^, ANDREW DAVIS^1,2,3^, MELLISA TIEN^2^, ANDREAS PFLAUMER^1,2,3^

#### 24.1.1 ^1^The Royal Children's Hospital, Melbourne, Australia,^2^University of Melbourne, Melbourne, Australia,^3^Murdoch Children's Research Institute, Melbourne, Australia

**Introduction:** Arrhythmia induced cardiomyopathy (AICM) is potentially reversible once the responsible arrhythmia is controlled, though genetic cardiomyopathy might play a role. While ablation can be a cure, risk is higher in newborns or those on mechanical circulatory support (MCS). We sought to identify the patient characteristics and outcome of pediatric patients who underwent ablation for AICM.

**Methods:** A single center retrospective study including patients who had ablation for presumed AICM after 2011. Patient characteristics, types of arrhythmias, ablation and latest follow up data were obtained.

**Results:** There were 19 cases (10 female) in total with the following distribution: Atrial ectopic tachycardia (AET) = 7, junctional reciprocating tachycardia (PJRT) = 6, ventricular tachycardia (VT) = 2, frequent premature ventricular contraction (PVC) = 1, atrial flutter (AFL) = 1. All patients had initial left ventricular ejection fraction (LVEF) of less than 50 % and all but two had left ventricular end‐diastolic dimension (LVEDD) Z score above 2. Median age at presentation was 6.5 (0 ‐ 14.6) years and mean follow up was 5.1 (0.6 ‐ 12.4) years. Body weight at initial ablation was 20.5 (3.2 ‐ 71) kg, four ablations were performed under MCS. 10/19 (53%) had more than a month period of medical management prior, with only three having normalization of LVEF/LVEDD by the time of ablation. 16/19 (84%) had successful ablation resulting in recovery of function and discontinuation of antiarrhythmic medication. 2/19 (11%) needed a second ablation. Of note, six cases were previously considered for heart transplant with five of them successfully recovering after ablation and one requiring heart transplant eventually due to underlying gene positive cardiomyopathy. All three unsuccessful cases were managed medically. One newborn with PJRT had AV block due to ablation, no other significant complications were noted.

**Conclusions:** Ablation for pediatric patients in AICM has a high long term success rate with an acceptable complication rate even for patients of a severity that potentially require heart transplant. Genetic Cardiomyopathies can present with arrhythmia as secondary symptom.

## 25 HOPEFUL INSIGHTS ON IMPLANTABLE CARDIOVERTER DEFIBRILLATOR IN ATHLETES WITH CONGENITAL OR ACQUIRED ARRHYTHMIA CHOOSING TO RETURN‐TO‐PLAY: A META‐ANALYSIS

### 25.1 **RIFQI RIZKANI ERI** ^1^, SANIA ZAHRANI^2^, PRASETYO ANDRIONO^1^

#### 25.1.1 ^1^Abdi Waluyo Hospital, Central Jakarta, Indonesia,^2^University of Indonesia, Faculty of Medicine, Central Jakarta, Indonesia

**Introduction:** Arrhythmia poses a significant risk to athletes due to the threat of sudden cardiac death during physical exertion. Current guidelines restrict athletes with arrhythmias and history of cardiac events from participating in most competitive sports. Implantable cardioverter defibrillators (ICDs), however, provide a career‐saving treatment option, with latest studies suggesting its safety and efficacy, offering hope for these athletes to continue pursuing their dreams.

**Methods:** A thorough search across PubMed, PMC, SCOPUS, and Embase was done using the keywords 'athlete' and 'implantable cardioverter defibrillator'. Studies were included if they examined the efficacy and safety of ICDs in athletes with arrhythmias returning to play, diagnosed arrhythmias using clinical features and/or gene screening, involved competitive athletes, and used syncope, cardiac arrest, lethal arrhythmias, and sudden cardiac death as cardiac events. Exclusion criteria incluides studies involving non‐competitive athletes, insufficient data for prognostic analysis, or non‐English publications. The Newcastle‐Ottawa scale was used to assess study quality, done by two independent reviewers with a third resolving conflicts.

**Results:** Five high‐quality cohorts with a total of 1031 participants were included for the final review, with a mean follow‐up duration of 30 to 85 months. Our meta‐analysis revealed encouraging findings: shock‐related physical injury and cardiac events were both reported at 0%. We also found the following rates: 1% for quitting sports due to shocks, 4% for inappropriate shocks during exertion, and 12% for appropriate shocks during exertion.

**Conclusions:** Our meta‐analysis provides encouraging insights for athletes with arrhythmias considering a return‐to‐play with ICDs. ICDs have a promising role in enabling athletes with arrhythmias to safely return to competitive sports. Future research should focus on long‐term outcomes and the optimization of guidelines for athlete eligibility with ICDs in sports settings.

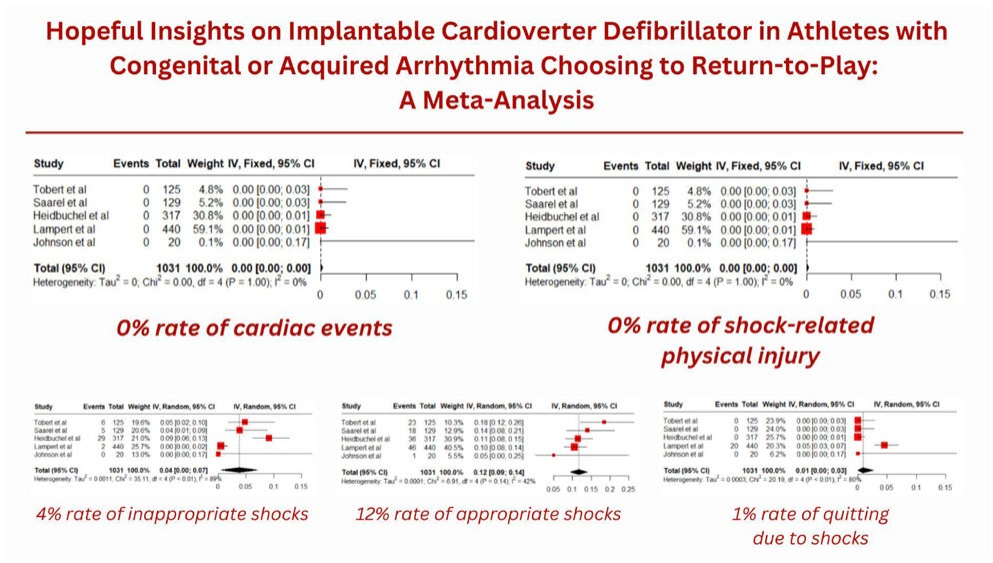

## 26 OPTIMAL AF DETECTION DURATION FOR LOOP RECORDERS: A DUAL‐LEVEL ANALYSIS

### 26.1 **SHAUN EVANS** ^1^, SURAYA HANI KAMSANI^1,2^, JOHN FITZGERALD^1^, MOHANARAJ JAYAKUMAR^1^, MOHAMED ABBAS^1^, ELNAZ SHAHMOHAMADI^1^, MELISSA MIDDELDORP^1,3^, PRASHANTHAN SANDERS^1^

#### 26.1.1 ^1^Centre for Heart Rhythm Disorders, University of Adelaide, Adelaide, Australia,^2^National Heart Institute, Kuala Lumpur, Malaysia,^3^Department of Cardiology, University of Groningen, University Medical Center Groningen, Groningen, Netherlands

**Introduction:** Implantable loop recorders (ILR) play a major role in the diagnosis of atrial fibrillation (AF). However, continuous monitoring has the potential to produce false positives which contribute to alert fatigue and significant workforce burden. Sensitivity and specificity for AF detection vary between devices and algorithms. The optimal AF duration threshold to reduce false positive episodes is unknown.

**Methods:** This study aims to determine the optimal duration threshold for AF detection with ILR. AF alert transmissions from LINQII (Medtronic) device between January 2022 and March 2023 were randomly selected. After adjudication by trained physicians, the alerts were classified as true or false positive events and the duration of each event was recorded. The sensitivity and specificity of AF detection was calculated for varying duration thresholds in a dual‐level analysis: The sensitivity was reported on a per‐patient basis, reflecting the overall diagnostic accuracy. The specificity was reported on a per‐alert basis to capture the workload associated with the burden of individual alert review.

**Results:** A total of 197 AF episodes were evaluated. Median duration of the episodes was 8 mins (IQR:2‐52mins). Twenty‐eight percent of the episodes were false positive alerts (n=55). An alert threshold duration of 15 minutes provided a per‐alert specificity of 0.80, and a patient‐level sensitivity of 0.88. The median latency to diagnosis introduced by the 15‐minute threshold was 0 days.

**Conclusions:** Raising the AF duration alert threshold for Medtronic LINQII devices to 15 minutes may provide acceptable sensitivity to detect AF episode while rejecting up to 80% of false positive AF alerts. This does not appear to introduce any significant delay to AF diagnosis.

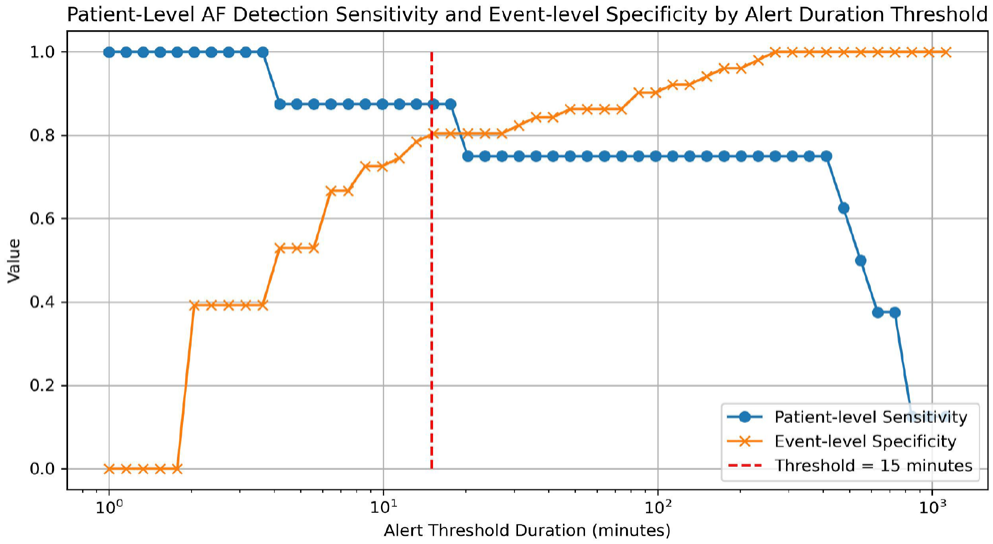

## 27 NON‐FLUOROSCOPIC RADIOFREQUENCY CATHETER ABLATION OF RIGHT‐SIDED ACCESSORY PATHWAYS IN CHILDREN USING 3D MAPPING SYSTEM

### 27.1 **WEN‐PO FAN** ^1^, CHIEH‐MAO CHUANG^2^, PI‐CHANG LEE^2^, YU‐CHENG HSIEH^2^, CHENG‐HUNG LI^2^, YUN‐CHING FU^2^, YENN‐JIANG LIN^1^, SHIH‐LIN CHANG^1^, LI‐WEI LO^1^, YU‐FENG HU^1^, FA‐PO CHUNG^1^, CHIN‐YU LIN^1^, TING‐YUNG CHANG^1^, LING KUO^1^, CHIH‐MIN LIU^1^, SHIN‐HUEI LIU^1^, CHENG‐I WU^1^, GUAN‐YI LI^1^, YU‐SHAN HUANG^1^, MUHAMMAD RAFDI AMADIS^1^, BAI SITTI AMEERAH ASLEAH B TAGO^1^, MARIE KIRK PATRICH MARAMARA^1^, CHIAO‐CHIN LEE^1^, LO‐CHIEH LING^1^, SHIH‐ANN CHEN^2^

#### 27.1.1 ^1^Taipei Veterans General Hospital, Taipei, Taiwan,^2^Taichung Veterans General Hospital, Taichung, Taiwan

**Introduction:** Non‐fluoroscopic radiofrequency (RF) catheter ablation of arrhythmias benefits patients, especially children, by eliminating radiation exposure. The non‐fluoroscopic ablation of left‐sided accessory pathway (AP) has been proved to be safe and effective.

**Methods:** The electrophysiologic study and transcatheter ablation were performed under fluoroscopic guidance, 3D‐EAM guidance, or both for patients with right‐sided APs. In the 3D‐EAM guidance group, the 3D reconstruction of right atrium, right ventricle and coronary sinus was completed in every procedure. A 5F or 7F 4‐mm non‐irrigated ablation catheter were mostly used, inserted into right femoral vein, and mapped the tricuspid annulus. RF ablation was performed at the site with the earliest antegrade ventricular and/or retrograde atrial activation.

**Results:** From March 2012 to May 2023, we included 66 patients (mean age: 11.5 years, mean body weight: 43.6 kg) with manifest or concealed right‐sided APs underwent transcatheter RF ablation in three hospitals. Among the patients, 30 received non fluoroscopic procedure. There were no significant differences in acute success rate, procedural time, AP locations, number of RF application, time to AP block, and minor complications between fluoroscopic group (X+) and non‐fluoroscopic group (X‐). No major complication was noted in both groups. No significant differences in recurrence‐free survival between the two groups (p=0.137) (Figure 1A). Mechanical trauma of APs during catheter manipulation was highly related to recurrence, irrelevant to guiding systems (Figure 1B).

**Conclusions:** The non‐fluoroscopic transcatheter RF ablation of right sided APs in pediatric patients is feasible and safe compared with fluoroscopic transcatheter RF ablation.

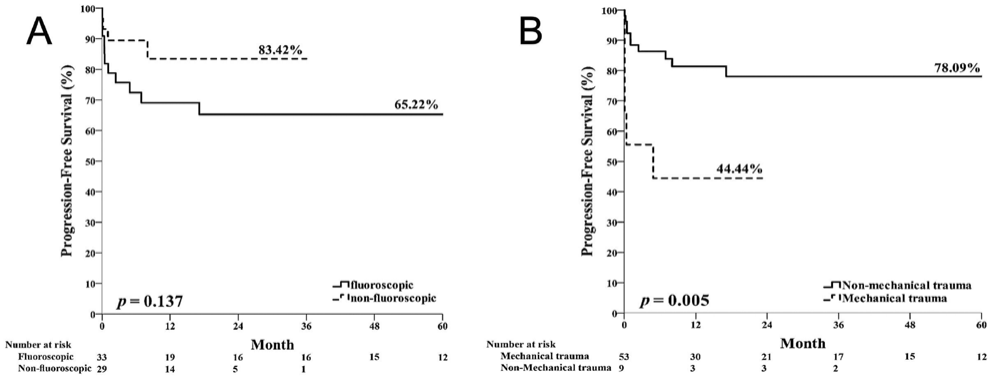

## 28 THE IN‐VITRO EFFECTS OF LOW‐ENERGY VERSUS HIGH‐ENERGY PHOTON BEAM RADIATION ON CARDIAC IMPLANTABLE ELECTRONIC DEVICES

### 28.1 **MEGAN FRASER**, MARK LOWREY, NICK WEST, KADHIM KADHIM, EWEN SHEPHERD

#### 28.1.1 Newcastle upon Tyne Hospitals Trust, Newcastle upon Tyne, United Kingdom

**Introduction:** Radiotherapy treatment has been shown to cause malfunctioning of cardiac implantable electronic devices (CIED). The current guidelines for radiotherapy patients with CIED advise limiting cumulative dose to 5Gy, using beam energies <10MV, and relocating the CIED if device exposure is unavoidable. This advice can negatively impact optimal radiotherapy treatment and increase the patient's risk of infection by relocating the CIED. There is an increased risk using beam energies >10MV as they produce secondary neutrons which are known to affect the electronics of the CIED leading to device malfunction. This study evaluated two treatment groups, low‐energy flattening filter (6MV) and high‐energy flattening filter‐free (10FFF) photon beam radiation to assess device malfunction at the same 48Gy cumulative dose.

**Methods:** Two treatment groups irradiated ninety‐four pacemakers and defibrillators with a cumulative dose of 48Gy. The CIEDs were programmed VVI/DDD at maximum sensing sensitivity and placed directly in the centre of the treatment field. 6MV group were irradiated in eight fractions at 6Gy/min, and 10FFF irradiated in a single fraction at 24Gy/min.

**Results:** One malfunction occurred in the 6MV group vs. eleven in the 10FFF group (*p* = 0.003). The 10FFF group experienced one inappropriate shock and two device reset failures. The only malfunction in the 6MV group was pacing inhibition showing malfunction severity increases at higher dose rates and beam energy. Newer generation models produced fewer malfunctions than legacy devices (5% vs 17.5%, *p* = 0.003). Newer CIED models are known to have protective software capabilities to reduce CIED malfunction.

**Conclusions:** Radiotherapy dose rate and beam energy have a greater effect on CIED malfunction than total cumulative dose. Reprogramming CIED to asynchronous pacing with ICD therapies off could reduce malfunctions and should be attempted before relocation of CIED is considered. Newer generation CIED may help prevent malfunction; therefore, current guidelines may be outdated as they are based on research performed on legacy devices which will soon become irrelevant.

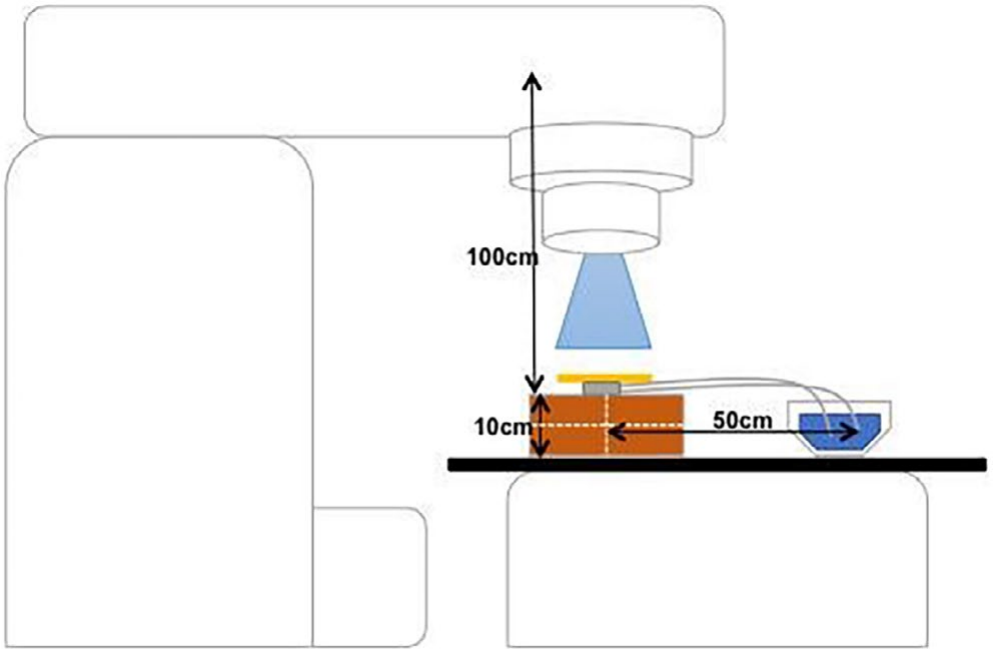

## 29 THE PROGNOSTIC BENEFIT OF CONDUCTION SYSTEM PACING OVER RIGHT VENTRICULAR PACING IN PATIENTS WITH ATRIAL FIBRILLATION: A RETROSPECTIVE COHORT STUDY

### 29.1 **BINGQI FU**, WEI HUA

#### 29.1.1 Fuwai Hospital, Beijing, China

**Introduction:** Few studies have focused on the clinical application of conduction system pacing (CSP) in patients with atrial fibrillation (AF) requires ventricular pacing. This study aims to evaluate whether CSP, as compared to right ventricular pacing (RVP), results in prognostic benefit among patients with AF.

**Methods:** This study was conducted between September 2018 and September 2022, and included patients with AF and LVEF ≥ 40% that required ventricular pacing. The primary outcome was all‐cause mortality, and the secondary outcome was heart‐failure rehospitalization (HFH).

**Results:** Eventually, 301 patients were included and were divided into CSP (n=98) and RVP (n=203). During a mean follow‐up of 21.4 months, CSP demonstrated a higher ventricular pacing percentage (CSP: 86.1% vs. RVP: 29.6%, p < 0.001) and a narrower paced QRS duration (CSP: 119.9 ± 20.5 ms vs. RVP: 153.0 ± 27.6 ms, p < 0.001) compared to RVP. Additionally, CSP exhibited a significantly lower rate of all‐cause mortality (Log‐rank = 8.8, P = 0.003; **Figure 1A**) and HFH (Log‐rank = 4.4, P = 0.03; **Figure 1B**). Univariate Cox regression analysis indicated a reduced risk of all‐cause mortality (HR = 0.14, 95% CI 0.03‐0.59, P = 0.007) and HFH (HR = 0.40, 95% CI 0.17‐0.96, P = 0.040) in CSP . This association remained significant after adjusting for confounders, showing an independent reduction in the risk of all‐cause mortality (HR = 0.15, 95% CI 0.03‐0.68, P = 0.014) and HFH (HR = 0.37, 95% CI 0.16‐0.87, P = 0.022) in CSP. In the subgroup analysis of patients with paroxysmal AF, CSP was linked to a lower risk of both all‐cause mortality (HR = 0.11, 95% CI 0.02‐0.78, P = 0.027) and HFH (HR = 0.11, 95% CI 0.02‐0.61, P=0.012). Among patients without previous radiofrequency catheter ablation, CSP showed a reduced risk of HFH (HR = 0.35, 95% CI 0.14‐0.86, P = 0.022).

**Conclusions:** In patients with AF required ventricular pacing, CSP demonstrated significantly lower risk of all‐cause mortality and HFH compared to RVP, suggesting a prognostic benefit of CSP over RVP.

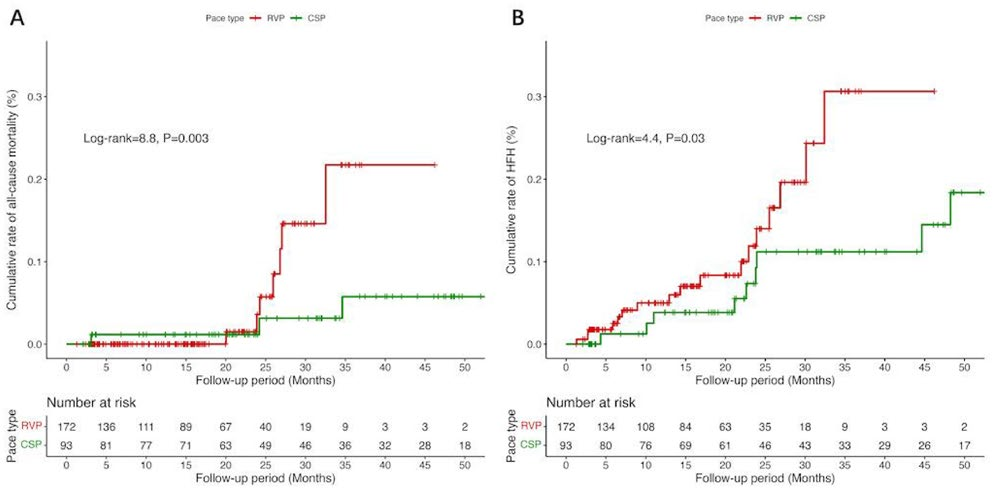

## 30 COST‐EFFECTIVENESS OF INSERTABLE CARDIAC MONITOR IN UNEXPLAINED SYNCOPE IN CHINA

### 30.1 YIJIA TANG^1^, BAOJIAN ZUO^2^, BINBIN CHEN^2^, CHANGSHENG FAN^2^, JIE ZHOU^1^, YIN SONG^3^, **JIN FU** ^3^

#### 30.1.1 ^1^Sichuan Provincial People's Hospital, Chengdu, China,^2^Beijing Medical and Health Economic Research Association, Beijing, China,^3^Medtronic Greater China, Shanghai, China

**Introduction:** Insertable cardiac monitor (ICM) is recommended to help establish a diagnosis in patients with unexplained syncope. Compared with conventional testing (CONV), ICM offers continuous long‐term ECG monitoring and has superior ability to establish symptom‐rhythm correlation, which is crucial for patients with infrequent and random syncope. In China, ICMs trigger relatively higher upfront costs. However, the consequent costs and outcomes of additional diagnosis and resulting treatments remain indistinct. We seek to evaluate the economic value of ICM compared with CONV for the diagnosis of arrhythmia in unexplained syncope patients from the perspective of China's healthcare system, with a view of informing clinical and policy decisions.

**Methods:** We used a decision tree‐Markov model to assess the lifetime costs and benefits of arrhythmia diagnosis with ICM versus CONV. In the model, all related diagnostic and follow‐up arrhythmia‐related treatment costs and consequences were considered. The cohort characteristics and costs were derived from a retrospective real‐world study conducted in Sichuan Provincial People's hospital. Risks of mortality, syncopal recurrence, syncope‐related injury, and quality of life were obtained from the literature. For each strategy, the total costs and quality adjusted life‐years (QALYs) were modelled, and the incremental cost‐effectiveness ratio (ICER) was calculated. Three times China's GDP per capita ($36,930.2) was used as the willingness‐to‐pay (WTP) threshold.

**Results:** ICM was more costly but more effective than CONV. The total discounted costs and QALYs of ICM strategy were $14,604.7 and 9.1 QALYs, whereas the CONV strategy was associated with the cost of $11,493 and 8.9 QALYs. The ICM strategy contributed to the overall 0.2 QALYs gained, and $3,111.6 costs increased. The ICER was $17,780.8/QALY, around 1.4 times China's GDP per capita, much lower than the WTP threshold. Therefore, ICM strategy is a cost‐effective strategy compared to CONV in patients with unexplained syncope.

**Conclusions:** ICM strategy is more cost‐effective than CONV in establishing a diagnosis in recurrent unexplained syncope in China.

## 31 THE FIRST REAL‐WORLD STUDY OF FARAPULSE PULSED FIELD ABLATION SYSTEM IN A CHINESE POPULATION WITH PAROXYSMAL ATRIAL FIBRILLATION

### 31.1 **YANG GANG** ^1,2^, WEIZHU JU^2^, SHUYING QI^3^, ZHI YU^3^, HAIXIONG WANG^4^, XIANHUI ZHOU^5^, MINGLONG CHEN^6^

#### 31.1.1 ^1^Jiangsu Province Hospital Chongqing Hospital, Nanjing, China,^2^Jiangsu Province Hospital, Nanjing, China,^3^Boao Super Hospital, Qionghai, China,^4^Shanxi Cardiovascular Hospital, Taiyu, China,^5^The First Affiliated Hospital of Xinjiang Medical University, Urumqi, China,^6^Jiangsu Province Hospital Hospital, Nanjing, China

**Introduction:** Pulsed field ablation (PFA), an emerging atrial fibrillation (AF) ablation energy with the potential to optimize ablation outcomes, has accumulated numerous clinical data, while these trials were mainly performed in European or US centers. This study aimed to present the initial results of FARAPULSE PFA system in a Chinese population.

**Methods:** Benefiting from Priority Use policy in China, paroxysmal AF patients were prospectively enrolled in BoAo Super Hospital in Hainan China and accepted pulmonary vein isolation (PVI) with FARAPULSE PFA system. Follow‐up would be ECG and 24h Holter at 6 and 12 months, any rhythm monitoring performed under standard of care would be collected too. The efficacy endpoints included acute PVI rate and atrial arrhythmia recurrence rate at 12 months. The safety endpoint was the incidence of major complications during the study.

**Results:** Thirty subjects were enrolled and all the procedures were performed under general anaesthesia by 14 new PFA operators from 11 hospitals in 10 provinces. 30% cases underwent high‐density mapping. The procedural time and left atrial dwell time were 77.8 ± 22.9 mins and 37.9 ± 12.8 mins. Acute PVI rate was 100% with no major complications. Three minor vessel access complications happened without intervention or prolonged hospitalization. The mapping of electrical PV antrum coverage suggested operators should pay attention to catheter manipulation to achieve good adhesion to the anterior of right pulmonary veins and the crista in the left side in initial cases. All the subjects except one accepted ECG and 24h Holter at 3‐,6‐ and 12‐month visits. The proportion of subjects free from atrial arrhythmia recurrence was 76.5% at 12 months. Two of the three re‐ablation cases showed PVI durability. Four of seven recurrence subjects had early recurrence during blanking period too.

**Conclusions:** This first PFA real‐world study performed in Chinese AF patients showed satisfied safety and efficacy results even in the scenario of all the procedures completed by new PFA operators.

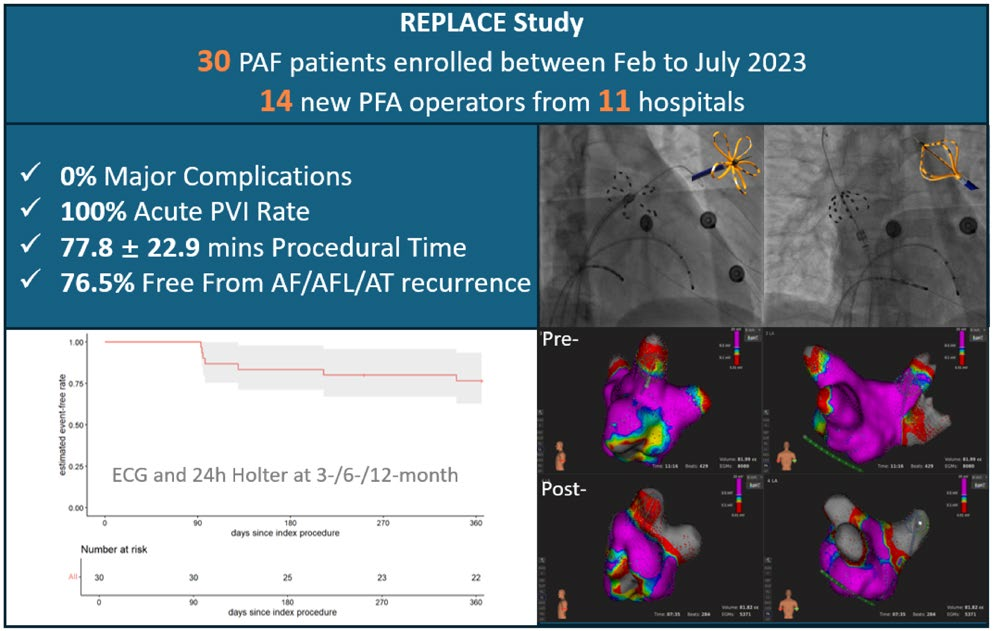

Chair

**M. Gawalko**;

Medical University of Warsaw, Warsaw, Poland

## 32 A COMPLEX WOLFF‐PARKINSON‐WHITE (WPW) SYNDROME CASE ‐ ABLATION OF ACCESSORY PATHWAY WITH MULTIPLE EXTENSIONS

### 32.1 **MAHENDRA GUNASEKARA**, ASUNGA DUNUWILLE

#### 32.1.1 Institute of Cardiology, Colombo, Sri Lanka

**Introduction:** Managing Wolff‐Parkinson‐White (WPW) syndrome with multiple accessory pathway extensions presents significant challenges in electrophysiological mapping and ablation. This report explores treatment complexities.

**Methods:** N/A

**Results:** A 31‐year‐old woman presented with frequent palpitations requiring multiple hospital admissions. Resting ECG showed sinus rhythm, WPW pattern, and a negative delta wave in lead II, suggesting a right‐sided accessory pathway in the coronary sinus; 2DE is normal. An EP study via the right femoral vein identified a septal pathway. Initial attempts with Decapolar and Quad catheters induced AVRT. Mapping revealed retrograde VA conduction at CS 3,4 with partial placement of the Decapolar catheter due to a tight tricuspid valve. This favored a septal pathway with a TCL of 308 ms. The ablation catheter initially mapped the right postero‐septal area without optimal signals. Retrograde aortic insertion from the right femoral artery revealed early fused AV signals near CS 3,4. Radiofrequency ablation (RFA) transiently terminated tachycardia, but preexcitation persisted. Reintroducing the Decapolar catheter via the right internal jugular vein improved maneuverability but did not achieve perfect signals. Subsequent use of an irrigated‐tip ablation catheter inside CS 3,4 showed excellent pre‐delta wave mapping preceding the delta wave by 32 ms. RF pulses successfully separated fused signals.Re‐stimulation induced tachycardia recurrence with altered CS activation. Mapping via the retrograde aortic route from the left side achieved an activation map of 54 ms, eliminating early fused signals within 8 seconds. Concentric CS activations with separated AV signals ensued. The 3‐hour procedure included 12 minutes of RFA at 40 W / 60°C and a 3‐minute cooled‐tip ablation. WPW syndrome with posteroseptal accessory pathways, right, and left extensions, was confirmed and successfully ablated.

**Conclusions:** This case shows meticulous mapping and ablation's adaptability in managing complex WPW syndrome. Successful ablation alleviated symptoms, normalizing the post‐procedure ECG.

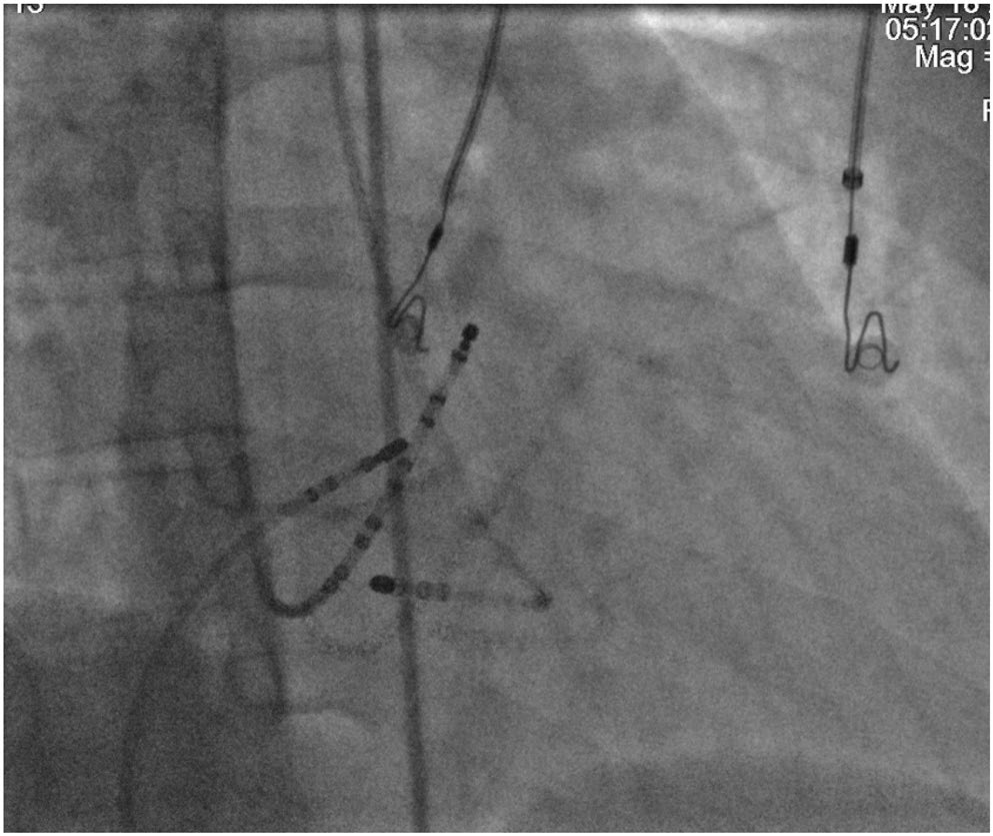

## 33 CARDIAC RESYNCHRONIZATION THERAPY WITH LEFT‐SIDE PERSISTENT SUPERIOR VENA CAVA BY USING LIMITED AND UNCONVENTIONAL HARDWARE

### 33.1 **MAHENDRA GUNASEKARA**, ROHAN GUNAWARDENA, NUWAN RATHNAYAKE, NILANTHA PERAMUNA ARACHCHIGE, CHASIKA WEERTASEKARA

#### 33.1.1 Institute of Cardiology, National Hospital of Sri Lanka, Colombo, Sri Lanka

**Introduction:** Cardiac resynchronization therapy (CRT) is effective for heart failure and electrical dyssynchrony, but anatomical variations like persistent left superior vena cava (PLSVC) complicate the procedure. PLSVC, found in 0.3‐0.5% of the general population, presents challenges for CRT‐P implantation, especially with LV lead placement. Economic constraints often make passive fixation LV leads a challenging yet economical choice.

**Methods:** A 65‐year‐old male with hypertension, dyslipidemia, and a smoking history presented with NYHA Class IV dyspnea and fatigue despite optimal medical therapy. ECG showed left bundle branch block (QRS > 160 ms), and echocardiography revealed a 20% ejection fraction with myocardial dyssynchrony. The patient met ESC guidelines for CRT‐P. Left cubital venograms revealed PLSVC draining into a dilated coronary sinus (CS) with no apparent tributaries. Right side venogram confirmed Type 3B PLSVC. An initial attempt at CS cannulation with a Decapolar EP catheter was unsuccessful. A 6F AL2 guiding catheter and Terumo 0.035 guidewire entered the CS main trunk but failed to identify a suitable CS tributary. A 5F 'Bern' catheter, a specific catheter used in interventional radiology, facilitated targeting the postero‐lateral branch. Using an extended PCI guidewire, stable LV lead placement was achieved. Subsequent axillary vein punctures allowed active fixation of RV and RA leads. Good parameters were achieved, and the post‐op chest X‐ray was normal. The patient's symptoms improved remarkably.

**Results:** N/A

**Conclusions:** Cardiac resynchronization therapy in patients with PLSVC presents significant procedural challenges. Through careful planning, utilization of advanced tools, and procedural adjustments, successful outcomes can be achieved.

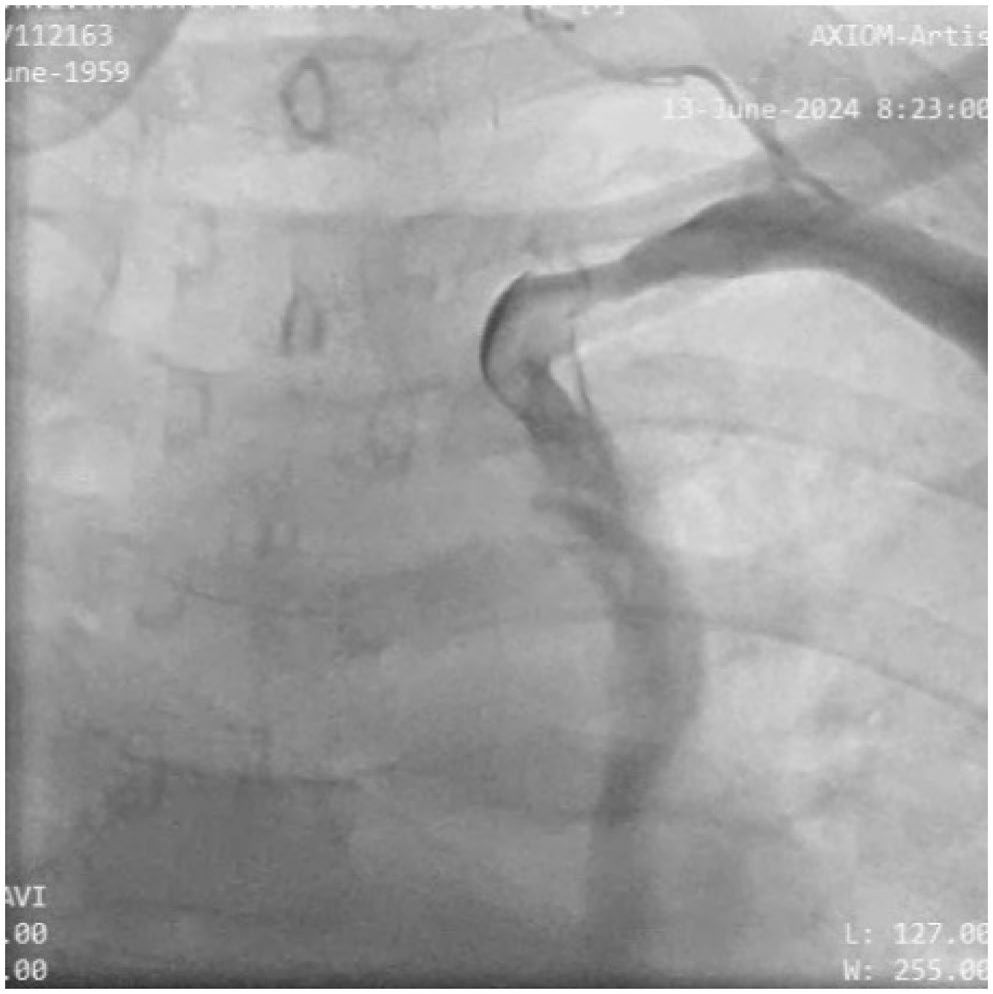

## 34 OPTIMIZING CIED ACCESS¦ SRI LANKA’S CARDIAC CARE AUDIT

### 34.1 **MAHENDRA GUNASEKARA**, ASUNGA DUNUWILLE, ROHAN GUNAWARDENA, SAMEERA ELAPATHA

#### 34.1.1 Institute of Cardiology, Colombo, Sri Lanka

**Introduction:** Cardiac rhythm management (CRM) treats arrhythmias with catheter ablation and cardiac implantable electronic devices (CIEDs), such as implantable cardioverter‐defibrillators (ICDs), pacemakers, and cardiac resynchronization therapy (CRT). The National Hospital of Sri Lanka's Cardiac Electrophysiology Department performs over 600 CIED implantations annually, the most in the country. A clinical audit found factors affecting patient flow, leading to several changes to ensure fair access to CIED procedures, including a real‐time system to monitor CIED availability and manage patient details for pending procedures, known as the Device Availability Surveillance & Patient Allocation Management System (Figure 1).

**Methods:** A retrospective clinical audit was conducted at the department analyzing 117 patients who had CIED procedures from September to November 2023. Data from hospital records were analyzed to evaluate hospital stay length, factors causing delays, preoperative patient traits, and the link between complications and stay duration. Statistical analyses employed parametric and nonparametric tests, with p‐values under 0.05 deemed significant.

**Results:** During this period, 76 males and 41 females underwent CIED implantation. The average hospital stay was 4.05 days, and 86% of procedures were completed in a single admission. Delays and extended stays resulted from poor patient flow management, unawareness of CIED availability at scheduling, and pre‐op issues like uncontrolled blood sugar, anticoagulation mismanagement, or complications like hematoma, pneumothorax, pericardial effusion, or device‐related infections.

**Conclusions:** The study found several correctable causes of delays in CIED implantation. Priorities included enhancing pre‐admission patient education, prioritizing procedures based on urgency, and reorganizing device storage and accessibility. An online system was developed to track devices, monitor available stock, allocate devices to patients, and schedule procedures. These changes have already reduced congestion and allowed for timelier procedures. Future audits will evaluate their impact and ensure care quality.

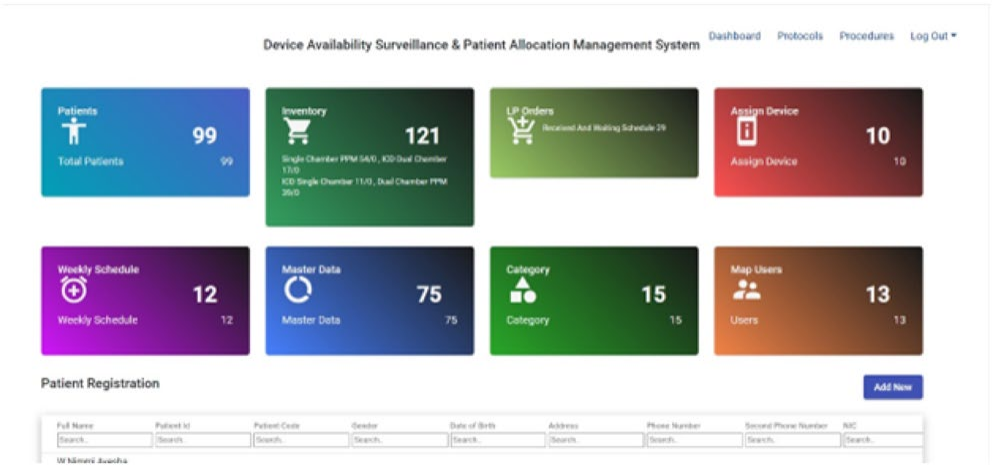

## 35 BRAIN PERFUSION IN PATIENTS WITH ATRIAL FIBRILLATION AND ASSOCIATION WITH COGNITIVE FUNCTION. A PILOT STUDY

### 35.1 JANA HASKOVA

#### 35.1.1 IKEM, Institute for Clinical and Experimental Medicine, Prague, Czech Republic

**Introduction:** Hemodynamic changes associated with atrial fibrillation (AF) may decrease brain perfusion. This may result in cognitive dysfunction. Goal:To evaluate acute and long‐term effect of AF on brain perfusion and cognitive function.

**Methods:** Patients planned for electrical cardioversion of persistent AF underwent brain magnetic resonance imaging (MRI) before and 3 months later. After early phase, subsequent patients had brain MRI also the same day after cardioversion and cognitive tests were performed before and 3 months after. All patients signed their informed consent. Brain perfusion was measured using a 3T MR scanner with native arterial spin labelling method (ASL). We also performed 3D T2 FLAIR and DWI sequences and structural images by 3D MP‐RAGE sequence. During pre‐processing, perfusion maps were co‐registered with structural images. Finally, paired t‐test was performed on normalized perfusion maps with a statistic threshold of p = 0.001. Cognitive examination consisted of a battery of tests, including Montreal cognitive test, psychomotoric tempo, Executive function (Prague Stroop test) and Philadelphia memory test.

**Results:** In early phase, 10 patients (6 males; age: 70.3±3.8 y) had MRI only prior to ECV and 6.8±1.3 weeks later. Five patients remained in sinus rhythm and had significant rise of perfusion, predominantly in the frontal and occipital regions. Subsequent 9 patients (7 males; age 65.7±9.1y) had MRI also just after cardioversion. All but three had sinus rhythm and documented increase in perfusion. Five patients were studied 3 months later with additional improvement in brain perfusion (Figure). Cognitive tests before cardioversion shoved deficit in MOCA and memory tests (Z‐score ‐ 0.752 and ‐0.585 against normal population) with significant improvement 3 months later in sinus rhythm (Z‐score +0.86 and +0.396, respectively) with improved psychomotoric tempo.

**Conclusions:** Our pilot data suggest that rhythm control strategy may improve cerebral perfusion in patients with AF immediately after cardioversion and 3 months later. Maintenance of sinus rhythm appears to be associated with improved cognitive function.

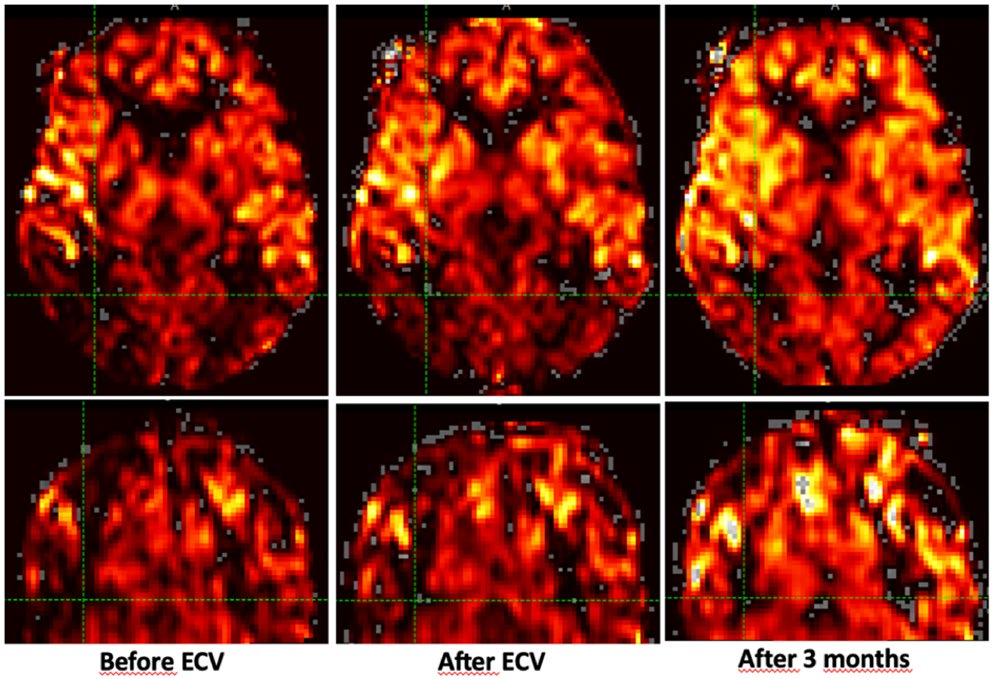

## 36 THE TYPE OF RECURRENT ATRIAL TACHYCARDIA AFTER THE EXTENSIVE LINEAR ABLATION FOR PERSISTENT ATRIAL FIBRILLATION USING CRYOBALLOON FOR LEFT ATRIAL ROOF AND CHEMICAL ABLATION FOR MITRAL ISTHMUS

### 36.1 **KAZUTO HAYASAKA** ^1^, YASUHIRO SHIRAI^2^, TAKESHI SASAKI^1^, AYAKA USUI^1^, RYOTARO FUNAYAMA^1^, JUNICHI DOI^1^, SHU YAMASHITA^1^, SHINSUKE MIYAZAKI^3^, TETSUO SASANO^3^

#### 36.1.1 ^1^NHO Disaster Medical Center, Tachikawa, Tokyo, Japan,^2^AOI Universal Hospital, Kawasaki, Kanagawa, Japan,^3^Tokyo Medical and Dental University, Bunkyo, Tokyo, Japan

**Introduction:** The efficacy of linear ablation for atrial fibrillation (AF) has been reported to be limited. We previously reported the extensive linear ablation strategy for persistent AF that contains multiple energy sources such as radiofrequency, cryoballoon for pulmonary vein isolation (PVI) and left atrial roof ablation (LAR‐ABL), and ethanol ablation for Marshall vein (EI‐VOM) in mitral isthmus ablation (MI‐ABL) to improve the lesion durability. However, the pattern of atrial tachycardia (AT) recurrence after the strategy remains unclear.

**Methods:** Among 243 persistent AF patients who had undergone the extensive ablation between May 2018 and January 2022, recurrent 11 AT patients who had received a redo procedure were analyzed. ATs were classified according to the activation mapping with CARTO 3 system module and a multielectrode mapping catheter, and the diagnosis of circuit was confirmed by entrainment pacing.

**Results:** The main structures involving recurrent AT were cavo tricuspid isthmus (CTI) (N=1), PV (N=2), left atrial wall (N=1) and perimitral isthmus (N=7) whereas LAR was not included in the recurrent AT circuit. Regarding reconnection of the previous ablated block line, reconnection was observed in CTI (N=1, 9%), PVs (N=2, 18%), LAR (N=2, 18%) and perimitral isthmus (N=8, 72.7%).

**Conclusions:** After the extensive ablation strategy for persistent AF that contains cryoballoon for PVI and LAR‐ABL, and EI‐VOM in MI‐ABL, multiple ATs occurring using epicardial connections in addition to PM‐AT, which necessitates detailed mapping and selection of the optimal ablation sites.

## 37 NOVEL EXPANDABLE CRYOBALLOON ABLATION FOR ATRILA FIBRILLATION : MULTICENTER EXPERIENCE OVER 500 CASES

### 37.1 YOSUKE HAYASHI

#### 37.1.1 Sakakibara Heart Institution, Tokyo, Japan

**Introduction:** Novel expandable diameter cryoballoon ablation has been available. However, the post‐procedural clinical outcomes remained unclear. We evaluated the short‐term treatment outcomes at 6 month after cryoballoon ablation.

**Methods:** This multi‐center observational study included 535 consecutive atrial fibrillation patients (mean age 67.4 ± 11.1, men in 70%, paroxysal in 65%) who underwent pulmonary vein (PV) isolation using expandable diameter cryoballoons capable of ablation at 28‐mm or 31‐mm.

**Results:** Out of 2,006 targeted PVs, 1,950 (97.2%) were successfully isolated by a sized‐adjustable cryoballoon, while the remaining 56 required touch‐up ablation. Among them, 1,219 (62.5%) PVs were isolated by a 31‐mm balloon. The biophysical parameters, time‐to‐isolation and the rate of complication were similar regardless of experience of operators or the timing of availability. Out of 535 patients, 174 completed 6‐month follow‐up. After a mean follow‐up of 7.0 ± 2.0 months, 85 patients (89%) were free from atrial fibrillation recurrence and 66 patients (80%) with non‐PAF were free (log‐rank test P = 0.043). In multivariate Cox model paroxysmal atrial fibrillation was the only predictor for freedom of the recurrence [HR = 0.35 (0.15 ‐ 0.85), P = 0.020]. Phrenic nerve injury occurred in 36 patients (6.3%).

**Conclusions:** Short‐term treatment efficacy and safety after the novel expandable cryoballoon ablation was feasible.

## 38 IMPACT OF REDO ATRIAL FIBRILLATION ABLATION

### 38.1 **CHARLES YAO‐CHENG HO** ^1^, ANDREW MARTIN^1^, NIGEL LEVER^1,2^, MATTHEW O'CONNOR^1^, ANDREW GAVIN^3^, FANG SHAWN FOO^3^, JAMIE VOSS^4^, KHANG LI LOOI^1^

#### 38.1.1 ^1^Te Whatu Ora ‐ Auckland Hospital, Auckland, New Zealand,^2^University of Auckland, Auckland, New Zealand,^3^Te Whatu Ora ‐ Waitemata, Auckland, New Zealand,^4^Te Whatu Ora ‐ Counties Manukau, Auckland, New Zealand

**Introduction:** Atrial fibrillation (AF) recurrences after pulmonary vein (PV) isolation (PVI) are mainly due to PV reconnections however durable PVI is also observed. Optimal redo strategy other than PV reisolation is unknown. This study analyses the clinical characteristics and impact of redo AF ablation strategies in a single tertiary centre (Auckland, New Zealand).

**Methods:** Patients ≥18 years underwent redo AF ablations between 2020 ‐ 2023 were included. Clinical background, procedural details, and atrial arrhythmia recurrence were obtained from procedural database and compared.

**Results:** 133 patients (61±11 years, 82.0% male, 63.9% European, 46.6% paroxysmal AF) underwent redo ablations for AF recurrences. Mean CHA_2_DS_2_VASc score 1.0±1.1. Structurally normal heart comprised 65%. During redo procedure, 81 (60.9%) patients had reconnected PVs and 52 (39.1%) had durable PVIs. Table 1 summarises redo ablation strategies. After 13.7±3.0 months follow up, 21 (23.3%) patients had atrial arrhythmia recurrence (median 7.8 months, 95%CI 5.0 ‐ 10.6). Anterior mitral line (HR 6.28, 95% CI 1.11 ‐ 35.5, p=0.04) and electrogram (EGM) based ablation (HR 2.55, 95% CI 1.01 ‐ 6.43, p=0.05) were associated with increased arrhythmia recurrence. Posterior wall isolation trended towards arrhythmia free survival (HR 0.3, 95%CI 0.07 ‐ 1.20, p=0.08). Those with durable PVI were less likely to undergo PV based strategy while other strategies were similar. No significant differences in arrhythmia free survival (Diagram 1).

**Conclusions:** In redo AF ablations, anterior mitral line and EGM based ablations appears to be associated with increased atrial arrhythmia recurrence while posterior wall isolation demonstrated a trend towards reduction. Longer follow‐up and a larger study are needed to ascertain significance. Finding of durable PVI did not alter non‐PV based ablation strategy and arrhythmia free survival was similar.

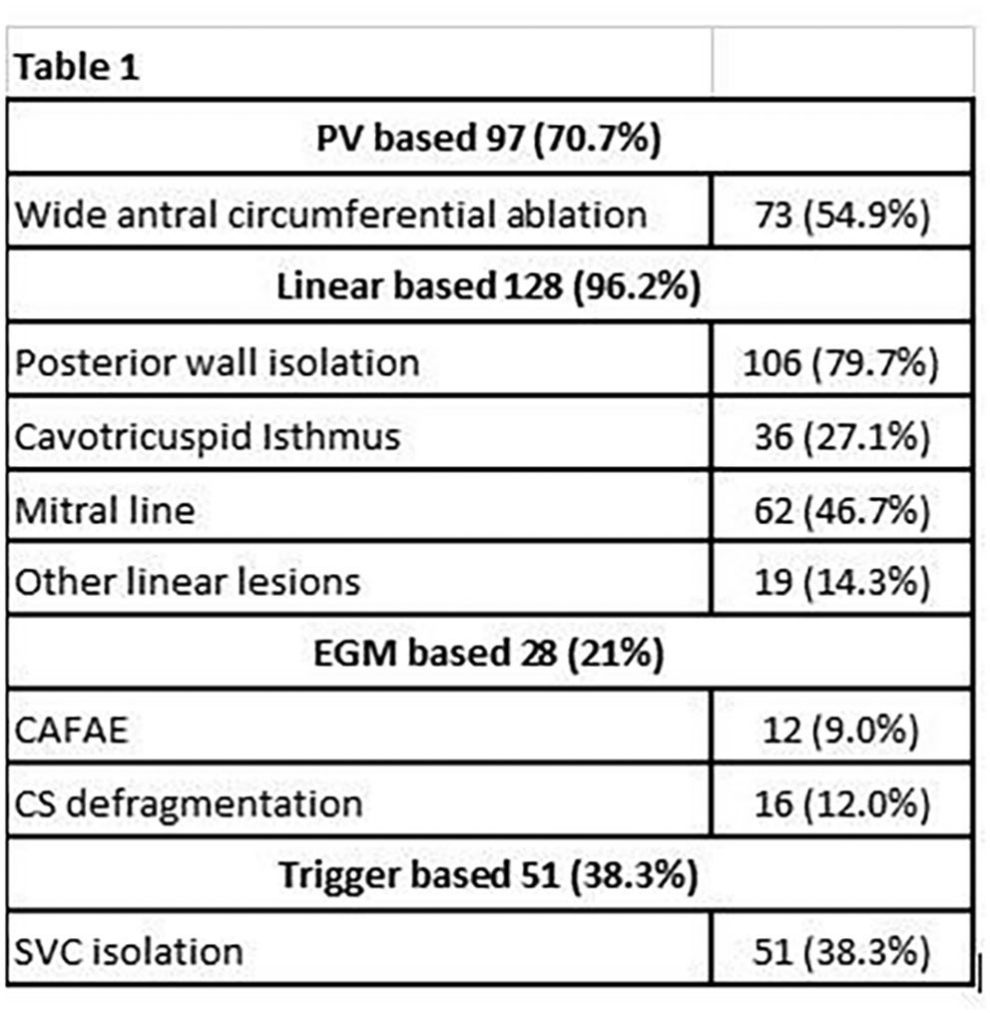

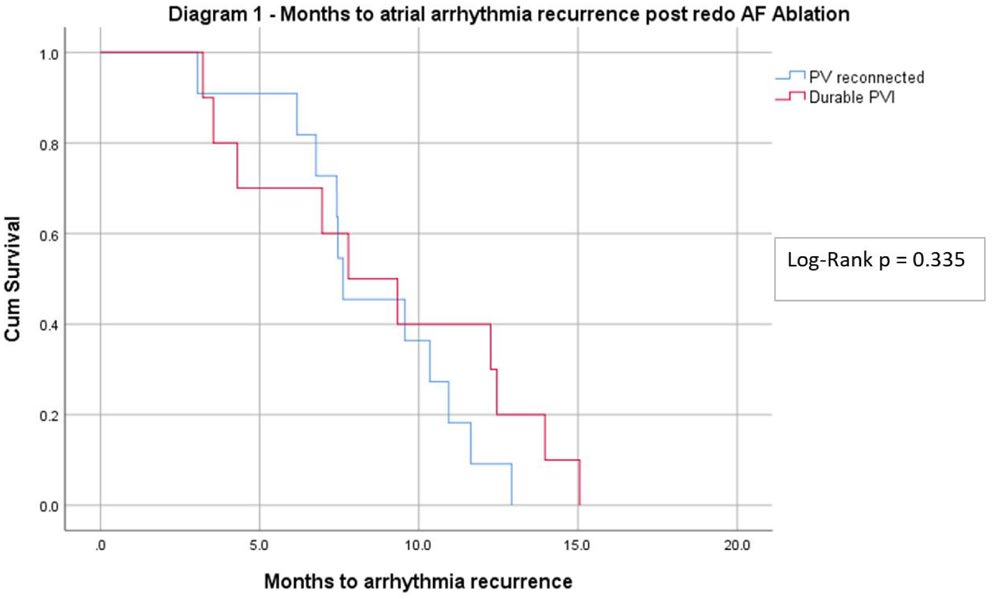

## 39 SAFETY OF PORTABLE SMART HEART RHYTHM DEVICES IN CHILDREN WITH CARDIOVASCULAR IMPLANTABLE ELECTRONIC DEVICES

### 39.1 **CHUN LOK HO**, SIT YEE KWOK, SABRINA SIU LING TSAO, YIU FAI CHEUNG

#### 39.1.1 Hong Kong Children's Hospital, Hong Kong, Hong Kong

**Introduction:** Portable smart heart rhythm devices can provide on‐demand recording of electrocardiogram (ECG) and have been shown to allow better symptom‐rhythm correlation. However, their clinical utility in children implanted with CIEDs is yet to be studied. We aimed to evaluate the safety of using these devices in children with CIEDs by monitoring for electromagnetic interference (EMI) and assessing their ability to generate useful ECGs.

**Methods:** In this prospective cross‐sectional study, patients aged 5 years and older with CIEDs were recruited during their clinic follow‐up at a tertiary children's hospital in Hong Kong. For each subject, a total of four 30‐second ECGs were acquired using KardiaMobile 6L (KM) and Apple Watch (AW). During ECG acquisition, subjects were monitored for any new onset of cardiac symptoms, and CIEDs were assessed for any evidence of EMI. The acquired ECGs were reviewed for the accuracy of heart rate measurements, interpretability, detection of non‐capture during pacing threshold tests, and agreement of ECG parameters with the corresponding standard 12‐lead ECGs.

**Results:** Thirty‐two patients, with a median age of 13 (range 5 ‐ 22), were studied. No adverse clinical events were identified. The entire cohort showed no EMI during or after ECG acquisition in both devices, irrespective of patient and CIED characteristics. Both devices demonstrated excellent accuracy in measuring heart rate (ρ = 0.99) and good agreement in measured ECG parameters (ρ =0.6 ‐ 0.8). 98% acquired ECGs were interpretable, and both devices were 90% sensitive in identifying non‐capture events.

**Conclusions:** Both Kardiamobile and Apple Watch are safe and generate clinically useful ECGs in pediatric patients with CIEDs. Portable smart heart rhythm devices hold promise for effective rhythm monitoring in this population.

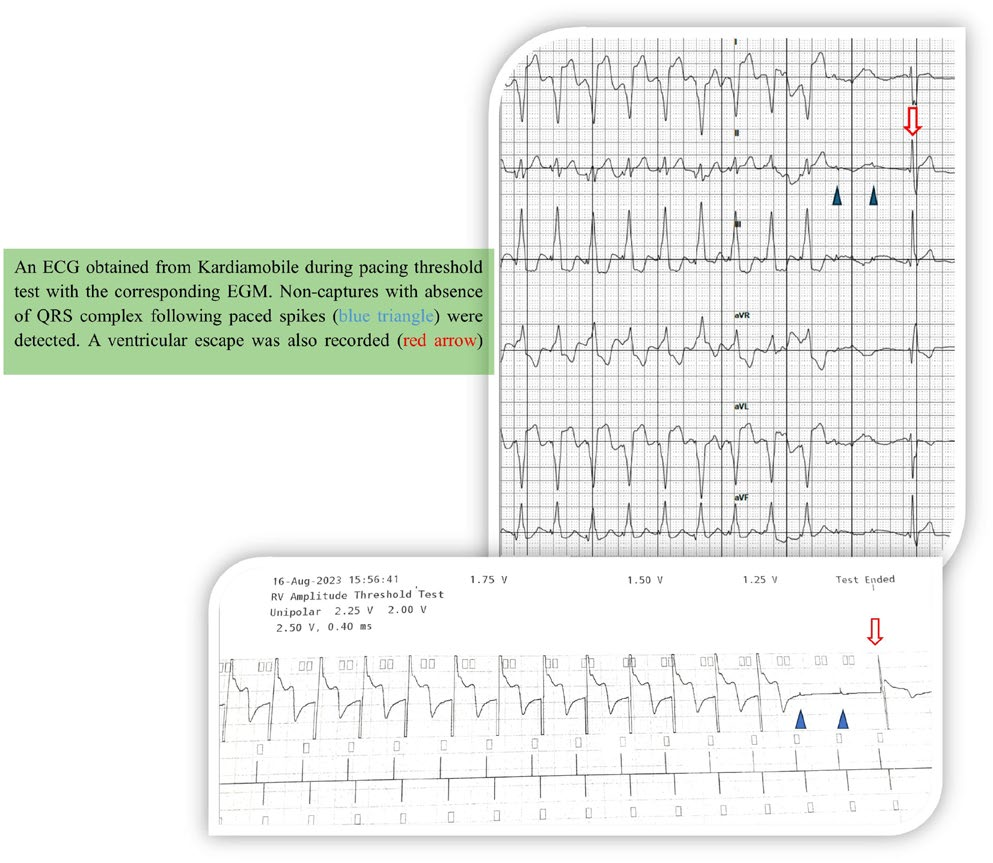

## 40 DIFFERENTIAL PREDICTION OF ATRIAL FIBRILLATION OCCURRENCE BASED ON ARTIFICIAL INTELLIGENCE‐ENHANCED ELECTROCARDIOGRAM TRAJECTORIES FOR AGE ESTIMATION

### 40.1 **TAEHYUN HWANG** ^1^, SENG CHAN YOU^2^, SANGYEOL KIM^1^, SUJEONG EOM^2^, DACHUNG BOO^2^, SUBIN KIM^2^, DAEHOON KIM^1^, TAE‐HOON KIM^1^, JAE‐SUN UHM^1^, HUI‐NAM PAK^1^, MOON‐HYOUNG LEE^1^, HEE TAE YU^1^, BOYOUNG JOUNG^1^

#### 40.1.1 ^1^Yonsei University College of Medicine, Seoul, Korea, Republic of,^2^Department of Biomedical Systems Informatics, Yonsei University College of Medicine,, Seoul, Korea, Republic of

**Introduction:** Artificial Intelligence (AI) estimation of age using 12‐lead electrocardiogram (ECG) data has proven effective as a biomarker for predicting future cardiac disease risks. However, the long‐term implications of temporal variations in the AI‐ECG age gap remain uncertain. This study aims to leverage the longitudinal difference between AI‐ECG predicted ages and actual ages to follow up and evaluate whether there are notable clinical differences in the occurrence of atrial fibrillation (AF) among specific groups.

**Methods:** From the patients not utilized for the AI‐ECG age prediction model (121,702 individuals with 522,261 ECGs), 5,317 individuals with 24,368 ECGs, who had both an index ECG and a hinge ECG in the fifth year, with at least one intervening ECG, were included for analysis. The absolute age gap, defined as the absolute difference between the ECG‐predicted age and the chronological age, was restricted to ±10 years for this analysis. Latent class trajectory modeling was utilized to categorize groups based on the AI‐ECG age gap.

**Results:** A data‐driven analysis identified four distinct trajectory groups based on age gap trajectories: Consistently Young ECG (28.7%), Reversed ECG Aging (35.5%), Accelerated ECG Aging (8.0%), and Consistently Old ECG (27.9%). Both the Accelerated ECG Aging group (adjusted hazard ratio (HR) 1.73, 95% confidence interval (CI) 1.07‐2.79, p‐value =0.025) and the Consistently Old ECG group (adjusted HR 1.78, 95% CI 1.27‐2.49, p‐value <0.001) exhibited significantly higher cumulative incidences of AF compared to the Consistently Young ECG group. Among the assessed candidate predictors, baseline age, chronic kidney disease, diabetes mellitus, and hypertension were identified as four independent predictors of these trajectories.

**Conclusions:** Of the four distinct trajectories derived from changes in the AI‐ECG age gap, Accelerated ECG Aging and Consistently Old ECG trajectories were linked to an increased risk of AF occurrence.

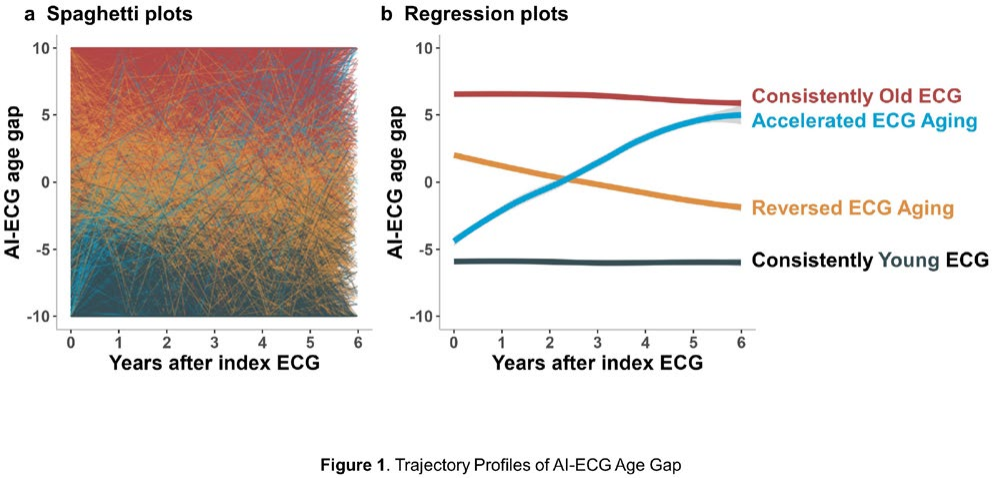

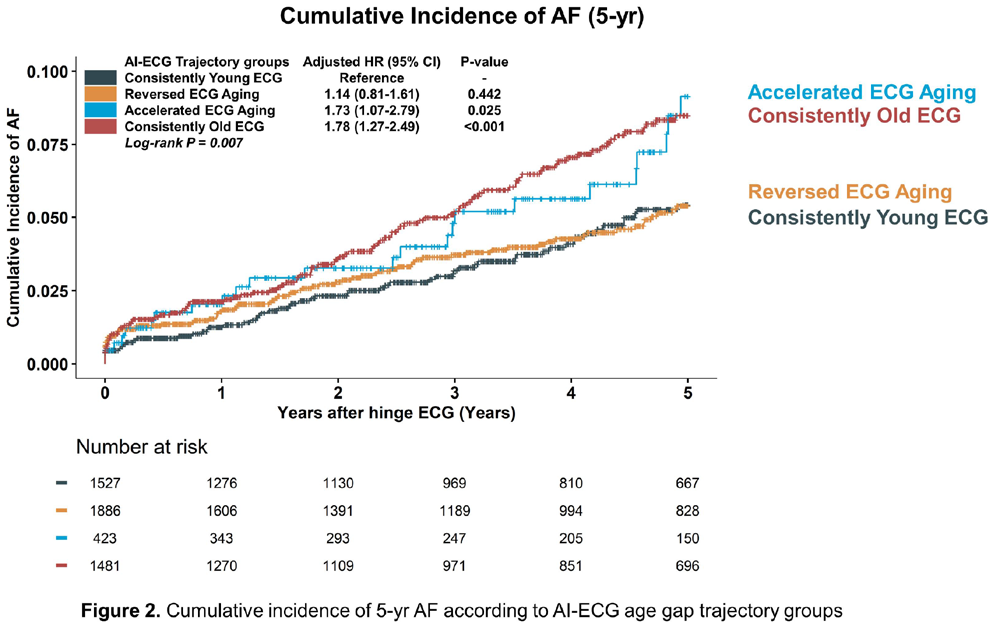

## 41 THE IMPACT OF THE EARLY INVASIVE RHYTHM MANAGEMENT ON SYMPTOMATIC HEART FAILURE WITH ATRIAL FIBRILLATION

### 41.1 **YOSHIFUMI IKEDA**, RITSUSHI KATO, HITOSHI MORI, KAZUO MATSUMOTO

#### 41.1.1 Saitama Medical University International Medical Center, Hidaka, Japan

**Introduction:** Cathter ablation(CA) for heart failure(HF) patients with atrial fibrillation(AF) has garnered considerable attention since the publication of the CASTLE‐AF trial. However, the optimal timing for such intervention remains uncertain. This study aims to elucidate the disparities between early and delayed ablation strategies in this patient population.

**Methods:** We performed a retrospective analysis of 100 consecutive cases of CA in patients presenting with symptomatic HF and AF at our institution between 2018 and 2022. Among these cases, 44 underwent CA within 30 days of HF onset (designated as the early strategy group, E group), while the remaining 56 cases were managed using conventional methods (designated as the delayed strategy group, D group).

**Results:** The mean follow‐up duration was comparable between both groups (E group: 553.8 ± 427.8 days vs. D group: 466.6 ± 353.7 days, p = 0.37). The majority of patients presented with persistent AF, with a similar proportion of patients exhibiting paroxysmal AF between the two groups (E group: 16% vs. D group: 30%, p = 0.09). The prevalence of HF with reduced ejection fraction (HFrEF) was significantly higher in the early strategy group, although the mean left ventricular ejection fraction (LVEF) was comparable between the groups (HFrEF rate: E group: 72% vs. D group: 52%, p = 0.04; mean LVEF: E group: 37.7 ± 16.2% vs. D group: 40.1 ± 15.0%, p = 0.31). The recurrence rate of AF did not differ significantly between the groups (E group: 16% vs. D group: 20%, p = 0.88), and overall survival rates were similar (Figure: Log rank test p = 0.49). While the length of hospital stay was comparable, the total number of hospitalizations was significantly reduced in the early strategy group (Table). Mortality occurred exclusively in the early strategy group (Table).

**Conclusions:** Early invasive rhythm management for symptomatic AF in the setting of HF appears to be feasible in terms of efficacy and safety, and it reduces hospitalizations compared to delayed conventional strategies. However, this approach may involve patients with more severe conditions, necessitating careful patient selection.

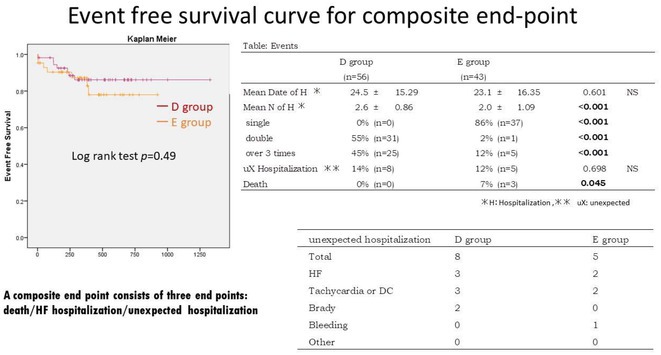

## 42 GENDER DIFFERENCES IN MANAGEMENT OF PATIENTS UNDERGOING CATHETER ABLATION OF SUPRA VENTRICULAR TACHYCARDIA

### 42.1 IFRAH UROOS, **GHAZALA IRFAN**

#### 42.1.1 National Institute of Cardiovascular Diseases, Karachi, Pakistan

**Introduction:** Gender differences in SVT prevalence, symptomatology, and management have gained attention, with disparities noted in diagnosis, treatment, and healthcare utilization. However, local data on gender disparities in SVT management remain limited.

**Methods:** A prospective cohort study was conducted on adult patients who underwent successful catheter ablation for SVT from November 2022 to May 2023 at a National Institute of Cardiovascular Diseases in Karachi, Pakistan. Data on demographics, symptom presentation, delays in seeking medical help, and patient preferences regarding physician gender were collected and analyzed using descriptive statistics and multivariable regression analysis.

**Results:** The study included 198 patients, with a higher proportion of females (68.2%) compared to males (31.8%). While both male and female patients experienced delays in informing family members about symptoms, the median delay time was longer for females. Similarly, delays in seeking medical attention after symptom onset were prevalent, with male patients exhibiting a median delay time of 0.5 years, compared to 8 years for females. These delays were often attributed to factors such as infrequent symptoms (24.7% of males and 49.2% of females), lack of knowledge (23.7% of males and 26.7% of females), and affordability issues (9.1% of males and 13.3% of females). Upon referral to an Electrophysiologist (EP) and subsequent consideration of ablation, significant delays were again evident, particularly among female patients, with 57.1% experiencing delays in visiting an EP after their first medical contact, and 86.4% experiencing delays in undergoing ablation after their first EP visit. Despite these challenges, most patients expressed no preference for physician gender, with 85.9% indicating openness to seeing any physician.

**Conclusions:** Gender disparities in SVT management were evident, with women facing greater delays in seeking and receiving care. Addressing these disparities through targeted interventions can improve healthcare equity and outcomes for both male and female patients affected by SVT

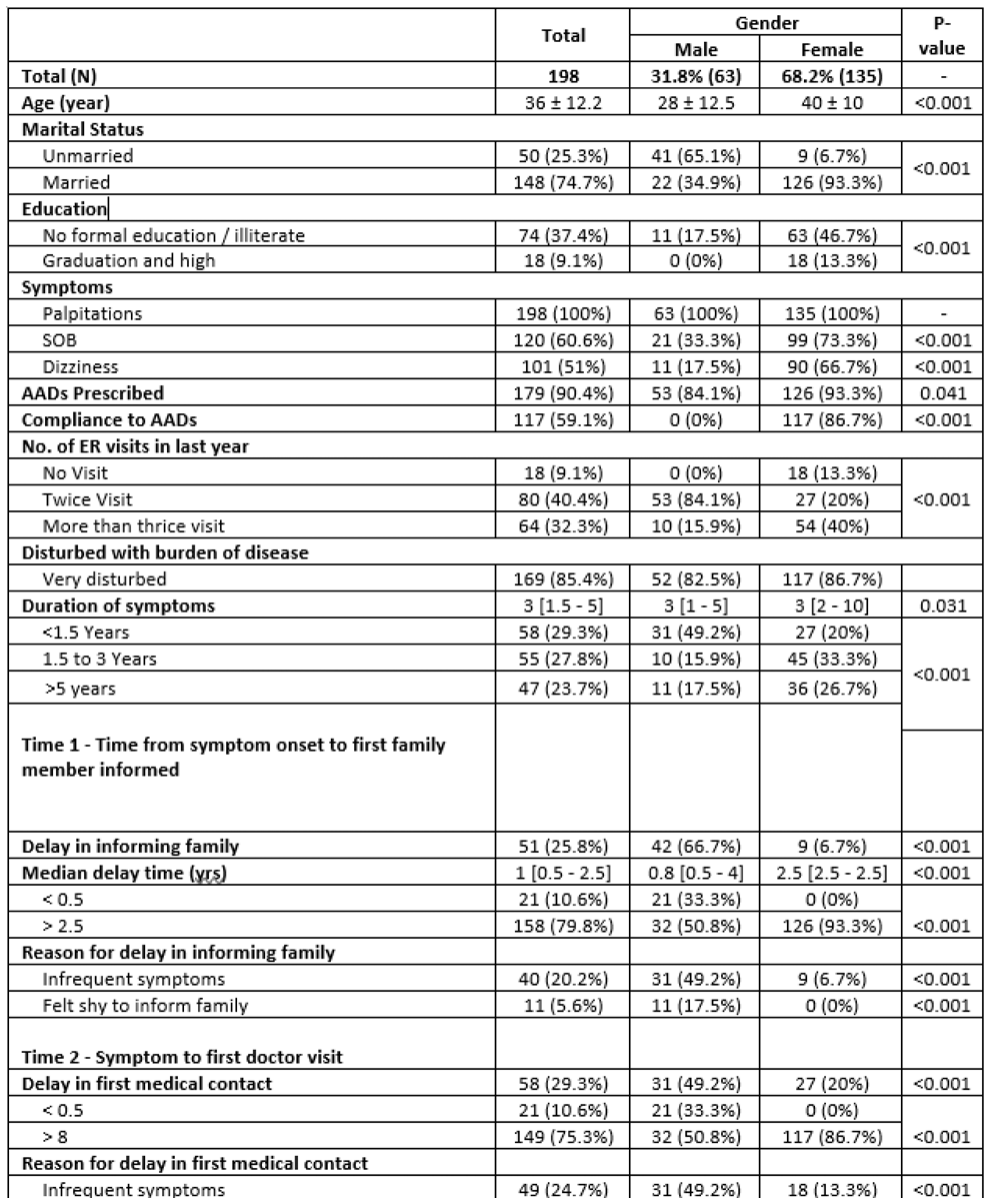

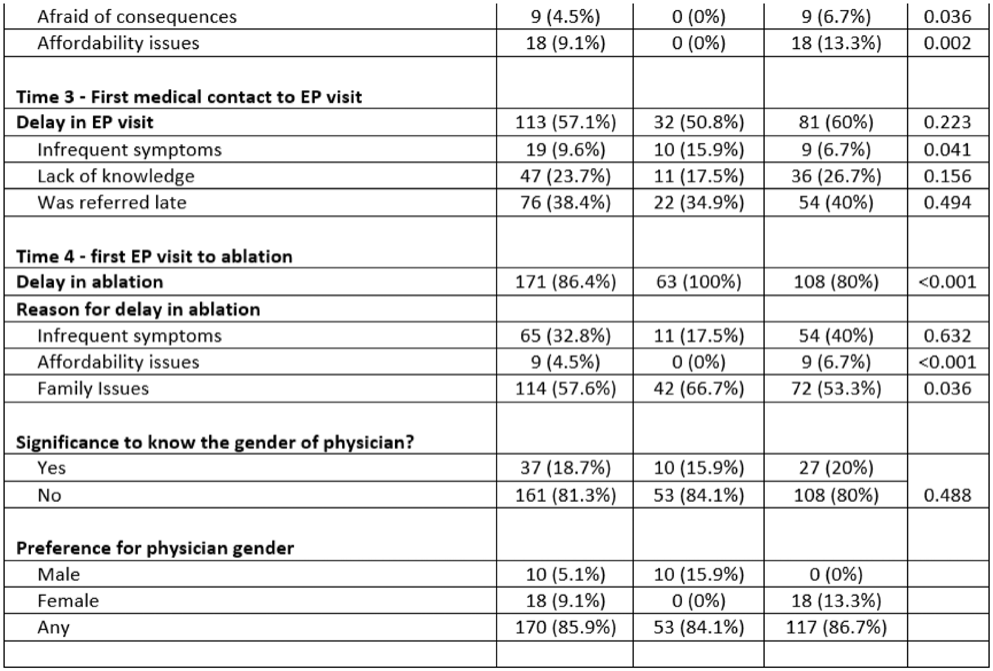

## 43 ASSSSMENT OF AV NODE DYSFUNCTION AND PACEMAKER NECESSITY FOLLOWING TRANSCATHETER AORTIC VALVE IMPLANTATION: A RESTROSPECTIVE ANALYSIS AND FUTURE DIRECTIONS

### 43.1 **FUYUE JIANG**, HANNAN KEMPTON, RACHEAL HALL

#### 43.1.1 St Vincent's Hospital, Sydney, Australia

**Introduction:** The necessity for permanent pacemaker implantation (PPI) following transcatheter aortic valve implantation (TAVI) is a critical aspect of both procedural planning and long‐term management of patients with severe aortic stenosis. Notably, there is no definitive correlation between pre‐existing right bundle branch block (RBBB) and the requirement for pacemaker implantation post‐TAVI. Consequently, it is not routine practice to implant pacemakers in the high‐risk patients prior to their TAVI procedure in our centers. During follow‐up, not all patients remain dependent on the pacemaker. Pacemaker dependency is defined as the absence of an escape or intrinsic rhythm for 30 seconds when temporary back‐up pacing is set at a rate of 30 beats per minute. This dependency occurs despite the necessity for pacing due to complete heart block in the immediate post‐procedural period.

**Methods:** We conducted a retrospective review of all patients in our TAVI program across two centers, comparing those who required PPI within 30 days post‐TAVI with their pacing dependency rates at 12‐month follow‐up.

**Results:** From August 2008 to August 2023, 1150 TAVI procedures were performed at our centers, with 113 patients (12.3%) requiring pacemaker implantation within 30 days post‐TAVI. Among these patients, 70 (7.7%) had a pre‐existing RBBB, and 12 out of these 70 patients (17%) required pacemaker implantation post‐TAVI. At the 6‐12 month follow‐up, 44 (38.9%) of all patients exhibited significant recovery of AV node conduction and were no longer pacing‐dependent, excluding 6 patients who had a cardiac resynchronization device.

**Conclusions:** Conduction disturbances are common following TAVI, highlighting the need for further prospective research to identify markers that predict the risk of requiring a pacemaker post‐TAVI

## 44 OUTCOMES OF ATRIAL FIBRILLATION ABLATION IN DRUG NAïVE PATIENTS VS PATIENTS WITH FAILED ANTI‐ARRHYTHMIC DRUGS

### 44.1 **NATASHA JONES‐LEWIS** ^1,2^, LUKAH Q. TUAN^1,2,3^, LORI BELL^1,2^, ADRIANA TOKICH^1,2,3^, JENISH SHROFF^1,2,3^, ANUGRAH NAIR^1,2,3^, RAJEEV K. PATHAK^1,2,3,4^

#### 44.1.1 ^1^Canberra Heart Rhythm, Canberra, Australia,^2^Canberra Heart Rhythm Foundation, Canberra, Australia,^3^Australian National University, Canberra, Australia,^4^University of Canberra, Canberra, Australia

**Introduction:** Atrial Fibrillation (AF) is one of the most common atrial arrhythmias involving the left atria. Radiofrequency catheter ablation along with anti‐arrhythmic drugs (AAD) have been widely used as a method of treatment. However, some patients are unable to tolerate long term AAD use or have contraindications preventing the use of AADs, including asthma, coronary artery disease or age.

**Methods:** Of 314 patients between 2018‐2024, 69 patients with symptomatic persistent AF underwent catheter ablation and were included in this study. Echocardiographic data and Holter data were analysed at baseline prior to the first ablation, early follow‐up and 1 year post ablation in patients who were not started on AAD medication (Group 1) and patients who failed AAD medication (Group 2).

**Results:** The mean age was (67±9.5) years old with 37% female. In group 1, 8 patients (19%) had diabetes, 15 (36%) had obstructive sleep apnoea and 22 patients (53%) had coronary artery disease shown on cardiac CT scan. In group 2, 4 patients (14%) had diabetes, 8 patients (28%) had obstructive sleep apnoea and 13 patients (46%) showed coronary artery disease. The average baseline left ventricular ejection fraction (LVEF) of the patients in group 1 was 59.3±9.6, compared to those patients that weren’t started on AAD's with an average LVEF of 61.2±5.8. At final follow up, LVEF remained the same in group 1 with an average of 58.7±6.6, compared to an increased LVEF of 61.4±4.4 in group 2. 3(9.6%) of patients with failed AAD, showed recurrence on Holter at 12 months post ablation compared to 1(2.3%) patient in those not started on AAD before catheter ablation.

**Conclusions:** Patients with failed AAD medications had higher recurrence of AF post ablation compared to patients who are drug naïve. Those who had failed drugs also had a higher need for device implant prior to ablation due to pauses and symptomatic bradycardia.

## 45 FEASIBILITY AND SAFETY OF MRI SCANS IN MIXED MANUFACTURER AND NON‐MRICONDITIONAL CARDIAC IMPLANTABLE ELECTRONIC DEVICES; A LARGE TERTIARY CENTREEXPERIENCE

### 45.1 **KADHIM KADHIM**, KATHARINE NELSON, CHRIS PLUMMER

#### 45.1.1 Freeman Hospital, The Newcastle Upon Tyne Hospitals Trust, United Kingdom

**Introduction:** The demand for magnetic resonance imaging (MRI) scans in patients with cardiac implantableelectronic devices (CIEDs) is increasing. A high proportion of these patients have abandoned leads or leads & generators from different manufacturers, resulting in lack of MRI conditional labelling & potentially compromising diagnostic work‐up

**Methods:** We aimed to assess the feasibility & safety of MRI scans in patients with fully MRI‐conditional systems (group A) compared to mixed manufacturers (group B), non‐conditional systems (group C), and those with epicardial/abandoned leads (group D). We analysed prospectively collected data on MRI scans performed in patients with CIEDs at Newcastle Hospitals, UK between Oct 2012 and Oct 2023. The primary endpoint was a composite of death, clinical adverse events, lead or generator failure, electrical reset & arrhythmia. The secondary endpoints comprised significant scan‐related changes in generator or lead parameters.

**Results:** There were 518 1.5T MRI scans performed in 388 patients (32% females, mean age 69.3±14.7years, range 0.6‐100.7). Scans for patients with fully‐MRI conditional systems (group A) accounted for 44.2% of total scans, with 23.9%, 27.4% and 4.4%for groups B, C & D respectively. The primary endpoint did not occur in any scan. Acute battery voltage reduction by ≥0.04V occurred in 6 (1.2%) scans (1 in group A, 5 in group C, P=0.049). There was no P or R wave amplitude reduction by ≥50%, and no atrial or ventricular threshold increase by ≥1V. A threshold increaseby ≥0.5V was noted in right ventricular (RV) leads in 8 scans (3, 1, 3 & 1 in groups A, B, C & D respectively,P=0.569) and 1 left ventricular lead (group C). Atrial lead impedance changes (increase or decrease) by ≥50Ωoccurred in 5.2% of scans (4.4%, 5.6%, 7% & 0% in groups A, B, C & D respectively, P=.534).

**Conclusions:** MRI scans in this large cohort of CIED patients were feasible and safe. Further studies are required to quantify the MRI safety of patients with non‐conditional CIED systems.

## 46 PULMONARY VEIN INTERVENTION FOR SEVERE PULMONARY VEIN STENOSIS AFTER ATRIAL FIBRILLATION ABLATION

### 46.1 **TOMONORI KATSUKI**, KOUMEI ONUKI, HIROYUKI KOUNO, REI KUJI, KENGO KORAI, MASATO FUKUNAGA, MICHIO NAGASHIMA, YOSHIMITSU SOGA, KENICHI HIROSHIMA, KENJI ANDO

#### 46.1.1 Kokura Memorial Hospital, Kitakyushu, Fukuoka, Japan

**Introduction:** Pulmonary vein stenosis (PVS) following PV isolation for ablation of atrial fibrillation is a rare complication, while the Japanese data of intervention for PVS is insufficient.

**Methods:** Between 2011 and 2023, endovascular therapy for denovo severe PVS lesion was performed for 21 patients (28 lesions) in our hospital. we evaluated the clinical outcomes after the intervention.

**Results:** The mean age was 53.3±13.8 years old, 76% was male, and a half of patients showed bloody phlegm or shortness of breath. Prior ablations were performed by radiofrequency in 22 leasions, cryo balloon in 3 leasions, and lazer balloon in 3 leasions, respectively. A total of 53% leasions were located in the left inferior pulmonary vein, and 67% leasions were chronic toatal occulisons. Stenting was performed in 64% leasions, while the other leasions were treated by angioplasty or drug‐coated balloon (DCB). In all cases, the lesions were successfully treated without any complucations. The mean follow‐up duration was 1.7±0.26 years. The restenosis rate after the intervention was 39% at 1 year, of these, the stenting had lower risk of restenosis compared with the angioplasty; the restenosis rate 19% at 1 year (crude hazerd ratio 0.30, confidence interval 0.11‐0.85, p=0.02). While DCB was used for 2 denovo lesions and for 5 restenosis lesions in 2023. During the follow‐up (mean follow‐up duraiton: 0.34±0.18 years), the patency was confirmed in all cases but one case.

**Conclusions:** We performed the intervention for PVS, and evaluated the prognosis. Even though after stenting, Restenosis occurred in one‐fifth of lesions within 1 year. Short‐term outcome of DCB showed promissing results, and the strategy has possibility to be the optimal choice for intervention. We report the clinical data with further follow‐up data to discuss the optimal PVS management.

## 47 CURRENT PRACTICE IN CATHETER ABLATION OF VENTRICULAR TACHYCARDIA: RESULTS OF THE APHRS SURVEY

### 47.1 **DAEHOON KIM** ^1^, SURINDER KAUR^2^, PIPIN KOJODJOJO^3^, NWE NEW^4^, TAKANORI YAMAGUCHI^5^, VAN DANG NGUYEN^6^, AMY CHU^7^, JAE‐MIN SHIM^8^

#### 47.1.1 ^1^Yonsei University College of Medicine, Seoul, Korea, Republic of,^2^National Heart Institute, Kuala Lumpur, Malaysia,^3^Asian Heart and Vascular Centre, Singapore, Singapore,^4^Yangon General Hospital, Yangon, Myanmar,^5^Saga University, Saga, Japan,^6^Tam Duc Heart Hospital, Ho Chi Minh City, Viet Nam,^7^United Christian Hospital, Hong Kong, China,^8^Korea University, Seoul, Korea, Republic of

**Introduction:** Ventricular arrhythmia is a leading cause of sudden cardiac death and is associated with increased mortality and morbidity, particularly in patients with structural heart disease (SHD). The Asia Pacific Heart Rhythm Society (APHRS) Ablation Committee conducted a survey to explore procedural specifics, applied technologies, ablation strategies, and procedural endpoints in ventricular arrhythmia ablation.

**Methods:** A 21‐item online questionnaire was developed and distributed among electrophysiology specialists by APHRS. A total of 84 participants from 21 different countries responded to the survey.

**Results:** More than half of the respondents indicated that their affiliated centers handle 100‐500 ablation procedures annually, of which 10‐50 are ventricular tachycardia (VT) or premature ventricular complex (PVC) ablations. The majority (65%) reported that over 60% of the patients undergoing VT/PVC ablation did not have underlying SHD. The most common etiology for VT/PVC in patients with SHD was ischemic cardiomyopathy, accounting for an average of 47.3% of cases, while the right ventricular outflow tract was the most frequent origin of VT/PVC in patients without SHD, with a mean proportion of 56.9%. Catheter ablation was the initial treatment for idiopathic VT/PVC in 52.2% of the cases. Ablation was preferred for hemodynamically stable sustained monomorphic VT in patients with impaired ventricular function by 34.5% of respondents for ischemic origins and 33.3% for non‐ischemic origins. Routine cardiac magnetic resonance imaging before VT ablation was reported by 65.5% of the respondents. Substrate modification, with or without focal ablation of the clinical VT, was the predominant ablation strategy (70.2%) for patients with SHD. An epicardial approach was used by 44% of respondents. Non‐inducibility of VT and the elimination of all local abnormal ventricular activities were the predominantly reported endpoints in VT ablation.

**Conclusions:** This APHRS survey indicates that VT management in the Asia‐Pacific region aligns with current VT guidelines. Sharing these insights and facilitating collaboration may improve patient outcomes.

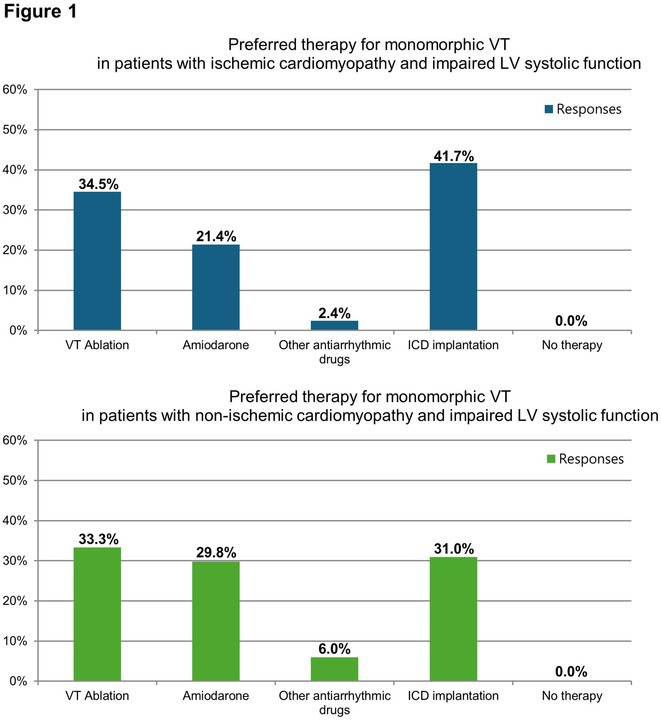

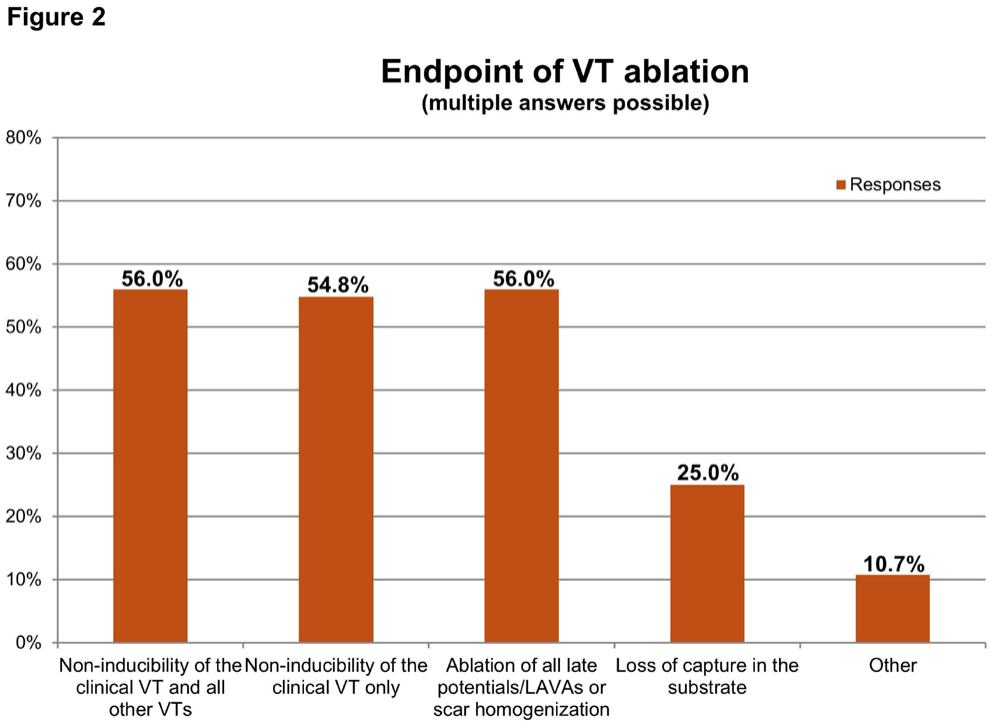

## 48 THE CLINICAL IMPLICATION OF ATRIAL FIBRILLATION IN BREAST CANCER

### 48.1 **DO YOUNG KIM** ^1^, KWANG‐NO LEE^1^, JONG‐CHAN YOUN^2^, SUNG HEA KIM^3^

#### 48.1.1 ^1^Ajou University Medical Center, Gyeonggi‐do, Korea, Republic of,^2^Seoul St. Mary's Hospital, Seoul, Korea, Republic of,^3^Konkuk University Medical Center, Seoul, Korea, Republic of

**Introduction:** Atrial fibrillation (AF) is an established CV risk factor and can impact breast cancer outcomes. Additionally, cancer‐related factors may increase risk of AF. However, there is a scarcity of studies investigating how it impacts the outcomes of patients with breast cancer.

**Methods:** The cohort of the study comprised 1,233 Asian female patients with breast cancer from 4 medical centers in Korea. We utilized Cox regression analysis was employed for survival data.

**Results:** In this study, AF was associated with an increased risk of major adverse cardiovascular events (MACE) which is a composite of myocardial infarction, stroke, heart failure, and CV death. The figure shows that patients with AF had a higher incidence of MACE compared to patients without AF. A further multivariate Cox regression model was employed to assess the clinical significance of AF in breast cancer patients. In the multiple Cox regression model, AF was associated with increased risk of MACE (hazard ratio [HR] 2.61, 95% CI, 1.29‐5.28; p=0.007), and rehospitalization (HR, 1.63, 95% CI 1.02‐2.56).

**Conclusions:** AF is associated with an increased risk of MACE in breast cancer patients. Therefore, early identifying patients with AF is necessary to optimize their outcome.

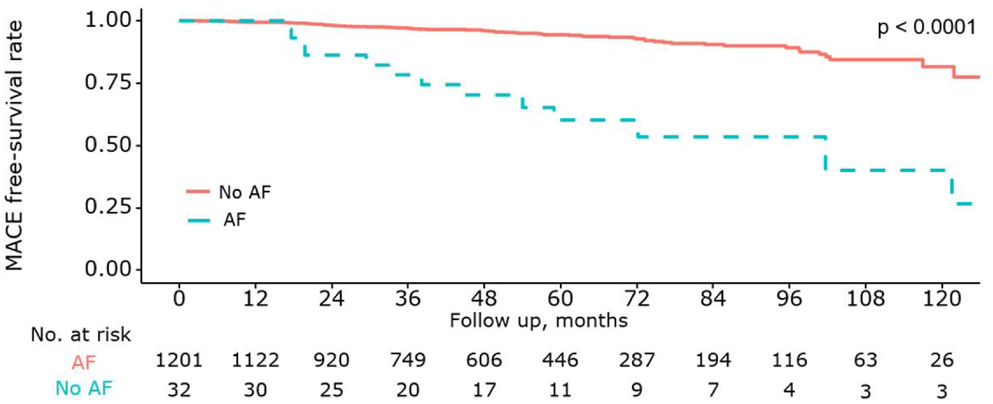

## 49 FIRST‐LINE TREATMENT OF ATRIAL FIBRILLATION WITH CATHETER ABLATION VERSUS ANTI‐ARRHYTHMIC DRUGS: RETROSPECTIVE ANALYSIS OF AN ADMINISTRATIVE CLAIMS DATABASE OF COMMERCIALLY INSURED ADULTS IN THE UNITED STATES

### 49.1 RAHUL KHANNA^1^, RHEA PIMENTEL^2^, SONIA MACCIONI^1^, DONGYU ZHANG^1^, JONATHAN PICCINI^3^, **MIRI KIM** ^1^

#### 49.1.1 ^1^Johnson & Johnson, New Brunswick, NJ,^2^The University of Kansas Health System, Kansas City, KS,^3^Electrophysiology Section, Duke University Hospital, NC

**Introduction:** There is limited information currently available on outcomes among patients with newly diagnosed atrial fibrillation (AF) undergoing first‐line treatment with catheter ablation (CA) versus anti‐arrhythmic drugs (AADs) in a real‐world setting.

**Methods:** The Optum Clinformatics Dataset, which is an administrative claims dataset for commercially insured individuals in the United States, were used for study purposes. Patients with incident AF (diagnosed between 2015‐2022) were identified and classified into CA or AAD group based on first‐line treatment received. Primary outcome of interest was 1‐year incidence of atrial tachyarrhythmia (inclusive of AF, atrial flutter, and atrial tachycardia). The CA and AAD cohorts were balanced on study covariates using inverse probability of treatment weighting (IPTW). Cox regression model was used to examine differential risk of outcome among the weighted cohorts.

**Results:** The final sample included 2,711 CA and 22,726 AAD patients. Study cohorts were well balanced on study covariates post‐IPTW. Regression analysis revealed a 42% lower risk of atrial tachyarrhythmia incidence among patients with AF who underwent first‐line treatment with CA versus AADs (hazard ratio [HR]=0.58, 95% confidence interval [CI] 0.45‐0.74). Similar results were observed when examining by AF type, with risk of atrial tachyarrhythmia lower for CA (versus AAD) cohort in both paroxysmal (HR=0.51, 95% CI 0.36‐0.73) and persistent AF (HR=0.63, 95% CI 0.42‐0.94) patients, respectively.

**Conclusions:** The use of CA as first‐line treatment among newly diagnosed patients with AF was associated with significantly lower recurrence risk of atrial tachyarrhythmia as compared to the use of AAD. Results were consistent irrespective of AF type.

## 50 SMOKING IS ASSOCIATED WITH INCREASED RISK OF SUDDEN CARDIAC ARREST IN YOUNG

### 50.1 **YEJI KIM** ^1^, JOO HEE JEONG^1^, YUN GI KIM^1^, CHANG‐OK SEO^1^, HYOUNG SEOK LEE^1^, YUN YOUNG CHOI^1^, SEUNG‐YOUNG ROH^2^, JAEMIN SHIM^1^, YOUNG‐HOON KIM^1^, KYUNG‐DO HAN^3^, JONG‐IL CHOI^1^

#### 50.1.1 ^1^Korea University Anam Hospital, Seoul, Korea, Republic of,^2^Korea University Guro Hospital, Seoul, Korea, Republic of,^3^Soongsil University, Seoul, Korea, Republic of

**Introduction:** Smoking is a strong risk factor of adverse cardiovascular event as well as sudden cardiac arrest (SCA). Regarding the different etiology of SCA in younger individuals compared to the elderly, we aimed to investigate the linkage of smoking and the risk of SCA in young adults.

**Methods:** Data was obtained from the national health screening database in South Korea. Adults aged between 20 to 39 years that underwent health screening from 2009 to 2012 were included. Smoking status was categorized as non‐smokers, ex‐smokers, and current‐smokers. Burden of smoking was divided as mild (<10 pack‐years), moderate (<20 pack‐years), and heavy smokers (≥20 pack‐years).

**Results:** A total of 6,293,672 individuals were analyzed. Mean age was 30.8 years, comorbidities such as hypertension, diabetes mellitus, or dyslipidemia were rare (7.4%, 1.9%, 1.2%, respectively). According to smoking status, non‐smokers were most prevalent (n=3,472,782, 55.2%), followed by current‐smokers (n=2,188,036, 34.8%) and ex‐smokers (n=632,851, 10.1%). Incidence of SCA increased from non‐smokers (incidence rate 0.06) to ex‐smokers (0.08) to current‐smokers (0.14). Current smoking showed cumulative effect on SCA in median 9.6 years follow‐up. Smoking burden revealed dose‐dependent relationship with SCA incidence, which showed linear increase from mild (0.10) to moderate (0.16) and heavy smokers (0.25). Adjustment of confounding risk factors showed consistently increased risk of SCA in current‐smokers (adjusted hazard ratio 1.69), which was reversed in ex‐smokers (adjusted hazard ratio 0.96).

**Conclusions:** In young‐aged adults, current smoking revealed increased risk of SCA in long‐term follow‐up. Risk of SCA showed positive correlation with cumulative burden of cigarette exposure.

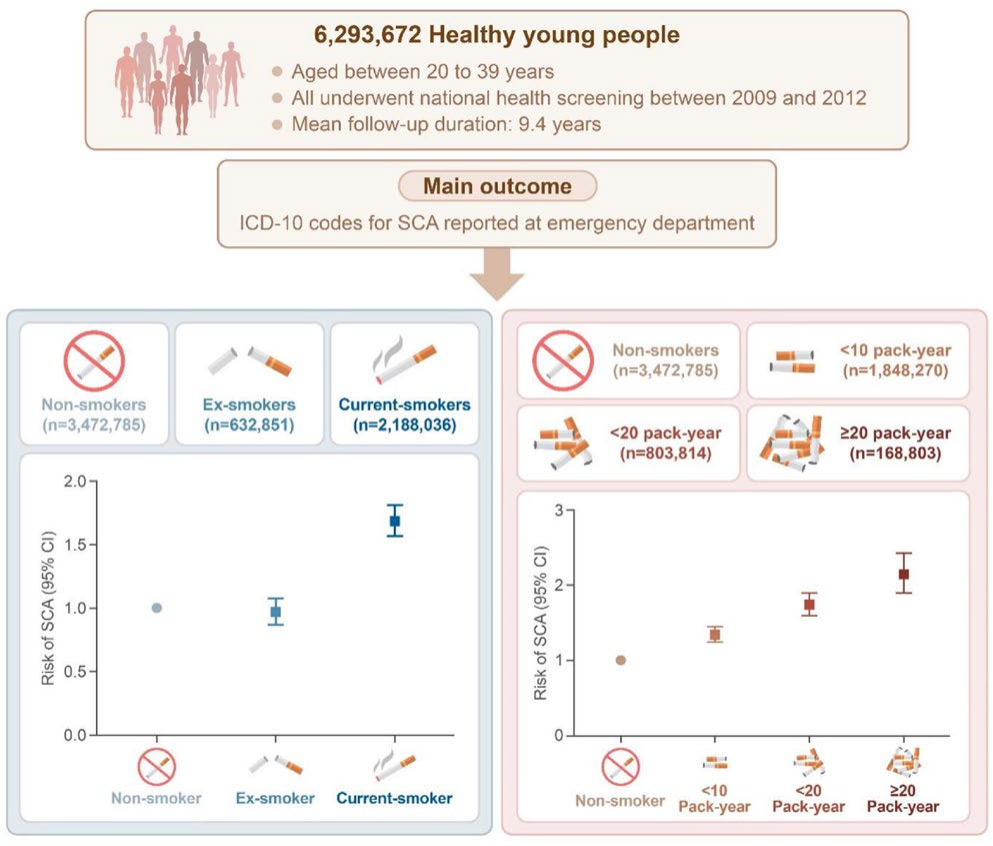

## 51 LEFT BUNDLE PACING IN A PATIENT WITH PERSISTENT LEFT SUPERIOR VENA CAVA

### 51.1 NATCHAYATHIPK KITTICHAMROEN

#### 51.1.1 Charoenkrung Pracharak Hospital, Bangkok, Thailand

**Introduction:** Left bundle pacing (LBP) has recently emerged and widely used as a novel modality for conductive system pacing. Persistent left superior vena cava (PLSVC) is a very rare anatomic variation with the prevalence in the general population is 0.1‐0.5%. Owing to the venous access of PLSVC was very distorted. Hence LBP in PLSVC is very technically challenging. Our study aimed to evaluate the feasibility of LBP in patients with PLSVC.

**Methods:** N/A

**Results:** A 79‐year‐old male who had the indication of pacemaker implantation with sick sinus syndrome was performed with LBP. PLSVC was found during the operation. The active helix stylet‐driven lead (Ingevity lead 7842‐59, Boston Scientific Inc., Marlborough, MA, USA) was used through 9184 SSPC4 delivery sheath at first. The ventricular lead always fell down into the right ventricular (RV) due to its instability. Hence 9184 SSPC4 delivery sheath was manually adjusted double‐curve shape in reverse and precisely targeted at left bundle area. The initial pacing QRS morphology had the "W" sign on lead V1. The lead was then rotated with repeatedly assessed QRS morphology and lead parameters. At the final lead position, the pacing QRS morphology had a rSr' morphology in V1 with negative QRS complex in inferior leads, compatible with left posterior fascicular pacing and time from pacing stimulus to peak R wave in lead V5 (LVAT) was 82 ms. The final lead parameters were impedance 823 ohms, R wave 9.9 mV, capture threshold 0.6 V at 0.4ms. The device was programmed as DDDR 60‐120 bpm. The intraoperative electrophysiological characteristics, pacing parameters, CT chest indicated that the electrode was fixed to the ventricular side across the tricuspid valve. The threshold of LBAP was 0.6V/0.4ms on the first day after operation, and increased to 1.3V/0.6ms at seven months follow‐up.

**Conclusions:** LBP was successfully achieved by active helix stylet‐driven lead in the patient, which revealed the conventional lead can achieve LBP via PLSVC.

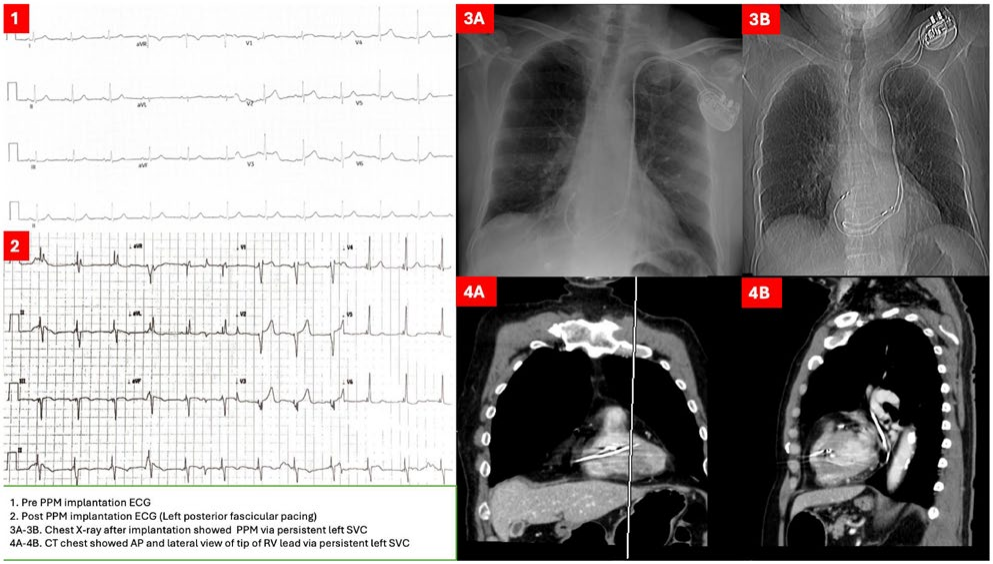

## 52 SYMPATHETIC NEUROTRANSMITTER ALTERATION IN ISCHEMIC HEART FAILURE MODEL: IMPLICATIONS FOR VENTRICULAR ARRHYTHMOGENESIS VIA CALCIUM OVERLOAD

### 52.1 **JIRO KOYA** ^1^, TARO TEMMA^1^, KEI KAWAKAMI^1^, MASAHIRO KAWASAKI^1^, KINTARO SHIMANO^1^, SHOTA SAITO^1^, DAISHIRO TATUSTA^1^, KOTARO NISHINO^1^, HIROYUKI NATSUI^1^, TAKAHIDE KADOSAKA^1^, TARO KOYA^1^, MOTOKI NAKAO^1^, MASAYA WATANABE^2^, TOSHIHISA ANZAI^1^

#### 52.1.1 ^1^Department of Cardiovascular Medicine, Faculty of Medicine and Graduate School of Medicine, Hokkaido University, Sapporo, Japan,^2^Department of Cardiovascular Medicine, Hokko Memorial Hospital, Sapporo, Japan

**Introduction:** Mechanism of fatal ventricular arrhythmias (VA) in ischemic heart failure (HF) remains elusive. Neuropeptide Y (NPY), which is co‐released with norepinephrine (NE) via sympathoexcitation, has been implicated with VAs, apart from the conventional NE‐β receptor‐mediated pathway under physiological condition. However, its role in the pathogenesis of VA in ischemic HF has not been explored. We hypothesize that NPY plays a more prominent role in inducing ventricular arrhythmias in the setting of ischemic heart failure compared to NE.

**Methods:** We used a mouse model of ischemic HF in which myocardial infarction was induced by permanent left anterior descending coronary artery ligation. Immunohistochemistry staining of NE and NPY in ventricular myocardium were assessed. Calcium (Ca^2+^) dynamics were measured in isolated cardiomyocytes using a confocal microscope. VA was studied in Langendorff‐perfused heart.

**Results:** NPY expression was significantly upregulated despite marked down‐expression of NE in the infarcted myocardium compared to sham, nevertheless both NPY and NE are sympathetic neurotransmitters. Interestingly, NPY expression in the border and remote zone was significantly upregulated on the endocardial side, not on the epicardium, compared to sham. The frequency of Ca^2+^ waves and the percentage of cells with Ca^2+^ waves were significantly increased in the NPY‐treated group, showing a mechanism of Ca^2+^ overload. Functionally, NPY administration significantly increased the premature ventricular contraction (PVC) burden and the appearance of complex PVCs in Langendorff‐perfused hearts in HF mice compared to sham. NPY enhanced VA inducibility and the myocardium in the pathogenesis condition changed the sensitivity of NPY.

**Conclusions:** NPY expression was clearly elevated compared to NE, that we called “neurotransmitter alternance”, in an ischemic HF model. NPY stimulation in this model resulted in increased ventricular arrhythmogenicity via Ca^2+^ overload. NPY may be a potential target of therapeutic interventions in VA of ischemic HF.

## 53 MULTICENTER AUTOMATIC DEFIBRILLATOR IMPLANTATION TRIAL ‐ SUBCUTANEOUS IMPLANTABLE CARDIOVERTER DEFIBRILLATOR: MADIT S‐ICD

### 53.1 **VALENTINA KUTYIFA** ^1^, REINOUD KNOPS^2^, DAVID CANNOM^3^, JAMES DAUBERT^4^, CLAUDIO SCHUGER^5^, JEANNE POOLE^6^, WOJCIECH ZAREBA^1^, ILAN GOLDENBERG^1^, MARY BROWN^1^, SCOTT MCNITT^1^, CHRISTOPHER BECK^7^, SCOTT SOLOMON^8^

#### 53.1.1 ^1^University of Rochester Medical Center, Rochester, NY,^2^Academic Medical Center, Amsterdam, Netherlands,^3^Good Samaritan Hospital, Los Angeles, NY,^4^Duke University, Durham, NY,^5^Henry Ford Hospital, Detroit, NY,^6^University of Washington, Seattle, NY,^7^Department of Biostatistics and Computational Biology, University of Rochester Medical Center, Rochester, NY,^8^Brigham and Women's Hospital, Boston, NY

**Introduction:** Patients with diabetes mellitus, myocardial infarction (MI), older age, and relatively preserved left ventricular ejection fraction (LVEF) are at risk for sudden cardiac death (SCD). However, it is not currently known whether implantation of an S‐ICD is associated with an improved survival in patients with diabetes mellitus and a relatively preserved LVEF of 36 to 50%.

**Methods:** The MADIT S‐ICD trial was designed to test the hypothesis that post‐MI patients age>65 years with diabetes, and an LVEF of 36‐50% will have a survival benefit from a subcutaneous implantable cardioverter defibrillator (S‐ICD) as compared to conventional medical treatment (CMT). The study aimed to enroll 1800 subjects at 100 European and US centers. Patients were randomized to S‐ICD vs. CMT in a 2:1 ratio and followed for a minimum of 5 years. The primary endpoint was all‐cause mortality, secondary endpoints included the effects of S‐ICD vs. CMT on SCD. The primary analysis was performed on intention‐to‐treat principles.

**Results:** The MADIT S‐ICD trial enrolled 40 subjects between 2017 and 2018 when it was stopped early. The mean age of the patients was 70.9±4.7 years, the mean LVEF was 42.9±3.8%. S‐ICD was associated with a numerically lower, 15% cumulative probability of mortality, as compared to 28% probability of mortality in the CMT arm (log‐rank p=0.344), associated with a non‐significant, 51% risk reduction in all‐cause mortality (HR=0.49, 95% CI:0.11‐2.20, p=0.354). The SCD rate at 5 years was 11% in the CMT arm vs. 4% in the S‐ICD arm (p=0.461) (HR=0.37, 95% CI: 0.02‐5.88, p=0.479) (Figure). The 5‐year cumulative probability of S‐ICD appropriate shock was 11%, the S‐ICD inappropriate shock rate was 14%. The 5‐year S‐ICD device and procedure related complication free rate was 79%.

**Conclusions:** In MADIT S‐ICD we have shown, that in patients with diabetes, prior MI, older age, and an LVEF 36‐50%, the S‐ICD was associated with a numerically lower rate of all‐cause mortality. Five‐year rates of appropriate and inappropriate S‐ICD shocks were 11% and 14%, respectively.

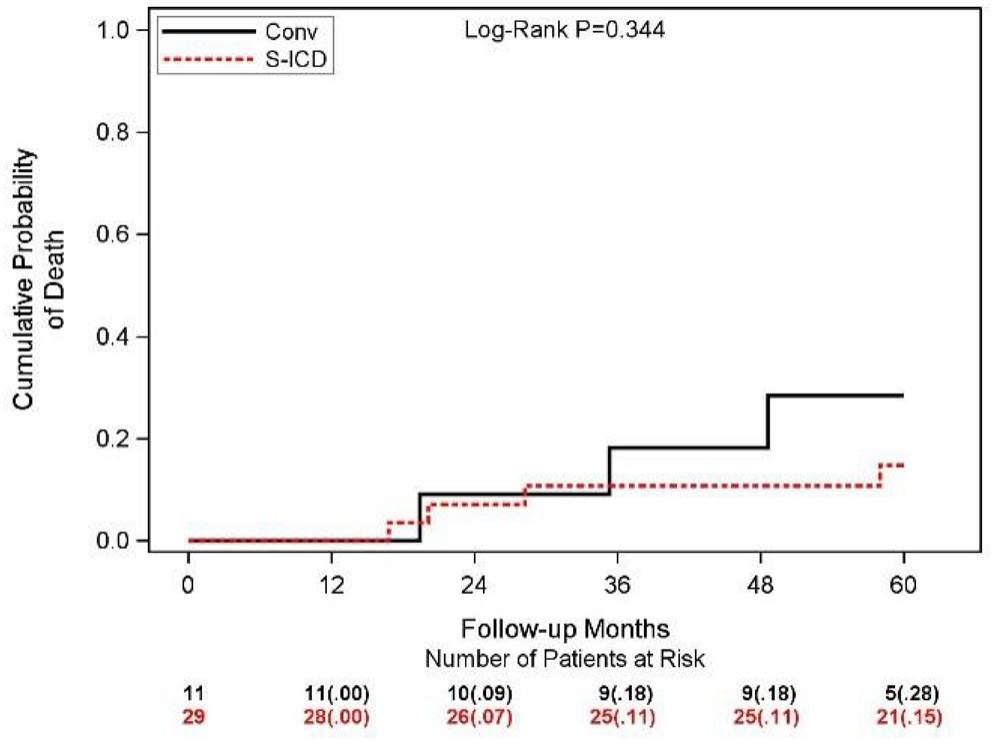

## 54 TWO CASES OF CATHETER ABLATION FOR ATRIAL FIBRILLATION AND FLUTTER THROUGH HEPATIC VEIN IN PATIENTS WITH LEFT ISOMERISM AND INFERIOR VENA CAVA INTERRUPTION

### 54.1 **GON LEE**, JAE‐SUN UHM, WON WOO YOO, JI‐YOUN CHOI, DUK‐WOO PARK, HANJIN PARK, DAEHOON KIM, HEE TAE YU, TAE‐HOON KIM, BOYOUNG JOUNG, HUI‐NAM PAK, MOON‐HYOUNG LEE

#### 54.1.1 Yonsei University Severance Cardiovascular Hospital, Seoul, Korea, Republic of

**Introduction:** In common atrial fibrillation catheter ablation cases, we use both the femoral vein for positioning catheters and septal puncture. However, some patients have vascular abnormalities. In this abstract, there are two cases where the hepatic vein was used for positioning the catheter and successfully performing the ablation.

**Methods:** N/A

**Results:** A 54‐year‐old female was referred for radiofrequency catheter ablation (RFCA) for atrial fibrillation (AF). Chest and abdomen computed tomography revealed inferior vena cava (IVC) interruption with azygos continuation, polysplenia, and bilateral left atria, bronchi, and lungs corresponding to left isomerism. RFCA for AF was decided to be performed through a hepatic vein approach. The hepatic vein was punctured under ultrasound guidance, and a Schwarz sheath was inserted into the right atrium (RA) (Figure A and B). After septal puncture, RFCA for the antrum of the four pulmonary veins was performed, and electrical isolation was achieved. After RFCA, the puncture site of the hepatic vein was occluded with a vascular plug.

A 53‐year‐old male complained of palpitations and dyspnea. He was diagnosed with situs inversus, left isomerism (IVC interruption with azygos continuation), and ventricular septal defect (VSD). RFCA for AF was decided to be performed through a hepatic vein approach. The hepatic vein was punctured under ultrasound guidance, and a Schwarz sheath was inserted into the RA. After septal puncture, RFCA for the antrum of the four pulmonary veins was performed, and electrical isolation was achieved. After RFCA, the puncture site of the hepatic vein was occluded with a vascular plug. However, Atrial flutter recurred 1 month after RFCA. Second RFCA for atrial flutter was decided to be performed through a hepatic vein approach. The 3‐dimensional activation map showed dual‐loop RA macro reentrant tachycardia (Figure C). RFCA was performed at the Cavo‐tricuspid isthmus and scar to IVC.

**Conclusions:** In conclusion,The hepatic vein approach is a good solution for patients with IVC interruption with azygos continuation.

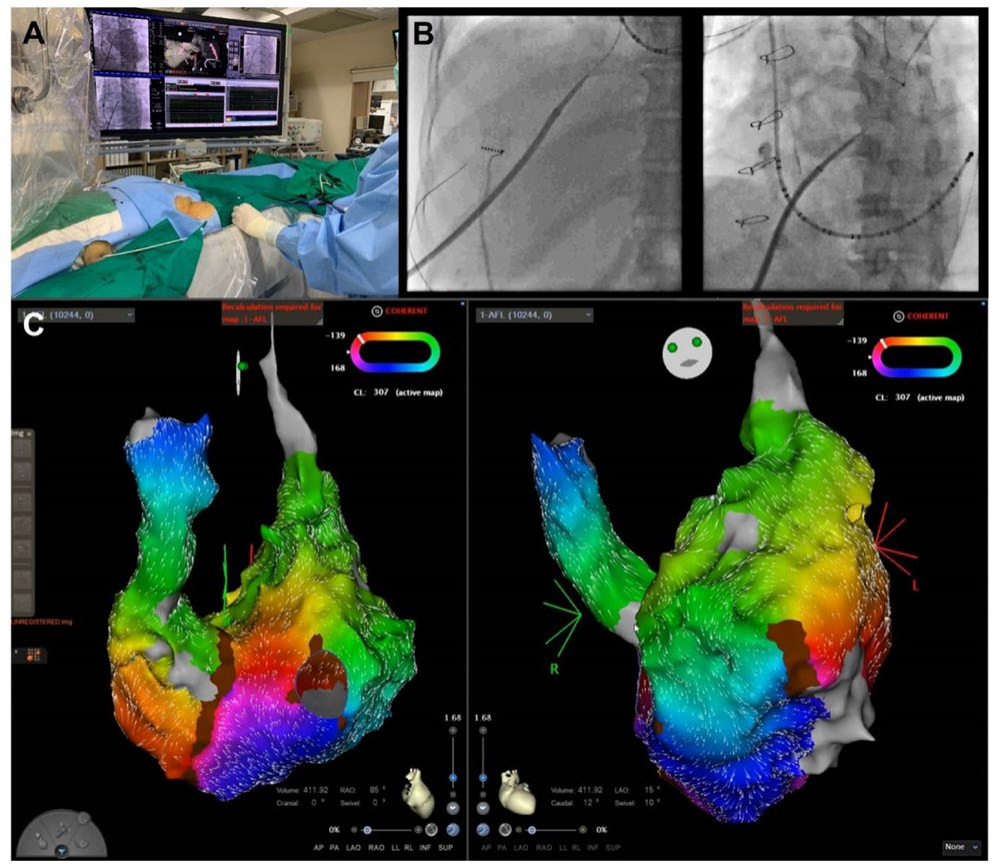

## 55 ATRIAL FIBRILLATION IN ADULT PATIENTS WITH ATRIAL SEPTAL DEFECT: A NATIONWIDE POPULATION‐BASED STUDY

### 55.1 **JUE SEONG LEE** ^1^, MAN YOUNG PARK^2^, JIN‐MAN JUNG^3^, HONG JU SHIN^4^

#### 55.1.1 ^1^Korea University Anam Hospital, Seoul, Korea, Republic of,^2^Digital Clinical Research Department, Korea Institute of Oriental Medicine, Daejeon, Korea, Republic of,^3^Korea University Ansan Hospital, Ansan, Korea, Republic of,^4^Myoungju Hospital, Yongin, Korea, Republic of

**Introduction:** The beneficial effect of atrial septal defect (ASD) closure on cardiovascular events and mortality in adults remains unclear. This study aims to investigate the effects of different ASD closure methods on atrial fibrillation (AF) in adults.

**Methods:** We retrospectively investigated the Korean National Health Insurance Service benefit records from 2002 to 2020. Among them, we extracted patients aged 20 years or older who were newly diagnosed with ASD based on diagnostic codes between 2004 and 2015. The participants were divided into the observation, device, and surgery groups. Subsequently, propensity score matching (PSM) was performed in a ratio of 2:1:1 for each group based on sex, age, cardiovascular diseases and medications. The observation group was defined based on the diagnosis date, while the device/surgery group was defined based on the ASD closure date as the index date. The occurrence of cardiovascular outcomes including AF was compared among the groups during the follow‐up period.

**Results:** A total of 20,643 ASD patients were included in this study, with 6,636 patients assigned to the observation group and 3,318 patients each assigned to the device and surgery groups following PSM. Over a 5‐year follow‐up period, the adjusted hazard ratio (aHR) for AF were significantly lower in the device closure group (0.64; 95% CI, 0.52‐0.78) compared to the observation group, while they were significantly higher in the surgical group (1.34; 95% CI, 1.14‐1.58) compared to the observation group. Furthermore, when comparing the short‐term outcomes during the three months immediately following the procedure, the device closure group also exhibited significantly lower aHR (0.30; 95% CI, 0.21‐0.43) compared to the surgical group.

**Conclusions:** In adult patients with ASD, the occurrence of AF demonstrated different prognoses depending on the treatment method. The device closure group showed the most favorable outcome for AF, while the surgery group exhibited a higher risk of AF compared to the observation group.

## 56 EXPLORING THE ROLE OF MIR‐122 IN ION CHANNEL REGULATION AND MITOCHONDRIAL PROTECTION IN LA CARDIOMYOPATHY

### 56.1 **GUAN‐YI LI** ^1^, SHIH‐LIN CHANG^2^, FA‐PO CHUNG^2^, YENN‐JIANG LIN^2^, LI‐WEI LO^2^, YU‐FENG HU^2^, CHIN‐YU LIN^2^, TING‐YUNG CHANG^2^, YU‐CHENG HSIEH^1^, CHENG‐HUNG LI^1^, YU‐SHAN CHIEN^1^, MING‐REN KUO^1^, SHANG‐JU WU^1^, SHIH‐ANN CHEN^1^

#### 56.1.1 ^1^Taichung Veterans General Hospital, Taichung, Taiwan,^2^Taipei Veterans General Hospital, Taipei, Taiwan

**Introduction:** The potential targets of microRNA (miR) ‐122 encompass genes associated with calmodulin 3. However, existing evidence regarding the functional role of this miR in LA cardiomyopathy remains exceedingly limited. The aim of the present study was to explore the impact of miR‐122 on various ion channels and the corresponding molecular mechanisms in the development of LA cardiomyopathy. Additionally, we examine this effect at the mitochondrial level.

**Methods:** H9c2 cells, derived from embryonic rat cardiomyocytes, underwent transfection and in‐vitro miR‐122 expression manipulation using a lentiviral expression vector. The expression of calmodulin 3 and various ion channels in H9c2 cells were measured, both with and without miR‐122 transfection. Furthermore, a hypoxic rat model was employed to induce LA cardiomyopathy, and the impact of miR‐122 transfection on the mitochondria was examined.

**Results:** Calmodulin 3 expression was significantly lower in H9c2 cells with miR‐122 transfection (P < 0.001, Figure A). The expression of ion channels Cav1.2, SERCA2a, NCX, and PLB decreased significantly (P < 0.01 for each), while the expression of ion channels Nav1.5, KvLQT1, and RYR did not show significant changes following miR‐122 transfection (P > 0.05 for each, Figure B). In hypoxic H9c2 cells, both mitochondrial D‐loop and 18s rRNA levels were significantly diminished, whereas NF‐κB level was significantly elevated (P < 0.01, < 0.001, and < 0.001 respectively, Figure C). Interestingly, levels of mitochondrial D‐loop and 18s rRNA were notably higher, while NF‐κB was notably lower in hypoxic H9c2 cells transfected with miR‐122 compared to those without transfection (P < 0.05 for all, Figure C).

**Conclusions:** MiR‐122 regulates ion channels in cardiomyocytes, notably influencing calmodulin 3 and calcium handling. A protective effect of miR‐122 on the mitochondrion was also observed. These findings may have implications for the molecular mechanisms underlying LA cardiomyopathy.

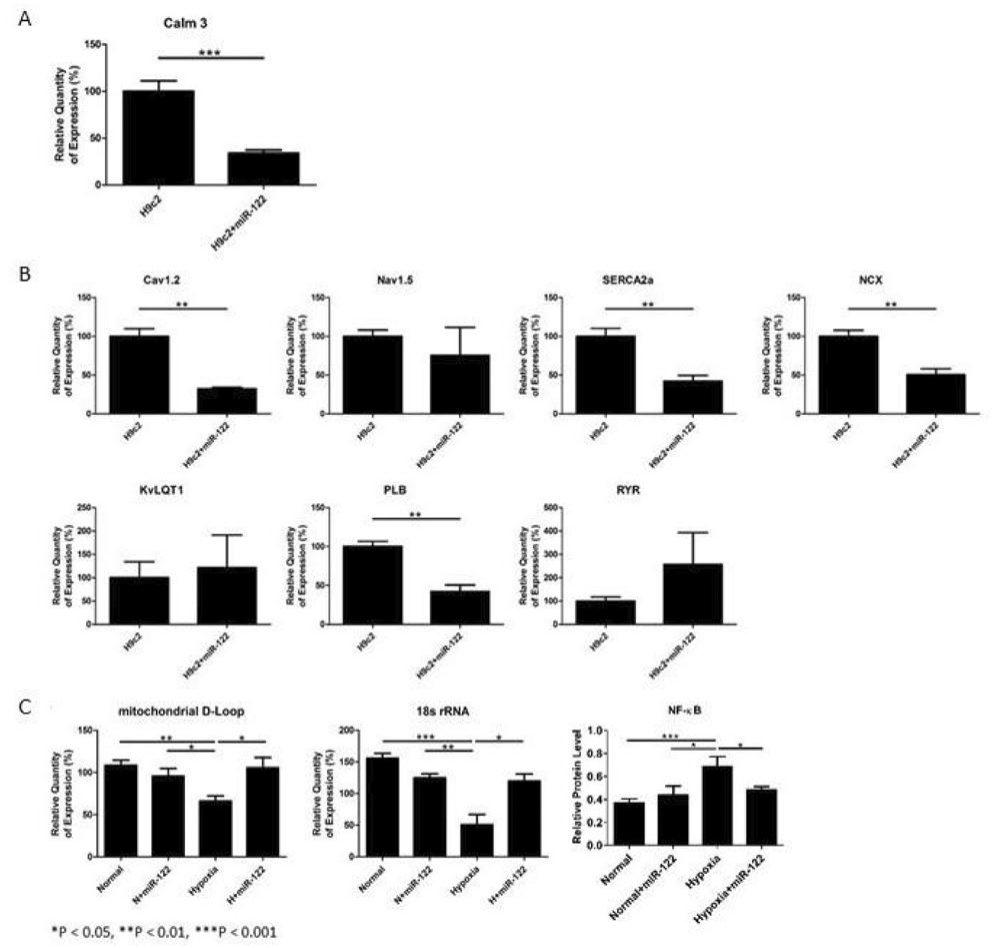

## 57 DYSSYNCHRONOUS HEART FAILURE MODELS IN CANINES: NEW INSIGHTS INTO ELECTROCARDIOGRAPHIC, ECHOCARDIOGRAPHIC AND HISTOLOGICAL FEATURES

### 57.1 **HUI LI**, HAN JIN, HAO HUANG, SIJING CHENG, YIRAN HU, WEI HUA

#### 57.1.1 Fuwai Hospital, Chinese Academy of Medical Sciences and Peking Union Medical College, Beijing, China

**Introduction:** We investigated the similarities and differences between two experimental approaches using tachy‐pacing technology to induce desynchronized heart failure in canines.

**Methods:** A total of eight dogs were included in the experiment, four were tachy‐paced in right ventricle apex (RVAP) and 4 were paced in right atrium after the ablation of left bundle branch to achieve left bundle branch block (RAP+LBBB). Three weeks of follow‐up were conducted to observe the changes in cardiac function and myocardial staining was performed at the end of the experiment.

**Results:** Both experimental approaches successfully established heart failure with reduced ejection fraction models, with similar trends in declining cardiac function. The RAP+LBBB group exhibited a prolonged overall ventricular activation time, delayed left ventricular activation, and lesser impact on the right ventricle. The RVAP approach led to a reduction in overall right ventricular compliance and right ventricular enlargement. The RAP+LBBB group exhibited significant reductions in left heart compliance (LVGLS, %: RAP+LBBB −12.60 ± 0.12 to −5.93 ± 1.25; RVAP −13.28 ± 0.62 to −8.05 ± 0.63, p = 0.023; LASct, %: RAP+LBBB −15.75 ± 6.85 to −1.50 ± 1.00; RVAP −15.75 ± 2.87 to −10.05 ± 6.16, p = 0.035). Histological examination revealed more pronounced fibrosis in the left ventricular wall and left atrium in the RAP+LBBB group while the RVAP group showed more prominent fibrosis in the right ventricular myocardium.

**Conclusions:** Both approaches establish HFrEF models with comparable trends. The RVAP group shows impaired right ventricular function, while the RAP+LBBB group exhibits more severe decreased compliance and fibrosis in left ventricle.

## 58 THE IMPACT OF STATIN THERAPY ON ATRIAL MYOPATHY IN ATRIOVENTRICULAR BLOCK PATIENTS WITH RV PACING: FROM BENCH TO CLINICAL STUDY

### 58.1 **YU‐SHENG LIN** ^1^, WAN‐CHUN HO^1^, MIEN‐CHENG CHEN^2^

#### 58.1.1 ^1^Chang Gang Memorial hospital, Chiayi, Taiwan,^2^Chang Gang Memorial hospital, Kaohsiung, Taiwan

**Introduction:** Left atrial myopathy often precedes atrial fibrillation (AF) in atrioventricular block (AVB) patients with right ventricular (RV) pacing. This study examined statin therapy's potential to prevent myopathy progression and AF.

**Methods:** A clinical cohort from Chang Gung Memorial Hospital was assessed using Inverse Probability of Treatment to balance patients with or without statin therapy. The outcomes assessed included the incidence of AF and left atrial (LA) size. Complementing the clinical data, an animal study involving groups of 6 pigs each—sham control, RV pacing, and RV pacing with atorvastatin treatment—was conducted to investigate changes in left atrial function and to assess myocardial fibrosis and collagen deposition.

**Results:** Clinically, 2338 eligible patients were divided into 304 in the statin group and 2034 in the non‐statin group. After IPTW adjustment, results showed that statin therapy did not lead to a statistically significant reduction in AF incidence (10.5% vs. 10.6%; HR: 1.11 [0.83‐1.48]) in a 5‐year follow‐up period. Similarly, no discernible benefit of statins was observed in terms of maintaining LA size across the examined follow‐up intervals. Animal model investigations paralleled these findings, with no substantial difference in LA size (RV pacing vs. RV‐atorvastatin: 28.5±3.3 vs. 33.2±6.6 cm^3^) or ejection fraction (RV pacing vs. RV‐atorvastatin: 54.4±17.3 vs. 50.1±15.5%) noted among the RV pacing and statin‐treated RV pacing groups. Furthermore, statin treatment did not significantly attenuate myocardial fibrosis or collagen deposition compared to non‐treated RV pacing animals, suggesting a lack of therapeutic benefit in the structural preservation of atrial tissue.

**Conclusions:** The results indicated that statin therapy does not confer significant protective effects against left atrial myopathy, nor in preventing the development of AF in the context of RV pacing‐induced LV dyssynchrony. These outcomes highlight the necessity for alternative therapeutic strategies to address atrial remodeling in AVB patients undergoing pacemaker implantation.

## 59 PULMONARY VEIN ISOLATION PLUS FOCI ABLATION GUIDED BY LOW‐DOSE ISOPROTERENOL &LT ADENOSINE TRIPHOSPHATE FOR PAF PATIENTS

### 59.1 **HUIYI LIU**, YUMEI XUE

#### 59.1.1 Guangdong provincial people' hospital, Guangzhou, China

**Introduction:** The recurrence of atrial arrhythmia after ablation mostly associated with pulmonary vein reconnected &lt non‐PV foci for patients with paroxysmal atrial fibrillation(PAF). This study was to estimate whether low‐dose isoproterenol (ISP) &lt adenosine triphosphate (ATP) could uncover the triggers of PAF in the absence of drug‐related adverse effects &lt increase the success rate of single procedure.

**Methods:** A total of 164 consecutive patients with PAF who underwent radiofrequency ablation in Guangdong provincial people's hospital were enrolled. 114 patients in control group did not underwent drug challenge. The remaining 50 patients accepted ISP &lt ATP challenge during procedure. ISP was infused by a rate of 2μg/min or 4μg/min from the beginning of procedure until atrial arrhythmia was induced or PVI was complicated. ATP(30 or 40mg) was injected to uncover dormant PV conduction &lt non‐PV foci. All patients accepted PVI, whether to perform empirical ablation was up to the operators.

**Results:** Atrial arrhythmia was induced in 40 patients by ISP or/&lt ATP. Clear triggers were found in only 35 patients (group2) &lt the trigger focus was not clear in 5 patients (group3). Any arrhythmia did not occur in the remaining ten patients during the procedure (group1). The mean ISP infusion time was 21.38±11.38 minutes. A total of 54 atrial arrhythmias were induced, which included 35 AF, 8 AFL, 4 AT, &lt 8 PAC. 80.00% of foci originated from PVs. The remaining triggers were distributed in LA posterior wall(5.00%), MI(5.00%), SVC(2.50%), LA anterior wall(2.50%), &lt ligament of Marshall(LOM, 2.50%). After 290.72±98.31 &lt 347.82±63.89 days of follow‐up, the recurrence rate of atrial arrhythmia in drug challenge group was significantly decreased than control group(10.00% vs 23.68%, *P*=0.042). Subgroup analysis found that compared with group3, the survival rate of atrial arrhythmia also decreased in group1 &lt group2(*P* =0.005).

**Conclusions:** Low‐dose ISP &lt ATP could uncover the foci of PAF. PVI plus foci ablation guided by these drugs could reduce the recurrence rate of atrial arrhythmia in PAF patients underwent first ablation.

## 60 HIGH‐LEVEL SPINAL CORD STIMULATION ELICITS PARASYMPATHETIC REMODELING IN A RAT MODEL OF CONGESTIVE HEART FAILURE

### 60.1 **SHIN HUEI LIU** ^1^, LI‐WEI LO^1^, YU‐HUI CHOU^1^, WEI‐LUN LIN^1^, TING‐AN LEE^1^, CHING‐WEN CHANG^2^, YOU‐YIN CHEN^2^, SHIH‐ANN CHEN^1^

#### 60.1.1 ^1^Taipei Veterans General Hospital, Taipei City, Taiwan,^2^National Yang Ming Chiao Tung University, Taipei City, Taiwan

**Introduction:** Spinal cord stimulation (SCS) induces autonomic nervous system (ANS) modifications and mitigates arrhythmias in diseased myocardial substrates. Mechanisms within the central ANS that impact cardiac heart rate variability (HRV) remained unclear. We aimed to explore the alterations in HRV resulting from various SCS protocols at the T‐spines.

**Methods:** Sprague‐Dawley rats were randomly assigned to sham (n=4) and congestive heart failure (CHF, n=4) groups. The CHF model was induced by left coronary artery ligation. The SCS was performed by exposing the epidural space and inserting a custom‐made probe. All rats underwent SCS at T‐spine levels 2‐3 and 3‐4 using protocols of 50 Hz/225μs/200μA and 50 Hz/225μs/300μA. The HRV parameters were investigated.

**Results:** Figure 1 revealed significant differences in RR intervals between SCS‐On and SCS‐Off in the sham group under 50 Hz/225μs/200μA and 50 Hz/225μs/300μA. protocols The CHF groups exhibited a discernible trend of RR interval changes between SCS‐On and SCS‐Off under 50 Hz/225μs/200μA and 50 Hz/225μs/300μA (Figure 1). Across all T‐spine locations, the LF values in the CHF‐On groups under SCS at 200μA and 300μA were significantly higher than those in the Sham‐On, Sham‐Off, and CHF‐Off groups under the respective SCS protocols (Figure 2B). The HF values in the CHF‐Off groups under SCS at 200μA and 300μA were significantly higher than those in the Sham‐On, Sham‐Off, and CHF‐Off groups under the corresponding SCS protocols (Figure 2C). The LF/HF in the CHF‐On groups under SCS at 200μA and 300μA was significantly higher than those in the Sham‐On, Sham‐Off, and CHF‐Off groups under the respective SCS protocols (Figure 2D).

**Conclusions:** High‐level SCS at T‐spine levels indicated sympathetic hyperactivity in CHF rats, accompanied by sustained elevated parasympathetic expression following the termination of SCS. The observed SCS‐induced parasympathetic remodeling suggests its potential therapeutic efficacy as an adjunct to standard CHF treatment.

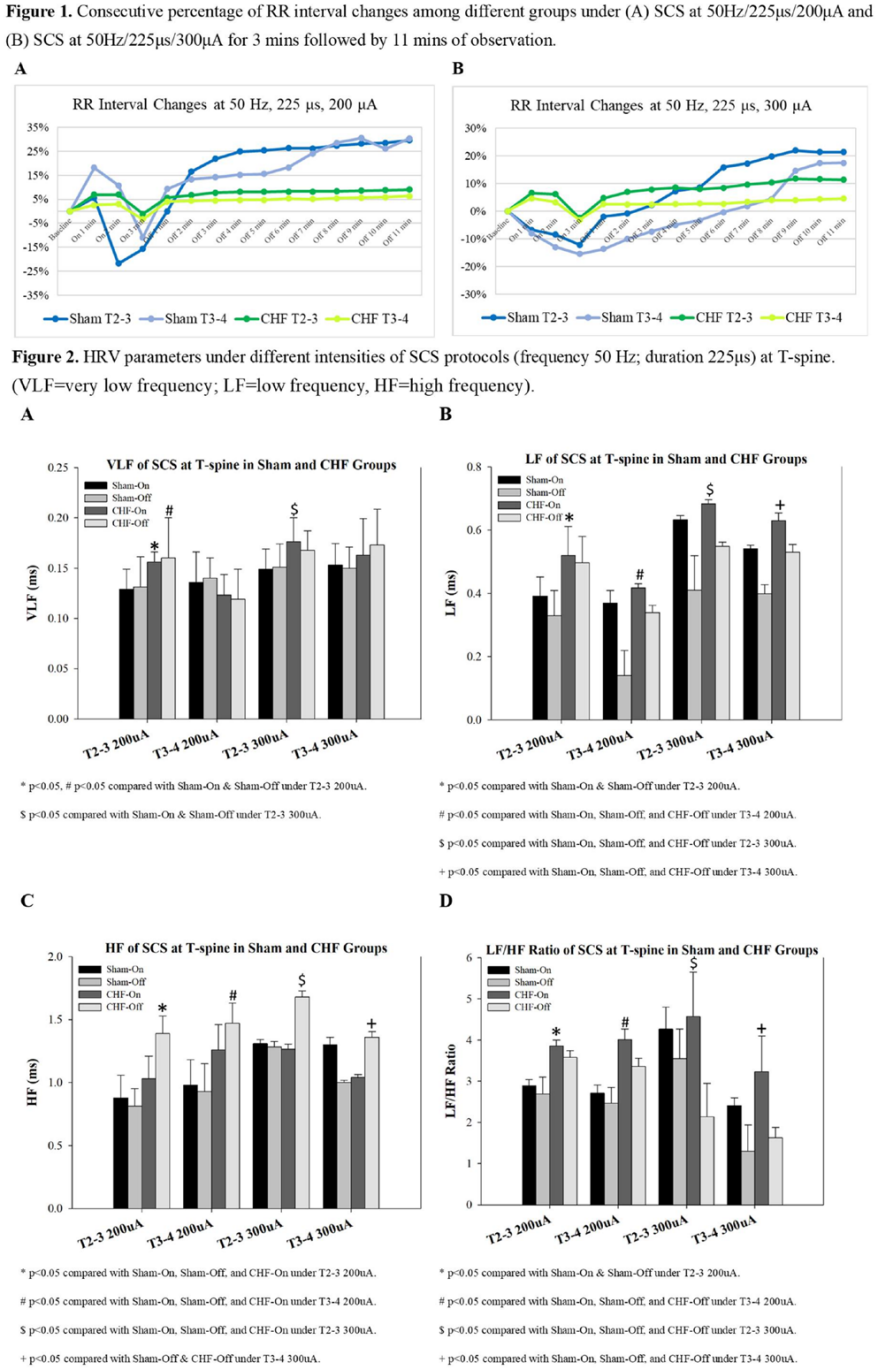

Chair

**T. Liu**;

Tianjin Institute of Cardiology, Second Hospital of Tianjin Medical University, China

## 61 EFFECTS OF RIVAROXABAN ON PROGNOSIS IN PATIENTS WITH ACUTE MYOCARDIAL INFARCTION AND PREEXISTING ATRIAL FIBRILLATION

### 61.1 **TONG LIU**, YING LIU, YI ZHENG, GARY TSE, KANGYIN CHEN, GUANGPING LI

#### 61.1.1 Tianjin Institute of Cardiology, Second Hospital of Tianjin Medical University, Tianjn, China

**Introduction:** The study explored the effects of rivaroxaban on the prognosis of patients with AMI and preexisting AF treated with antiplatelet drugs.

**Methods:** Patients diagnosed with AMI and AF who were prescribed antiplatelet drugs in 72 secondary and tertiary hospitals in Tianjin, China, from August 2016 to June 2023 were enrolled. Propensity score matching was performed with a 1:1 ratio for rivaroxaban users versus non‐users was performed. The primary outcomes were any stroke, ischemic stroke, and hemorrhagic stroke. The secondary outcomes were all‐cause mortality, cardiovascular mortality, any bleeding and major bleeding. Multivariable Cox regression adjusting for age, gender, previous comorbidities, and concomitant medications was conducted. Sensitivity analysis was performed using multivariable competing risk analysis.

**Results:** A total of 1,819 patients were identified, which included 340 rivaroxaban users and 1,479 non‐users. Over a median follow‐up of 922 days (interquartile range, 425‐1525), 561 patients died and 631 patients suffered from stroke. Compared to non‐users, rivaroxaban users had a lower risk of any stroke (hazard ratio [HR], 0.71; 95% confidence interval [CI], 0.56‐0.90) and ischemic stroke (HR, 0.72; 95%CI, 0.57‐0.92). In the matched cohort (n=312 in each group), rivaroxaban users had a lower risk of any stroke (HR, 0.64; 95%CI, 0.49‐0.85) and ischemic stroke (HR, 0.67; 95%CI, 0.51‐0.90) with no significant difference in any bleeding or major bleeding. Multivariable competing risk model confirmed the significant association between rivaroxaban use and any stroke (HR, 0.67; 95%CI, 0.51‐0.89) and ischemic stroke (HR, 0.70; 95%CI, 0.53‐0.93). No significant association was found for rivaroxaban use and bleeding, major bleeding, all‐cause mortality, or cardiovascular mortality (Figure).

**Conclusions:** Concomitant rivaroxaban use is associated with lower risks of any stroke, especially ischemic stroke, with similar risks of any bleeding and major bleeding in patients with AMI and preexisting AF taking antiplatelet drugs.

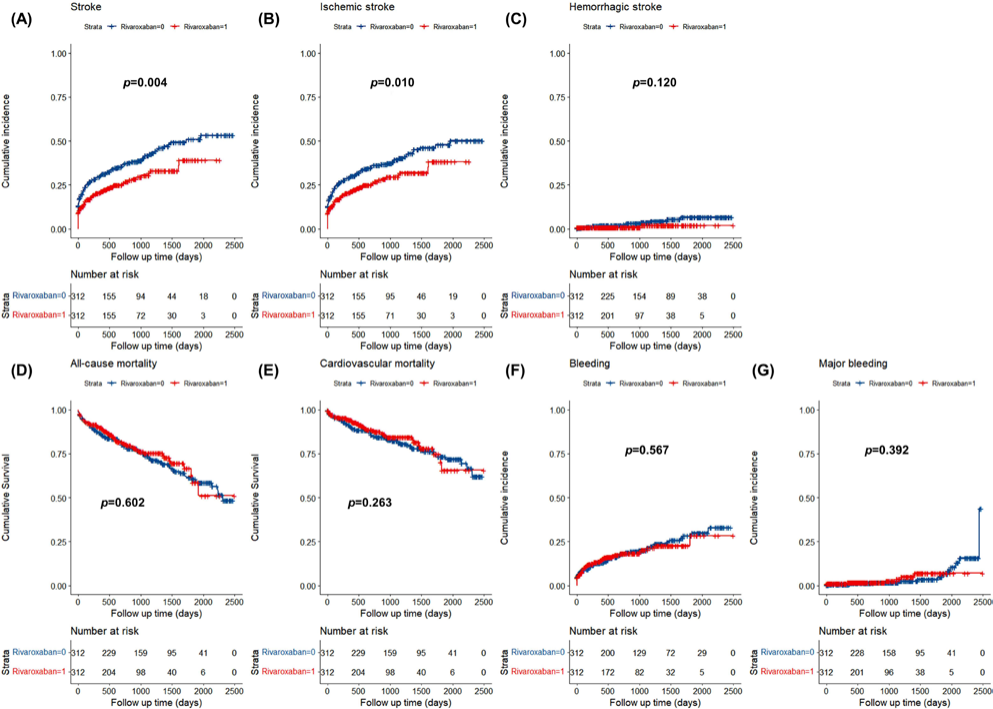

## 62 MULTIPOLAR PULSED FIELD VERSUS CONVENTIONAL THERMAL ENERGY ABLATION FOR PULMONARY VEIN ISOLATION: A SYSTEMATIC REVIEW AND META‐ANALYSIS

### 62.1 **XIAOHUA LIU**, XIAOFEI GAO, LIAN CHEN, YUFENG QIAN, YIGANG ZHONG, YIZHOU XU

#### 62.1.1 Hangzhou First People's Hospital, Hangzhou, China

**Introduction:** Atrial fibrillation (AF) is a common cardiac arrhythmia associated with significant morbidity and mortality. Catheter ablation using pulsed‐field (PF) energy seemed to exhibit promising efficacy and safety profile over conventional thermal energy (TE) ablation (TEA). However, limited knowledge is available on the differences between pulsed‐field ablation (PFA) and TEA for pulmonary vein isolation (PVI).

**Methods:** A systematic search of PubMed, EMBASE, the Cochrane Library, and ClinicalTrials.gov was performed to compare atrial arrhythmia (AA) recurrence and periprocedural adverse events between PFA and TEA for PVI. We analyzed data from 14 studies involving 5119 patients.

**Results:** The pooled analyses demonstrated a significantly reduced risk of AA recurrence (RR = 0.81, 95% CI, 0.71−0.93, P= 0.003, I^2^= 23%) and adverse events (RR: 0.49; 95% CI, 0.34‐0.69; P< 0.0001; I^2^= 22%) in PF‐PVI versus TE‐PVI. In sub‐analyses, PFA might achieve more efficacy in rhythm control when compared to cryoablation (RR: 0.77; 95% CI, 0.61‐0.97; P= 0.03; I^2^= 38%) rather than radiofrequency ablation. Patients with paroxysmal AF (RR: 0.80; 95% CI, 0.68‐0.94; P= 0.006; I^2^= 16%) possibly benefitted more rate of sinus rhythm maintenance from PF‐PVI than those with persistent AF. For adverse events, PFA seemed to significantly decrease the risk of damage to adjacent structures (RR: 0.11; 95% CI, 0.05‐0.27; P< 0.0001; I^2^= 0%) instead of non‐adjacent structures.

**Conclusions:** PFA appears to be more effective and safer than TEA for PVI. However, further studies are required to validate these findings.

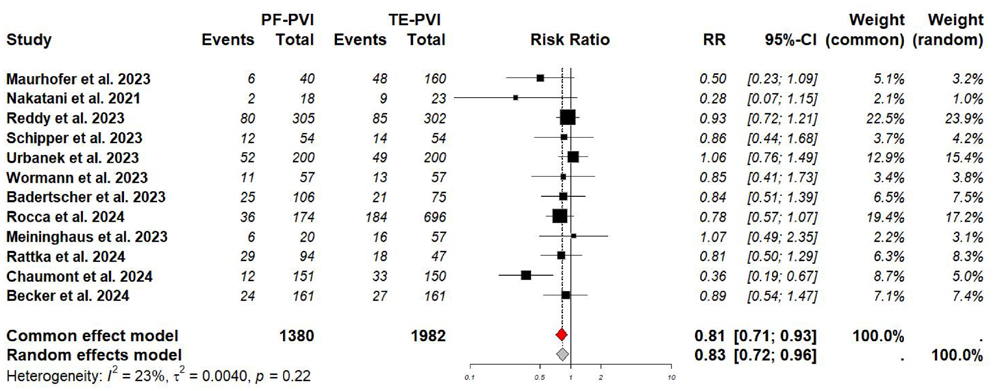

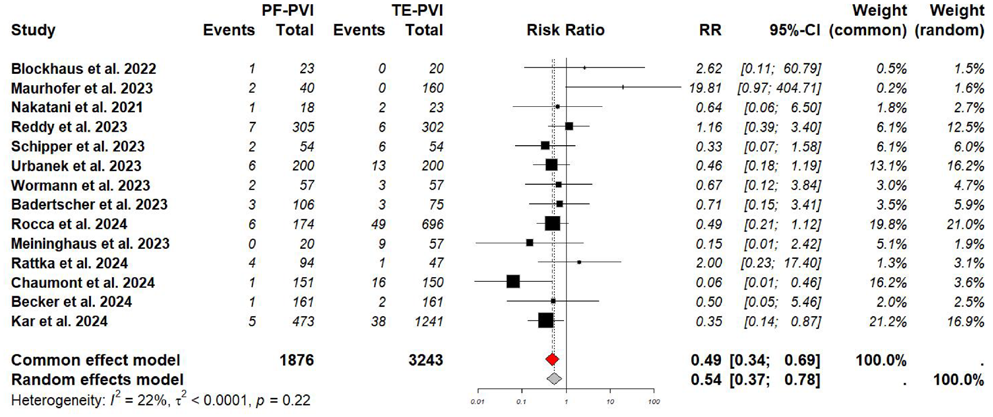

## 63 NKX2.5 VARIANT IN CHILDREN ‐ THE NEW SOUTH WALES EXPERIENCE

### 63.1 **WING KWAN WINKY LO** ^1^, CHRISTIAN TURNER^2^, CLAIRE LAWLEY^2,3^, JONATHAN SKINNER^2,3^, HIROKO ASAKAI^2^

#### 63.1.1 ^1^The University of New South Wales (UNSW), Faculty of Medicine, Sydney, Australia,^2^The Heart Centre for Children, Sydney Children's Hospitals Network, Sydney, Australia,^3^Faculty of Medicine and Health, The University of Sydney, Sydney, Australia

**Introduction:** NKX2.5 gene, a cardiac specific homeobox gene, is pivotal in cardiac development. Variants are associated with congenital heart disease (CHD), most commonly atrial septal defect (ASD), atrioventricular block (AVB). There are increasing reports of cardiomyopathy as well as sudden cardiac death (SCD) associated with NKX2.5 variants. SCD can occur despite pacemaker therapy for AVB, suggesting an increased risk of ventricular arrhythmias (VA).

**Methods:** A retrospective review of two families with NKX2.5 variants seen at two tertiary paediatric hospitals in Australia.

**Results: *Family 1:*** A 15‐years‐old boy was referred after Holter monitoring revealed variable AVB (first and second degree). He had ASD repair as an infant. There was a significant family history of CHD and SCD. His brother had ASD repair and was reviewed for syncope with an unremarkable exercise stress test but died suddenly 18 months later at age 18 years. Their father had repaired ASD, pacemaker for AVB, and had SCD in his 50s with documented VA. In light of this family history, genetic testing was performed in the two siblings, revealing a NKX 2.5 variant. The boy had a primary prevention ICD implanted with documented non‐sustained ventricular tachycardia (NSVT) during follow‐up. ***Family 2:** A* 10‐years‐old boy presented with ASD and complete AVB. His father had a stroke which led to the diagnosis of ASD, AF and nocturnal AVB. Genetic testing identified a missense NKX 2.5 variant in both. The boy had surgical ASD closure and ICD implantation. His older sister was subsequently diagnosed with an ASD and first degree AVB. Genetic testing identified the familial variant. She underwent device closure and transvenous ICD implantation. During follow‐up, both developed progressive AVB requiring pacing as well as NSVT.

**Conclusions:** Our experience highlight the expanded phenotypic spectrum of NKX2.5 variants beyond CHD and AVB. These cases underscore the importance of genetic testing in patients with CHD (ASD) and AVB, especially when there is a family history. An ICD should be considered particularly if there is a pacemaker indication for AVB given the increased risk of SCD.

## 64 ELECTROCARDIOGRAPHIC PROFILE OF ADULT PATIENTS WITH CORONAVIRUS DISEASE ADMITTED IN A TERTIARY HOSPITAL IN THE PHILIPPINES GIVEN REMDESIVIR

### 64.1 **MARIE KIRK PATRICH MARAMARA**, KAYE EUNICE LUSTESTICA, PAUL ANTHONY ALAD, ZANE OLIVER NELSON, BRYAN PAUL RAMIREZ, TAM ADRIAN AYA‐AY, NIGEL JERONIMO SANTOS, FELIX EDUARDO PUNZALAN

#### 64.1.1 PHILIPPINE GENERAL HOSPITAL, METRO MANILA, Philippines

**Introduction:** Severe Acute Respiratory Syndrome ‐ Coronavirus‐2 (SARS‐CoV‐2) was initially known to affect the respiratory system and has been reported to also involve the cardiovascular system leading to myocardial damage. Remdesivir is one of the approved treatments for the COVID‐19. The electrocardiographic (ECG) profile of adult patients with COVID‐19 given Remdesivir is unknown locally.

**Methods:** This was a retrospective descriptive study involving adult patients with COVID‐19 admitted in UP‐PGH given Remdesivir from June 2021 to June 2022. Demographic profiles and 12‐lead ECG done during the hospital admission were gathered. Descriptive statistics was used to summarize the clinical characteristics and the electrocardiographic findings of the patients.

**Results:** There were 412 confirmed COVID‐19 patients given Remdesivir (mean age 56 years old; female 52%) included in this study. The most common comorbidities were hypertension, diabetes mellitus, and stroke. Majority of the patients has severe (58%) to critical (22%) COVID‐19 infection. Most of the patients had sinus rhythm (94%), normal rate (72%) and normal axis (93%). The most common baseline ECG findings were non‐specific ST‐T wave changes (42%). Some patients had atrioventricular blocks (3.4%), bundle branch blocks (3.6%), prolonged QT interval (1.9%). Among those with repeat 12‐L ECG (136 patients) during admission, ECG changes observed were sinus bradycardia (6%), prolonged QT interval (4%) and both (1.5%).

**Conclusions:** Based on this retrospective review done locally in the Philippines,majority of patients were in sinus rhythm and normal axis.This study showed a low incidence of adverse ECG changes that would preclude administration of Remdesivir when indicated. These include sinus bradycardia and QT interval prolongation which did not require further interventions.

Chair

**A. Martin**;

Auckland City Hospital, Auckland, New Zealand

## 65 RELATIONSHIP BETWEEN TRANSSEPTAL PUNCTURE SITE LOCATION AND FIRST‐PASS PULMONARY VEIN ISOLATION SUCCESS RATE DURING RADIOFREQUENCY CATHETER ABLATION FOR ATRIAL FIBRILLATION

### 65.1 **KOHEI MATSUNAGA**, TADASHI HOSHIYAMA, YUTA TSURUSAKI, YUICHIRO TSURUTA, HITOSHI SUMI, SHOZO KANEKO, HISANORI KANAZAWA, KENICHI TSUJITA

#### 65.1.1 Kumamoto university hospital, Kumamoto, Japan

**Introduction:** Recently, radiofrequency catheter ablation (RFCA) for atrial fibrillation (AF) has become an important treatment strategy, especially for the patients who suffered heart failure induced by AF. During this procedure, residual conduction gap during pulmonary vein isolation (PVI) has shown to be independent risk factor for AF recurrence. Although various risk factor has proposed to be creating residual conduction gap, relationship between transseptal puncture area and residual conduction gap has not been clarified yet.

**Methods:** A total of 102 consecutive patients who had undergone their first RFCA for AF were included in this study. Transseptal area of each patients’ fossa ovalis were divided into four areas (supero‐anterior, supero‐posterior, infero‐anterior, and infero‐posterior) which were confirmed fast anatomical map in 3‐D mapping created using intracardiac echocardiography before PVI. Relationship between transseptal area among 4 groups and residual conduction gap following pulmonary vein isolation were analyzed.

**Results:** Among all 102 patients, residual conduction gap was observed in 32 patients. Although the ablation index was not significantly different between each transseptal groups (supero‐anterior 396.8±5.0, supero‐posterior 398.6±5.3, infero‐anterior 400.8±6.9, and infero‐posterior 401.5±5.4; p=0.233), residual conduction gap was significantly lowest at infero‐posterior group (13/61: 21%) than those of other groups (supero‐anterior 4/6:67%, supero‐posterior 5/9:56%, and infero‐anterior10/26:39% ;p<0.05). Actually, in infero‐posterior group, p‐vector which represent insufficient catheter contact was significantly observed lowest at infero‐posterior group(8.6% ;p<0.01).

**Conclusions:** The transseptal puncture site in PVI was an important factor to achieve first‐pass PVI. As a result, worsening heart failure accompanied AF recurrence might also decrease.

Chair

**M. McGuire**;

Royal Prince Alfred Hospital, Australia

## 66 OPTIMAL WOI FOR ACTIVATION MAPPING OF TYPICAL AFL

### 66.1 SUNG HO MOON

#### 66.1.1 ASAN MEDICAL CENTER, Seoul, Korea, Republic of

**Introduction:** Obtaining the activation map with 3D mapping system such as CARTO 3 or Ensite is important step for ablation of cavotricuspid isthmus (CTI) dependent counter clockwise atrial flutter (typical atrial flutter). During activation mapping, reference is usually set with coronary sinus electrode, and window of interest (WOI) is usally set as 95% coverage of tachycardia cycle length with reference in the middel of WOI. This method can easily confirm the re‐entrant circuit, but has limitation that early‐meet‐late line is not targeted to be at the critical isthmus. So we conducted this study to make a standard for setting the reference and WOI in typical atrial flutter to make early‐meet‐late line being at the critical isthmus

**Methods:** In single center (Asan medical center, Korea), consecutive 20 patients were enrolled in this study from January 2023 to July 2023. All patients were diagnosed as typical atrial flutter with activation mapping with 3D system (Carto3). To find the optimal reference and WOI to make early‐meet‐late line being at the critical isthmus, x‐ray image was used for setting the WOI early zone. Using Livewire Steerable electrophysiology catheter 7Fr duodecapolar catheter(Abbott, USA), distance from reference intra‐atrial electrogram to citical isthmus(CTI) was measured and used for WOI early zone.To evaluate the relation between critical isthmus(CTI) and surface electrocardiogram, distance from negative peak of inferior lead(lead II, lead III, and lead aVF) and positive peak of lead V1to CTI was analyzed

**Results:** Among 20 patients, mean age was 61.4 ± 8.68 years and male was predominant (65%). Atrial flutter cycle length was 252.25msec±42.13msec. Early zone was set as ‐33.75msec ±15.63msec earlier than reference electrode.(CS910) Distance from negative peak of inferior lead to CTI was ‐119.75msec±33msec with 47.58%±10.31% coverage of atrial flutter cycle length. Distance from positive peak of lead V1 to CTI was ‐173.25msec±33.65msec with 69.05%±9.61% coverate of atrial flutter cycle length.

**Conclusions:** In patients with typical atrial flutter, early‐meet‐late line can be made at critical isthmus by using distance from reference electrode to critical isthmus in WOI setting.

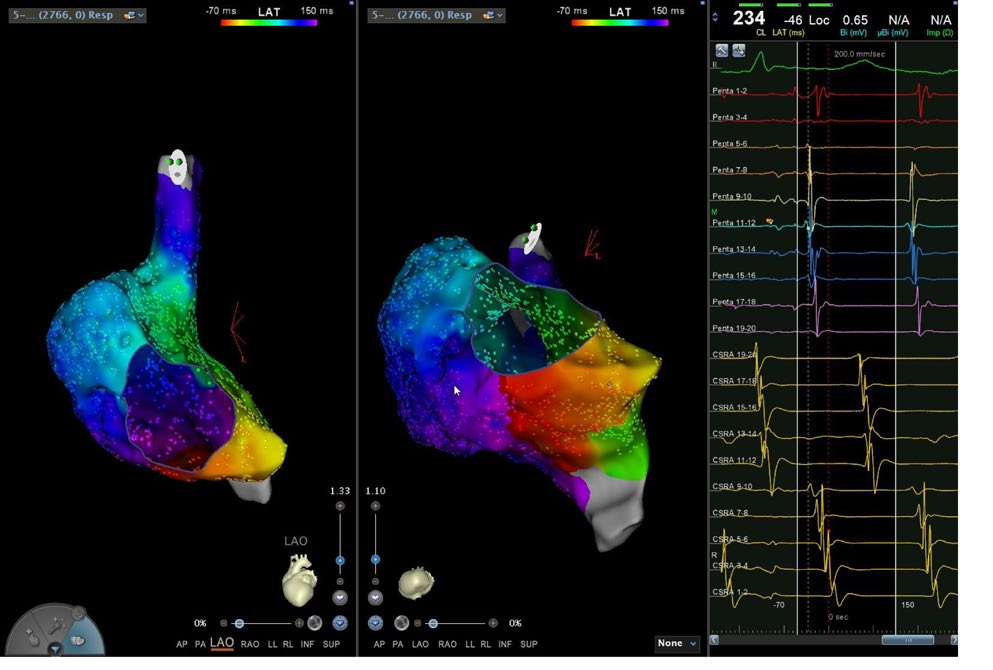

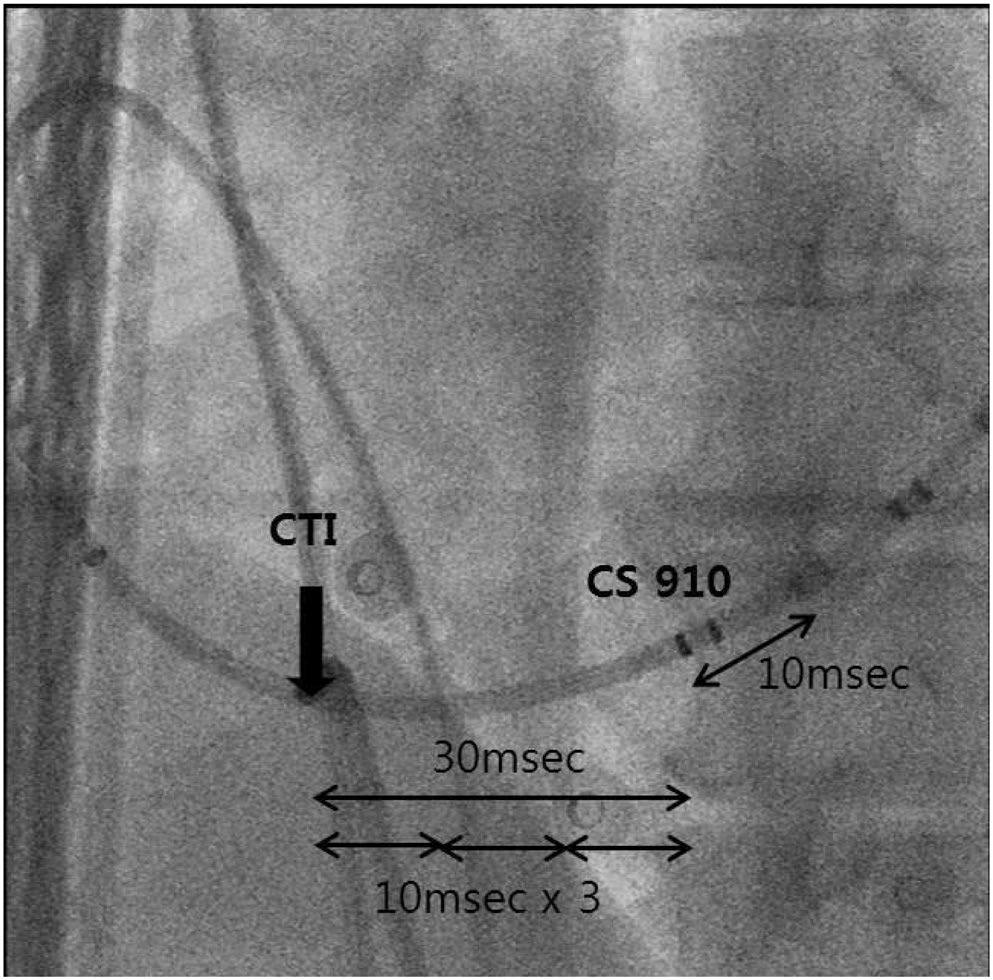

## 67 ROLE OF CONTINUOUS INTRAVENOUS LIDOCAINE IN PATIENT WITH CONGENITAL LONG QT SYNDROME AND 2:1 ATRIOVENTRICULAR BLOCK

### 67.1 **DEEBAJ NADEEM**, MUHAMMAD MOHSIN, FAISAL QADIR, REEMA QAYOOM

#### 67.1.1 National Institute of Cardiovascular Diseases, Karachi, Pakistan

**Introduction:** Congenital long QT syndrome (LQTS) is an inherited cardiac disorder characterized by abnormal cardiac repolarization, with prevalence of 1:2000. Rarely, it presents with 2:1 Atrioventricular (AV) block that usually manifest in fetus or neonates. Management include beta‐blockers, left cardiac sympathetic denervation, implantable cardioverter‐defibrillator, and avoidance of QT‐prolonging medications.

**Methods:** N/A

**Results:** A full‐term male newborn presented on 1^st^ day of life with 2:1 AV block and episodes of non‐sustained Torsade de pointes. An initial ECG of the infant depicted QTc of 681 ms and 2:1 AV block, with atrial rate 136 bpm and ventricular rate 68 bpm. Chest X ray, echocardiogram, physical examination, initial screening panel, complete blood counts and metabolic profile were normal. Parents had a normal baseline ECG. Family history was positive for sudden infant death in elder sister at 2^nd^ day of life. Mother had history of two episodes of syncope. Patient was started on oral propranolol with close heart rate monitoring with plan to put TPM in case of significant bradycardia or hemodynamic instability. However, it was later discontinued because of significant hypoglycemia. Continuous intravenous lidocaine infusion was initiated. After 40 hours, 1:1 AV conduction was restored with improvement in QTc intervals. Later, oral propranolol was re‐initiated with close blood sugar monitoring. After 72 hours, lidocaine infusion was switched with oral mexiletine and propranolol dose was increased. His corrected QT improved up to 443 ms by 7^th^ DOL. Gene panel came out negative for any LQTS associated mutation. He was discharged in stable condition. Later, exercise tolerance test of parents performed that showed QTc 486 milliseconds of mother during recovery. Mother has been started on beta blocker therapy and is doing well.

**Conclusions:** Continuous IV lidocaine infusion may represent a therapeutic option in the management of congenital LQTS and refractory arrhythmias, particularly in those with concomitant AV conduction abnormalities. Further studies are warranted to assess the long‐term efficacy and safety.

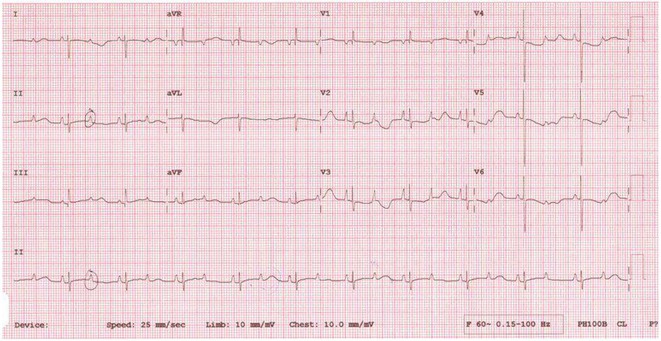

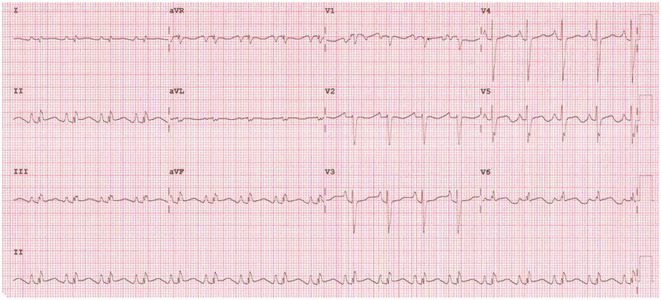

## 68 ARTIFICIAL INTELLIGENCE‐GUIDED PULSED‐FIELD ABLATION FOR REPEAT CATHETER ABLATION OF ATRIAL FIBRILLATION

### 68.1 **DEVI NAIR** ^1^, GANESH NAIR^2^, KIROLLOS GABRAH^2^, BRANDON DOTY^2^, JONATHAN CAIN^2^, ARUN MAHTANI^2^

#### 68.1.1 ^1^St. Bernards Medical Center & Arrhythmia Research Group, Jonesboro, AR,^2^Arrhythmia Research Group, Jonesboro, AR

**Introduction:** Pulsed‐field ablation (PFA) safety and efficiency for cardiac ablation have been demonstrated for pulmonary vein isolation (PVI), but its use to target additional substrate is still under investigation. We aimed to combine PVI and electrogram (EGM)‐based ablation strategies using PFA and a novel clinically validated artificial intelligence (AI)‐based software developed to identify spatio‐temporal dispersion during repeat procedures.

**Methods:** This study was a prospective, single‐center, non‐randomized study. Patients with recurrent atrial fibrillation (AF) despite previous catheter ablation were enrolled. Electroanatomical mapping was performed with CARTO (Biosense Webster) or Ensite X (Abbott) mapping systems and the AI‐based software (Volta AF‐Xplorer, Volta Medical), an expertise‐based AI tool trained to detect spatio‐temporal dispersion in multipolar intracardiac EGMs. After biatrial mapping, pulmonary veins were re‐isolated and spatio‐temporal ablation guided by Volta AF‐Xplorer was performed with the Farawave catheter (Boston Scientific).

**Results:** A total of 19 patients underwent a repeat procedure (37% female, 69.2±10.4 years, BMI 31.8±9.7 kg/m^2^, hypertension 69%, HF 42%, diabetes 47%, CHA_2_DS_2_‐VASc Score 3.6±1.3). Ten patients were in spontaneous AF (53%), 3 (16%) in atrial flutter at the outset of the procedure, while AF was induced in 6 patients (32%). Mean procedure and biatrial mapping time were 71.4±15.6 and 15.5±4.9 min, respectively. No fluoroscopy was used. PVI was achieved and spatio‐temporal dispersion was targeted for ablation in all 19 patients, with a median of 52 applications [50‐58]. No complications occurred. Sinus rhythm conversion by ablation was reached for 17 patients (89%) and 2 patients (11%) were cardioverted and non‐inducible.

**Conclusions:** Spatio‐temporal ablation guided by AI‐based software can easily be implemented to support PFA procedure. While further studies are needed to validate this approach, this workflow is promising, i.e. short procedure time, high termination rate and no complications for patients undergoing a repeat procedure for AF.

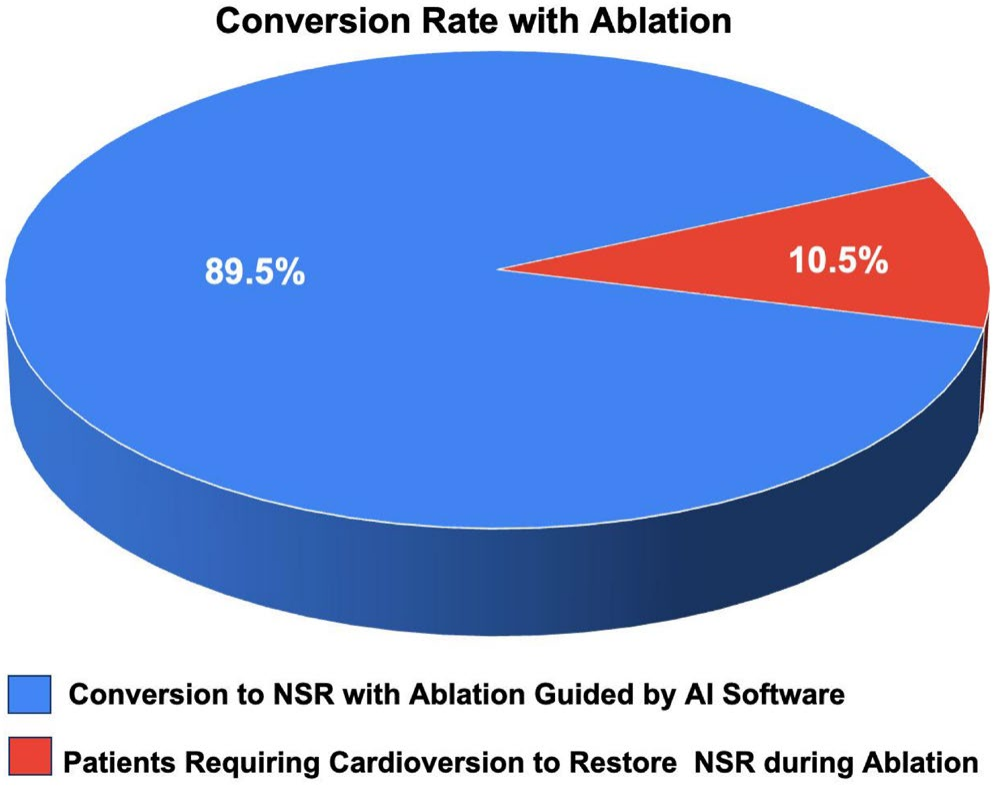

## 69 INCLUDE‐HEART PROJECT: INVESTIGATING NATIONAL CARDIOVASCULAR LEADERSHIP, UNDERSTANDING DIVERSITY AND EQUITY IN CARDIOVASCULAR MEDICINE CLINICAL TRIALS

### 69.1 **GANESH NAIR** ^1^, BRANDON DOTY^1^, ARUN MAHTANI^1^, KIROLLOS GABRAH^1^, DEVI NAIR^1,2^

#### 69.1.1 ^1^Arrhythmia Research Group, Jonesboro, AR,^2^St. Bernards Medical Center, Jonesboro, AR

**Introduction:** Gender, ethnic, & regional (GER) disparities among national principal investigators (NPI) of cardiovascular medicine clinical trials (CVMCT) may influence various aspects of the investigation's focus, methods, conduct, trial enrollment, & generalizability of the results. Such imbalances in clinical cardiology are acknowledged, yet diversity in CVMCT leadership and geographic disparities and their impact remain unexplored.

**Methods:** Data on CVMCT from Jan 2013 to Jan 2024 were extracted from Clinicaltrials.gov. Each study's NPI GER characteristics were examined including sponsor type, multiplicity of centers, geographic region, study size, gender, & ethnicity. The gender & ethnicity of NPI were determined based on their expressed identity in their professional profiles. US geography was categorized into five regions, & studies into five sizes (Figure 1). Multiplicity was binarized based on the number of enrolling centers. Sponsor types were industry, university, NIH, or other federal agencies. Time‐series analysis assessed trends toward GER parity in NPI over the last decade. CVMCT conducted outside the US or lacking identified NPI or locations were excluded.

**Results:** 24,848 CVMCT were analyzed. NPI were predominantly male (71%) & Caucasian (78%). Geographic distribution varied, with concentrations in some regions (28% region 1) & underrepresentation in others (12% region 4). Industry trials showed the most significant gender disparity (91% male). NIH‐sponsored trials exhibited consistent GER disparities (90% male, 86% Caucasian, 56% region 1). Other federal agencies demonstrated more equitable female representation (56%). Multicenter trials skewed male (82%) & Caucasian (82%). GER disparities persisted without significant improvement over the decade (P<0.05).

**Conclusions:** The unfavorable trends toward GER parity among NPI of CVMCT over the last decade demonstrate the urgent need for further investigation & the necessity for rigorous reexamination of efforts to foster diversity, inclusivity, & equity in CVMCT leadership.

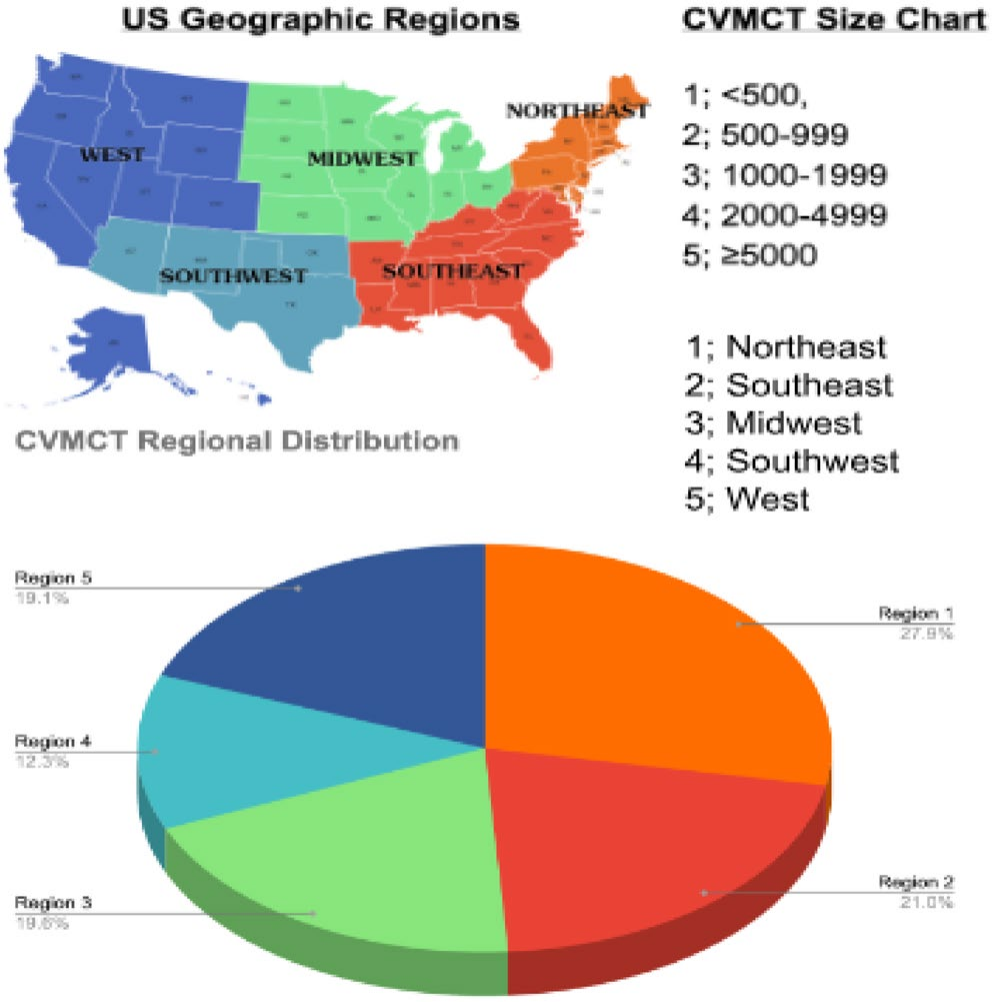

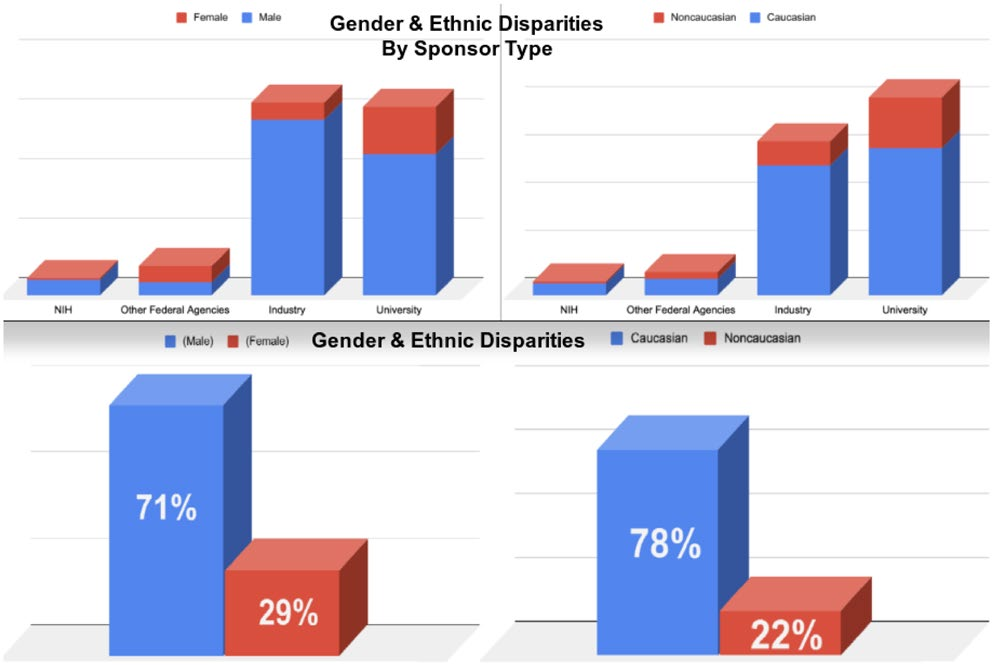

## 70 SERUM VASOACTIVE INTESTINAL PEPTIDE AND LOW‐VOLTAGE AREAS IN PATIENTS WITH ATRIAL FIBRILLATION

### 70.1 **KOTARO NISHINO** ^1^, TARO TEMMA^1^, KEI KAWAKAMI^1^, MASAHIRO KAWASAKI^1^, KINTARO SHIMANO^1^, SHOTA SAITO^1^, JIRO KOYA^1^, DAISHIRO TATSUTA^1^, HIROYUKI NATSUI^2^, TAKUYA KOIZUMI^3^, TAKAHIDE KADOSAKA^1^, TARO KOYA^1^, MOTOKI NAKAO^1^, MASAYA WATANABE^4^, TOSHIHISA ANZAI^1^

#### 70.1.1 ^1^Department of Cardiovascular Medicine, Faculty of Medicine and Graduate School of Medicine, Hokkaido University, Sapporo, Japan,^2^Department of Cardiovascular Medicine, Otaru Kyokai Hospital, Otaru, Japan,^3^Department of Cardiovascular Medicine, Hanaoka Seishu Memorial Hospital, Sapporo, Japan,^4^Department of Cardiovascular Medicine, Hokko Memorial Hospital, Sapporo, Japan

**Introduction:** Identifying low‐voltage areas (LVAs) within left atrium is crucial for predicting atrial fibrillation (AF) recurrence after catheter ablation. The impact of parasympathetic neurotransmitter vasoactive intestinal peptide (VIP) on atrial electrophysiological remodeling has been identified in animal experiments. Therefore, we hypothesized that serum VIP levels would be a valuable indicator of the presence of LVA.

**Methods:** An observational and prospective cross‐sectional study was conducted on patients undergoing catheter ablation treatment for AF between 2021 and 2023. Blood samples for VIP were collected before atrial septal puncture, and electroanatomic mapping (EAM) was performed using CARTO 3® (Biosense Webster, Diamond Bar, California, USA) before pulmonary vein isolation during the ablation procedure. VIP concentrations were measured using an ELISA kit. Patients were divided into two groups based on the presence of LVA (≥ 5% or < 5%) using EAM.

**Results:** Of the 108 patients eligible for analysis, 51 (47%) had LVA. VIP levels were significantly higher in patients with LVA than in those without (median/quartile range 335.1 [274.1 ‐ 456.5] pg/mL vs. 247.7 [196.1 ‐ 294.0] pg/mL, p< 0.001). Multivariate logistic regression analysis showed that VIP was an independent predictor of LVA (odds ratio 1.44, 95% confidence interval 1.13 ‐ 1.83, p< 0.001). Adding VIP level (≥288 pg/mL) to the existing score (age ≥65 years, female, and left atrial volume index ≥57 mL/m^2^) significantly improved the ability to identify LVA (area under the curve, 0.784: novel score vs. 0.707: existing score; p< 0.001).

**Conclusions:** We revealed that VIP levels are significantly higher in patients with LVA than in those without LVA. Furthermore, our analysis identified VIP as a reliable predictor of LVA, and a score that includes VIP to existing parameters has high discriminative power for LVA. Serum VIP would be effective in detecting LVA.

Chair

**M. O'Connor**

## 71 CLINICAL AND ELECTROPHYSIOLOGICAL CHARACTERISTICS OF FASCICULOVENTRICULAR PATHWAYS: A RETROSPECTIVE STUDY

### 71.1 **WEI SHENG JONATHAN ONG**, CHI KEONG CHING, KAH LENG HO, TIEN SIANG ERIC LIM, THUAN TEE DANIEL CHONG

#### 71.1.1 National Heart Centre Singapore, Singapore, Singapore

**Introduction:** Being a rare variant of pre‐excitation syndrome, fasciculoventricular pathways (FVPs) have historically been insufficiently described and reported. We aim to report one of the largest case series of FVP with a focus on their clinical and electrophysiological characteristics.

**Methods:** We retrospectively analyzed 35 patients who underwent electrophysiological study (EPS) and were diagnosed with FVP at our center between December 2013 to March 2024. Our study group consisted of pre‐military enlistment personnel referred for medical evaluation.

**Results:** Of the 35 patients, 34 (97%) were males. They had a mean age of 20.17 ± 0.48 years (range: 17‐23 years). 12 patients (34%) presented with palpitations, inclusive of the 6 patients (17%) with accompanying arrhythmia, while the rest were asymptomatic. All baseline electrocardiograms showed pre‐excitation and transitional zone was seen in leads V_2_‐V_4_. The mean PR interval was 115.74 ± 6.78 ms (range: 77‐160 ms) and the mean QRS interval was 97.74 ± 3.89 ms (range: 71‐119 ms). None of the patients had structural abnormalities of the heart noted on transthoracic echocardiogram. During the EPS procedures, a normal AH interval and a short HV interval were detected. The mean HV interval was 19.31 ± 3.01 ms (range: 0‐30 ms). All patients demonstrated AH prolongation after atrial pacing without a change in the degree of QRS pre‐excitation. The same degree of QRS pre‐excitation with a fixed HV interval was present during junctional beats as well. Retrograde conduction was concentric and decremental and there were no inducible tachycardias involving the FVPs. 4 patients (11%) FVPs exhibited intermittent conduction. 6 patients (17%) had concomitant arrhythmias, 1 had AV nodal reentrant tachycardia and 5 had AV reentrant tachycardia, all 6 were ablated successfully. All FVPs were followed up clinically with no arrhythmic events noted subsequently.

**Conclusions:** FVPs are rare innocent variants of pre‐excitation syndrome that do not participate in reciprocating tachycardia circuits. Accurate diagnosis is thus important so as to prevent unnecessary ablation with its accompanying risks.

Chair

**U. Pandurangi**

## 72 COMPARISON OF THE BURDEN OF VENTRICULAR ARRHYTHMIAS BEFORE AND AFTER LEFT VENTRICULAR ASSIST DEVICE IMPLANTATION

### 72.1 **YOUNG‐SUN PARK**, MIN‐SEOK KIM

#### 72.1.1 Asan medical center, University of Ulsan College of Medicine, Seoul, Korea, Republic of

**Introduction:** Ventricular arrhythmias (VAs) are frequently observed in patients who are awaiting or have already undergone left ventricular assist device (LVAD) implantation. We aimed to investigate whether LVAD implantation reduces the burden of VAs or if postoperative VAs are associated with increased mortality.

**Methods:** Between June 2015 and September 2023, we prospectively analyzed 92 patients who underwent LVAD implantation. The burden of VAs was defined as the total number of VAs or implantable cardioverter defibrillator (ICD) therapies including anti‐tachypacing (ATP) and appropriate shocks.

**Results:** In the 92 patients who successfully underwent LVAD implantation, the prevalence of VAs decreased following the procedure [37.0% (n=34) vs. 25% (n=23), p<0.01 for VAs before vs. after LVAD implantation]. However, there was no difference in the burden of VAs before and after LVAD implantation (4.81±34.03 vs. 2.80±13.07 events per patient‐month, p=0.96). The incidence of VAs, ATP, and appropriate shocks did not significantly decrease after LVAD implantation (2.74±18.71 vs. 1.52± 6.74, p=0.84; 1.21±8.15 vs. 1.08±6.32, p=0.53; and 1.00± 7.85 vs. 0.37±1.23, p=0.95, respectively). Logistic multivariable regression analysis revealed that preoperative VAs (hazard ratio 35.71 [6.20‐ 205.77]; p<0.01) were associated with postoperative VAs. However, there was no significant difference in survival regardless of the occurrence of postoperative VAs (p=0.91).

**Conclusions:** LVAD implantation did not reduce the burden of VAs. A history of preoperative VAs remained a risk factor for VAs following LVAD implantation. However, even if VAs occurred after LVAD implantation, the risk of mortality did not increase.

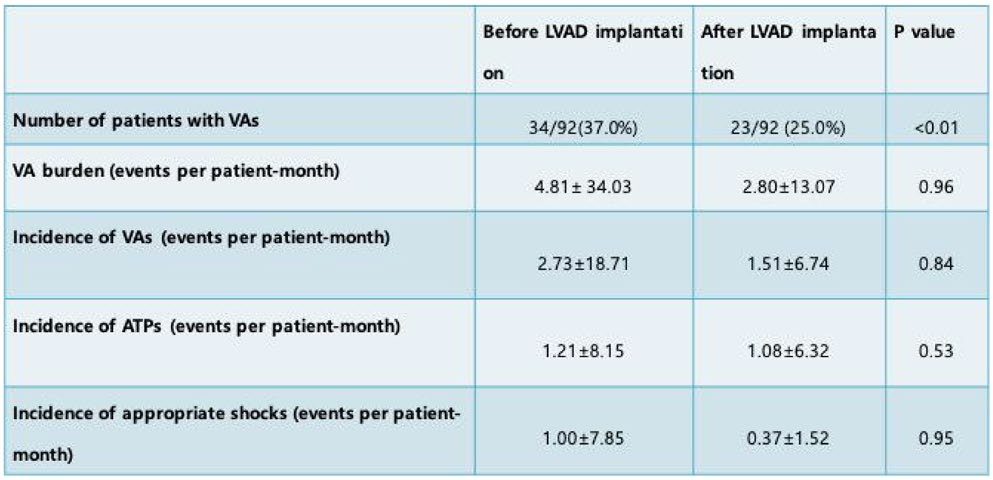

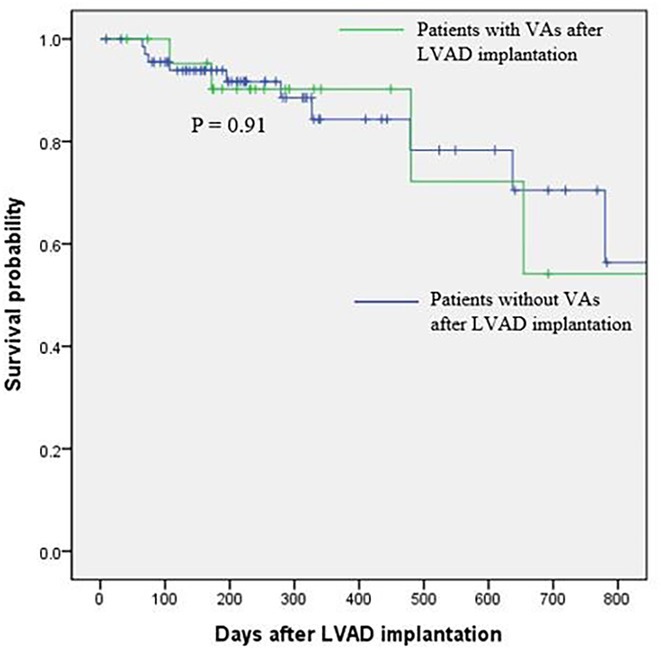

## 73 AN UNEXPECTED COMPLICATION OF PULMONARY VEIN ISOLATION ‐ CRYOABLATION

### 73.1 **NIKHILKUMAR PATEL**, PRAVEEN SREEKUMAR, ANILKUMAR RAJAPPAN

#### 73.1.1 Aster Medcity, Kochi, Kerala, India, Kochi, India

**Introduction:** AF is the most prevalent sustained cardiac arrhythmia. In patients with paroxysmal AF, Cryo‐PV isolation is an effective strategy with a shorter procedure time than conventional RFA. Most of the complications of cryo energy ablation are due to damage to structures close to the application site. Acute hemoptysis following PVI is rare complication and is most likely due to direct injury of the lung parenchyma and bronchial tree surrounding the PV, which can be attributed to lower temperature and deeper positioning during cryoablation. In most cases, it is self‐limiting. Endotracheal intubation is necessary for some patients to secure the airway.

**Methods:** N/A

**Results:** 73‐year‐old female with Type II DM presented with complaints of exertional breathlessness. Her ECG showed AF with CVR. Her CT CAG showed calcium in LMCA and RCA. She underwent CAG, which showed normal coronaries. During CAG, she developed AF with an FVR and acute pulmonary edema. AF was DC cardioverted, and she was managed medically. She was readmitted with AF with FVR and acute pulmonary edema. In view of refractory AF with recurrent HFpEF. She underwent PVI cryoablation with Medtronic Arctic Front cryoballoon. During the procedure, she developed hemoptysis. She was intubated to secure airway. For evaluation of hemoptysis, she underwent CTPA, which showed findings suggestive of aspiration with pulmonary haemorrhages. The next day, patient improved hemodynamically, and she was extubated. Thereafter, there was no recurrence of hemoptysis, and there was symptomatic improvement during follow‐up.

**Conclusions:** Hemoptysis during PVI cryoablation is a rare but life‐threatening complication. Important mechanisms for hemoptysis are direct injury of the lung parenchyma and bronchial tree surrounding the PV, pulmonary infarction and direct injury to PV during catheter manipulation. Avoiding deeper and non‐coaxial alignment of the cryoballoon to the PV axis can prevent hemoptysis. Early termination of ablation in the event of extremely cold cryoballoon temperatures can minimize the incidence of collateral injury. In most instances, it is self‐limiting, and symptomatic treatment is sufficient to tide over a crisis.

## 74 "RAP": BREAKING THE DEADLY CIRCUIT

### 74.1 **KUNARAJ PERUMALU** ^1^, SARAVANAN KRISHINAN^2^, MOHAMED JAHANGIR ABDUL WAHAB^1^, NG CHAI SHIN^1^, OOI TZE CHING^1^

#### 74.1.1 ^1^Hospital Pulau Pinang, Penang, Malaysia,^2^Hospital Sultanah Bahiyah, Alor Setar, Malaysia

**Introduction:** Scar‐related ventricular tachycardia (VT) is reentry caused by a combination of the activation and repolarization of the arrhythmogenic substrate. Scar VT ablation primarily guided by targeting RAP observed in mapping during baseline rhythm is associated with critical location of VT.

**Methods:** N/A

**Results:** A 59‐year‐old gentlemen with background history of diabetes mellitus, ischemic dilated cardiomyopathy and heart failure with reduced ejection fraction presented with recurrent palpitations in May 2024. He had history of anterior myocardial infarction in Year 2015 and had PCI done to LAD on same setting. His LVEF was 15% with ECG features of LBBB. Two years later patient's functional classification became NYHA III with recurrent heart failure symptoms. His repeat COROS in December 2017 showed patent proximal LAD stent. Despite optimal medical therapy patient had recurrent heart failure symptoms. CRT‐D was implanted in December 2017. He was admitted for multiple episodes of appropriate ICD shocks for VT. Device interrogation showed VT episode at a rate of 160/min not terminated by ATP and progressing to VF at a rate of 290/min terminated by 25J shock. An EPS and VT ablation was done on the same setting with further optimization of all 4 pillars of heart failure medication therapy. Baseline EP study yield short runs of VT. Left ventricle was mapped with RV pacing from device, double potential and fragmented signals were present at anteroseptal of LV basal which was at inferior LVOT. Split potential was consistently present at anteroseptal LV basal and mid‐basal. Rotational activation pattern was discovered at low voltage area. VT was induced during mapping and hence ablation done at anteroseptal LV basal. Homogenized the low voltage area and split potential eliminated at power of 40W. LV lateral and apical region were relatively healthy. Post VT ablation no PVC's or further VT were able to be induced.

**Conclusions:** The critical zone of VT reflects an area called RAP with slow conduction during sinus rhythm. The RAP facilitates in targeting areas specific for reentry. Identification of critical VT isthmus zones will be crucial with paced rhythm and pacing at sites near to the scar for successful ablation of scar‐related VT.

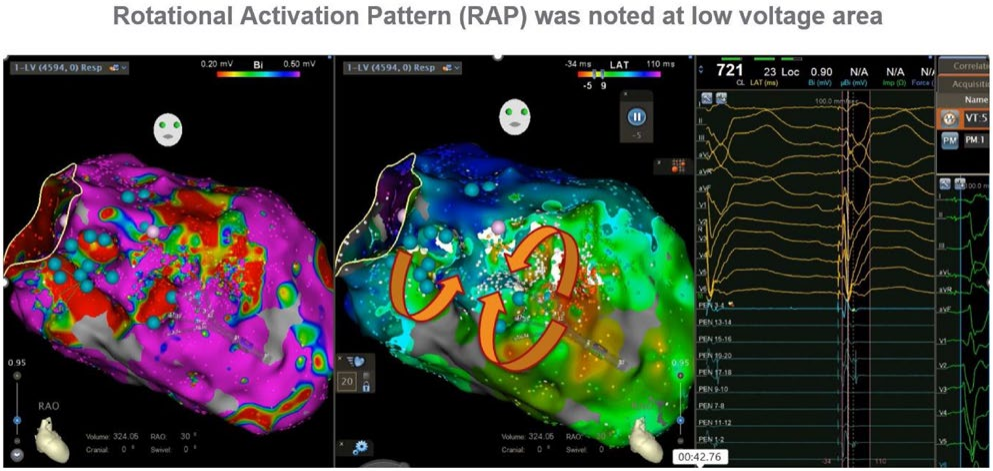

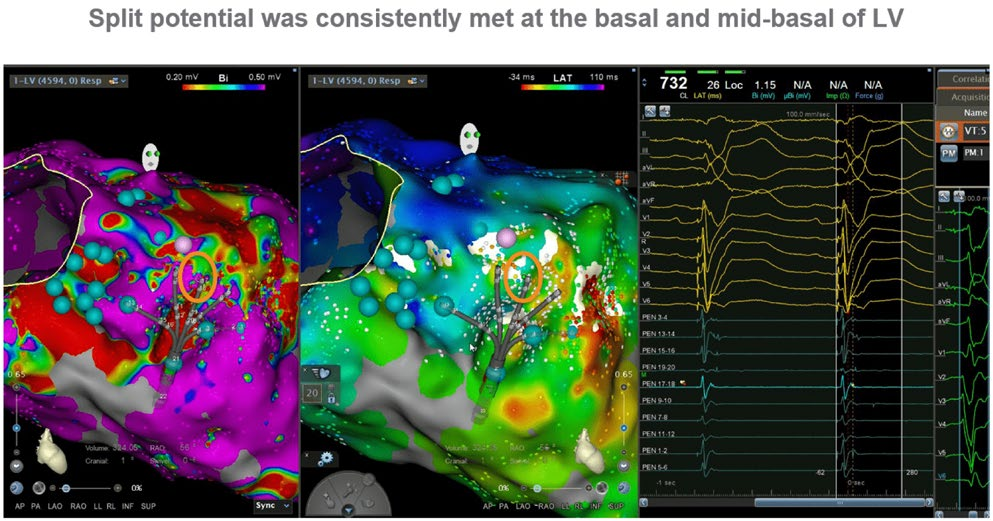

## 75 HYBRID TOTALLY THORACOSCOPIC MAZE FOR LONE PERSISTENT ATRIAL FIBRILLATION: EARLY OUTCOME ANALYSIS

### 75.1 ADRIAN PICK

#### 75.1.1 Victorian heart hospital, ARMADALE, Australia

**Introduction:** Hybrid epicardial and endocardial therapy has evolved as a breakthrough approach in the management of lone persistent atrial fibrillation(AF). Randomised trial results now report significantly improved outcomes, and the technique approaches mainstream integration into the guidelines for preferred management.

**Methods:** We present an analysis of 78 consecutive patients who underwent totally thoracocopic maze (TTMAZE) procedure from November 2017 to September 2023 by a single surgeon. Twenty patients underwent electrophysiologic three dimensional (3D) staged mapping. Preoperative workup included pulmonary function testing, echocardiography, and structural heart computed tomography to define the pulmonary anatomy and to exclude left atrial thrombus.

**Results:** The median age was 61+/‐ 7 years with 77% males and an average weight of 97 kg. Sixty seven patients were in persistent or long‐standing persistent AF. 12.8% had previous Transient Ischemic attack (TIA) and 2.6% had prior extracranial embolic episodes. Comorbidities included hypertension (37%), obstructive sleep apnoea (5%), smoking (19%) and coronary artery disease (1.2%). The average required hospital stay was 2.8 days. TIA was observed in 1.3%. Two patients received permanent pacemaker and two direct cardioversion as inpatients.The mean time to remapping was 5.5 months and within this cohort 90% of right superior veins and floor lines and 95% of left sided veins and roof lines were electrically isolated.Patients were followed for a mean of 3.5 years by which stage anticoagulation (AC) had been ceased in 46% and antiarrhythmics (AA) ceased in 65%. 88% patients reported improved symptom relief and quality of life, no patient reported deterioration. Three patients (3.8%) underwent subsequent additional epicardial ablation each of whom was in sinus rhythm at most recent follow up. The freedom from AF at most recent follow up was 87.5%.

**Conclusions:** This series of hybrid TTMAZE shows excellent outcomes with respect to lesion integrity, symptom relief, cessation of AA and AC's and safety. All patients will be followed long term.

## 76 LEFT ATRIAL POSTERIOR WALL ISOLATION IN ADDITION TO PULMONARY VEIN ISOLATION USING A PENTASPLINE CATHETER IN PULSED‐FIELD ABLATION FOR ATRIAL FIBRILLATION ‐ A SYSTEMATIC REVIEW AND META‐ANALYSIS

### 76.1 **RAYMOND PRANATA**, WILLIAM KAMARULLAH, GIKY KARWIKY, CHAERUL ACHMAD, MOHAMMAD IQBAL

#### 76.1.1 Department of Cardiology and Vascular Medicine, Faculty of Medicine, Universitas Padjadjaran, Hasan Sadikin General Hospital, Bandung, Indonesia

**Introduction:** This meta‐analysis aimed to investigate the feasibility and effectiveness of left atrial posterior wall isolation (LAPWI) in addition to pulmonary vein isolation (PVI) using a pentaspline catheter in pulsed‐field ablation (PFA) for atrial fibrillation (AF)

**Methods:** Comprehensive literature search was conducted using PubMed, SCOPUS, Sciencedirect, and EuropePMC for studies reporting LAPWI+PVI using a pentaspline catheter in PFA ablation for AF. The primary outcome was atrial tachyarrhythmia (ATa) recurrence, defined as AF/atrial flutter/atrial tachycardia after blanking period.

**Results:** There were 882 patients from 7 studies. The success rate of LAPWI was 100% using mean/median of 16‐20 added PFA applications with no reported acute LAPW reconnection and esophageal complications. In mean follow‐up of 240±91 days, ATa recurrence was 21% (95%CI 13‐29%; I^2^: 84.8%) in the LAPWI+PVI group. Meta‐regression analysis showed that age, LV ejection fraction, and repeat procedure did not significantly influence ATa recurrence (p>0.05). Each 1 mm increase in LA diameter, increases the chance of ATa recurrence by 6% (R^2^: 100%, p<0.001, I^2^: 0%). Meta‐analysis showed no difference in terms of ATa recurrence among LAPWI+PVI patients compared to those without LAPWI (OR 0.78 [95%CI 0.50, 1.21], p=0.27; I^2^: 0%, p=0.86). Procedure time (0.75 minutes [95%CI ‐11.28, 12.77), p=0.90; I^2^: 71%, p=0.03) and fluoroscopy time (0.72 minutes [95%CI ‐2.50, 1.07), p=0.43; I^2^: 23%, p=0.27) did not significantly differ.

**Conclusions:** LAPWI using a pentaspline catheter during PFA was feasible and did not prolong the procedure/fluoroscopy but did not reduce ATa recurrence. LAPWI may be considered during PFA, although the benefit is uncertain.

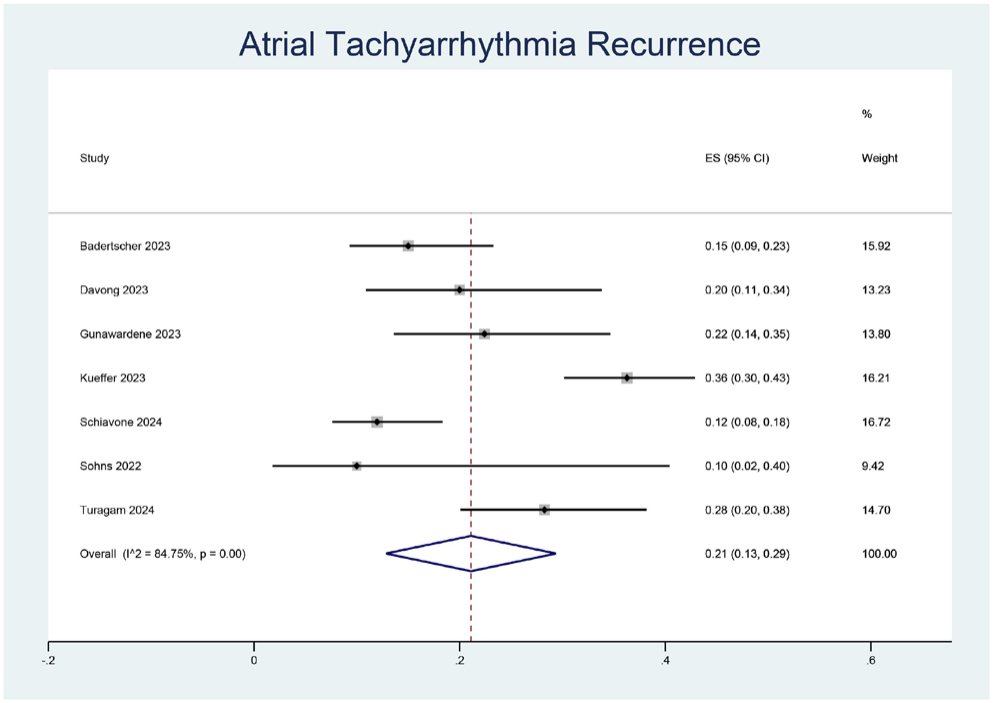

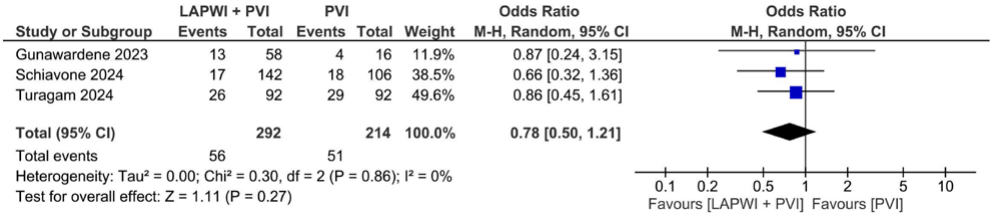

## 77 SAFE TARGETS FOR AVNRT ABLATION IN THE ERA OF HIGH DENSITY MAPPING: INSIGHT ON CONFLUENT AREAS AND SLOW PATHWAY SIGNAL FREQUENCY

### 77.1 **FABIO QUARTIERI** ^1^, ANTONELLA BATTISTA^1^, MATTEO IORI^1^, FRANCESCO MOSCATELLI^2^, NICOLA BOTTONI^1^

#### 77.1.1 ^1^Santa Maria Nuova Hospital, Reggio Emilia, Italy,^2^Abbott Medical Italy, Sesto San Giovanni, Italy

**Introduction:** Targeting slow conduction and electrogram fragmentation sites, Confluent Areas (CAs), in AVNRT ablation was recently proved to be safe and effective compared to conventional ablation. However, identification of CAs remains challenging. We aim to investigate the optimal way to provide a visual target for slow pathway (SL) ablation in the era of high density mapping (HDM) and omnipolar near field technology (OTNF).

**Methods:** We retrospectively investigated 10 AVNRT cases performed at our institution in 2023 enrolled in the prospective multicentric study (NCT055311903). The Koch's triangle (KT) was remapped with EnSite TurboMap exploiting the Advisor™ HD Grid and novel OTNF software not available at index procedure. Fractionation map and wave speed were inspected to find CAs targeted for ablation with a radiofrequency (RF) 4mm solid‐tip catheter 60W, 60°C. Signal peak frequency (PF) was acquired and overlapped to voltage maps [0.1‐1] mV to emphasize a visual target within the KT. The PF target was overlapped with CAs and compared to RF application to evaluate RF efficacy on target zone.

**Results:** 10 patients (age 55 [50‐60] years, 9 female) with inducible AVNRT had a sinus rhythm HD map of KT acquired in 6 [4‐10] min mapping time. The fractionation map revealed CAs with signals &gt; 2 fragmentation and wave speed within 0.6‐0.8 mm/ms significantly different from adjacent zones. PF mapping identified a target area of 11 [6‐16] mm^2^ with SP signals above 350Hz in the voltage map. We found CAs and PF emphasis to overlay for 30 [12‐60] % of the target area. RF delivery within the emphasized area (PF &gt; 350Hz and voltage &lt;1 mV) produced irritative junctional rhythm after 1 [1‐3] applications and after 10 [7 ‐ 25] sec of RF delivery. In addition, only 9% (7 over 80) of lesion placed outside the PF zone resulted in junctional rhythm.

**Conclusions:** We suggest that PF data combined with voltage map can easily provide a narrow visual target suitable for ablation evoking junctional irritative response from the first RF pulses. Further studies are needed to validate PF thresholds and prospectively validate the target as primary site for ablation.

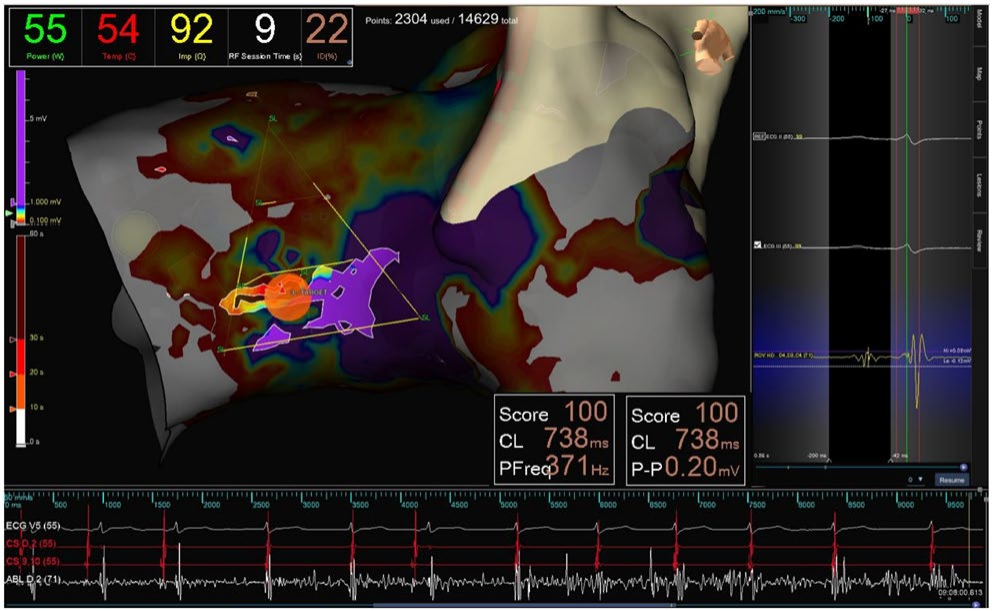

## 78 LEFT CORONARY ARTERY ‐ PULMONARY ARTERY FISTULA PRESENTING AS RECURRENT VENTRICULAR TACHYCARDIA IN ELDERLY PATIENT: A CASE REPORT

### 78.1 GEBRYEL SAERANG

#### 78.1.1 RS Saiful Anwar Malang, Malang, Indonesia

**Introduction:** Coronary artery fistulae (CAF) is a rare anomaly of the coronary anatomy, especially in the elderly, particularly in the elderly, they can occasionally induce functional myocardial ischemia and manifest as angina, dyspnea, and arrhythmia when hemodynamically significant. The clinical presentation of CAFs can vary from asymptomatic to sudden cardiac death. Cases of coronary artery fistula to the pulmonary artery with recurrent ventricular tachycardia (VT) are rare.

**Methods:** N/A

**Results:** An 81‐year‐old female patient presented to hospital with chest pain, palpitations and syncope, with an ECG showing monomorphic VT. The patient was cardioverted and given amiodarone with reversion to sinus rhythm with multifocal PVCs from LV origin. From physical examination, there was a continuous ‘machinery’ murmur at the left upper sternal border. During amiodarone treatment, the patient experienced recurrent VT which was terminated with lidocaine and at a rate of 60‐70 bpm. Coronary angiography showed a left main coronary fistula leading to the pulmonary artery through a large tortuous vessel. Coronary CT was performed for procedural planning and confirmed a coronary artery fistula leading through a large tortuous left main coronary artery to the pulmonary artery.

**Conclusions:** Coronary artery fistula in elderly patients are rare, especially when symptomatic. The mechanism of ventricular tachycardia in coronary artery to pulmonary artery fistulae is functional due to the 'coronary steal phenomenon'. Adults with haemodynamically significant coronary fistulae often present with symptoms and life‐threatening complications such as myocardial ischaemia or infarction, aneurysm formation and rupture, arrhythmias and congestive heart failure. An adequately performed CT scan with 3D reconstruction is invaluable in visualising the coronary anatomy for further treatment planning and correction of coronary artery fistulae. Closure is indicated for large or symptomatic fistula, either surgically or percutaneously, to prevent patient complications and sudden death.

## 79 PROGRAMMING RECOMMENDATIONS FOR A NOVEL INTEGRATED BIPOLAR DEFIBRILLATION LEAD: ANALYSIS FROM LEADR TRIAL

### 79.1 **PRASHANTHAN SANDERS** ^1^, PAMELA MASON^2^, BERT HANSKY^3^, PAOLO DE FILIPPO^4^, MAULLY SHAH^5^, PASCAL DEFAYE^6^, TRAVIS RICHARDSON^7^, THEODORE TAKATA^8^, BERNICE TSANG^9^, BAERBEL MAUS^10^, CHAD BOUNDS^11^, GEORGE CROSSLEY^7^

#### 79.1.1 ^1^University of Adelaide and Royal Adelaide Hospital, Adelaide, Australia,^2^University of Virginia Medical Center, Charlottesville, VA,^3^Städtische Kliniken, Bielefeld, Germany,^4^ASST Papa Giovanni XXIII, Bergamo, Italy,^5^The Children's Hospital, Philadelphia, PA,^6^Centre Hospitalier Universitaire de Grenoble, Site Nord, France,^7^Vanderbilt University Medical Center, Nashville, TN,^8^Texas Health Research & Education Institute, Austin, TX,^9^Southlake Regional Health Centre, Newmarket, ON, Canada,^10^Medtronic Bakken Research Center, Maastricht, Netherlands,^11^Medtronic, Inc, Minneapolis, MN

**Introduction:** The Lead EvaluAtion for Defibrillation and Reliability (LEADR) trial evaluated the small‐diameter, integrated bipolar OmniaSecure defibrillation lead. Additional analyses were conducted on P‐wave oversensing (PWOS) and mitigation robustness.

**Methods:** This pivotal trial enrolled patients with indications for de novo ICD/CRT‐D implant. The primary efficacy and safety endpoints were defibrillation success at implant and freedom from study lead‐related major complications at 6‐months, respectively. VF sensing and detection was evaluated at the least sensitive setting (1.2mV) via VF induction. Oversensing instances were reported by physicians and corroborated by sponsor scientists.

**Results:** In total, 643 patients were implanted (12.9±4.6‐month follow‐up). The lead exceeded prespecified primary safety and efficacy performance goals, with a 97.1% freedom from lead‐related major complications at 6 and 12 months and a 97.5% overall defibrillation efficacy at implant. There were zero lead fractures through follow‐up. Detection of induced VF at the least sensitive setting of 1.2 mV occurred in 119/122 (98%) patients. For 3 patients, VF undersensing occurred at 1.2mV and a subsequent test at a physician‐selected sensitivity confirmed detection at 0.3mV (2) and 0.9mV (1). PWOS was reported in 16 (2.5%) patients, all of which were resolved between implant and 3 months. There were zero inappropriate shocks associated with PWOS. Three resulted in system modification and 1 in symptomatic inhibition of pacing resolved by programming. The other 12 were resolved via programming RV sensitivity (11) and repositioning at implant (1). RV sensitivity was reprogrammed to 0.45mV (4), 0.6mV (5), 0.9mV (1) and 1.2mV (2) from 0.3mV nominal.

**Conclusions:** Chronic sensing performance of the OmniaSecure defibrillation lead has a low rate of PWOS events, mostly resolved by programming RV sensitivity. Furthermore, a high percentage of VF induction at implant (98%) showed appropriate detection at the least sensitive setting. These results show sensitivity to VF detection and indicate that programming changes are effective in PWOS resolution.

Chair

**R. Sandhu**;

University of Calgary, AB, Canada

## 80 IMPACT OF CATHETER ABLATION ON MITRAL REGURGITATION IN PATIENTS WITH ATRIAL FIBRILLATION AND LEFT VENTRICULAR SYSTOLIC DYSFUNCTION

### 80.1 **LOUISE SEGAN** ^1^, SANG JIN HAN^1^, MATTHEW WONG^1^, DANIEL MAHINI^1^, JAMES HARE^1^, JEREMY MOSKOVITCH^1^, DAVID CHIENG^1^, ROSE CROWLEY^1^, JEREMY WILLIAM^1^, KENNETH CHO^1^, ALEKSANDR VOSKOBOINIK^1^, HARIHARAN SUGUMAR^1^, LIANG‐HAN LING^1^, GEOFF LEE^2^, JOSEPH MORTON^2^, JONATHAN KALMAN^2^, SANDEEP PRABHU^1^, PETER KISTLER^1^

#### 80.1.1 ^1^Alfred Health, Melbourne, Australia,^2^Royal Melbourne Hospital, Melbourne, Australia

**Introduction:** Patients with AF and left ventricular systolic dysfunction(LVSD) have varying degrees of mitral regurgitation(MR).AF ablation is associated with significant recovery of LV systolic function however little is known regarding MR improvement.

**Methods:** We examined clinical characteristics,cardiac remodeling and ablation outcomes at 12 months among 275 patients with AF and LVSD according to baseline MR severity.

**Results:** 275 consecutive patients(age 60.6±10.2 years,17% female,NYHA class III) undergoing AF ablation were classified by baseline MR severity (trivial n=45,mild n=120,moderate or greater severity n=110). Baseline characteristics were comparable,however,those with at least moderate MR had lower LVEF(LVEF 26.8±10.0% vs mild:21.2±12.1% and trivial:37.6±8.9%,p<0.001) and significant TR (R^2^=0.488,p<0.001).LA dimensions did not significantly differ across MR groups(LAA X^2^ p=0.545,LAVI X^2^ p=0.574).Arrhythmia recurrence occurred in 45.8% and was comparable across subgroups(X^2^ p=0.093).Baseline MR severity did not influence arrhythmia recurrence (OR 1.31,95% CI 0.31‐5.43,p=0.713) nor LV recovery (OR 1.23,95% CI 0.83‐1.81,p=0.305). At 12 months,79.6% exhibited at least 1 grade of improvement in the degree of MR("MR responders”),with only 5.5% exhibiting moderate or greater MR at 12 months vs 40.0% at baseline(X^2^ p<0.001,figure 1a) accompanied by significant LA and LV reverse remodeling.Change in LV dimensions predicted "MR responders” (OR 0.89 95% CI 0.82‐0.96,p=0.003) with a greater reduction in LV size at 12 months in MR responders (‐5.9±5.7 vs non‐responders ‐2.0±5.0, p<0.001,figure 1b).Change in LA size did not influence MR improvement(OR 0.99, 95% CI 0.97‐1.03,p=0.742).Change in MR severity was inversely associated with AF burden at 12m(R^2^=‐0.275,p=0.004).

**Conclusions:** In patients with AF and LVSD undergoing AF ablation, there was a reduction in mitral regurgitation at 12 months corresponding to LV reverse remodeling,suggesting MR in this setting is largely driven by ventricular rather than atrial remodeling and reversibility is predicated on LV improvement.

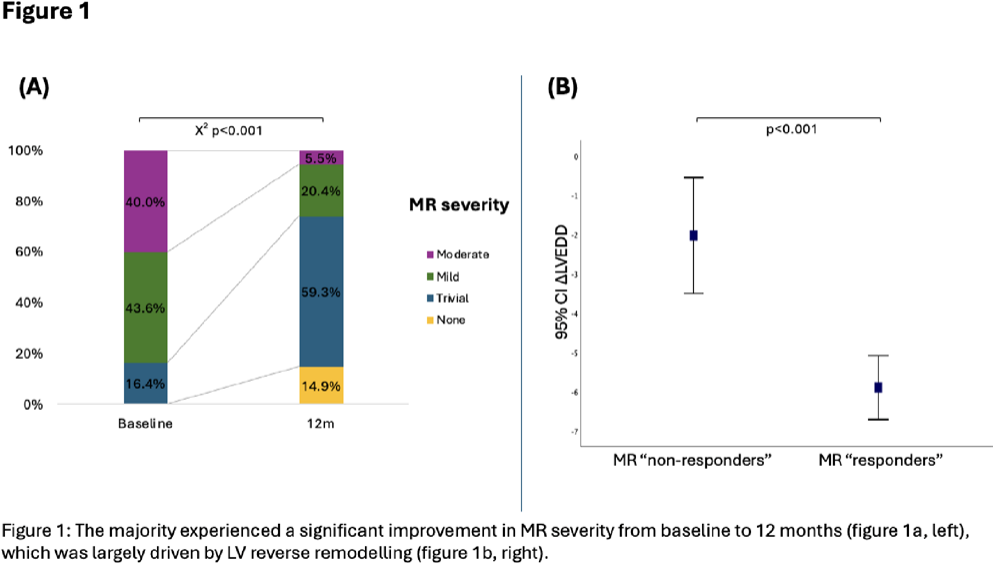

## 81 FIRST NON‐CLINICAL BENCH EVALUATION OF DIGITAL COMMUNICATION BENEFITS FOR SPATIOTEMPORAL DISPERSION MAPPING WITH THE OCTARAY CATHETER

### 81.1 **MICHAEL SHEHATA** ^1^, DAN SCHNEIDEWEND^2^

#### 81.1.1 ^1^Cedars Sinai Smidt Heart Institute, Los Angeles, CA,^2^GE Healthcare, Milwaukee, WI

**Introduction:** Spatio‐temporal dispersion (STD) ablation guided by artificial intelligence (AI)‐based software recently demonstrated robust performances during catheter ablation procedures of atrial fibrillation patients. The study aims to compare the impact of analog and digital acquisitions on the precision and accuracy of AI‐guided STD location.

**Methods:** We developed a non‐clinical bench model to map STD with either 10 or 24 bipoles (analog or digital communication respectively) using the Octaray (Biosense Webster) mapping catheter, an EP recording system (Cardiolab prototype, GE) and the AI‐guided STD system (Volta AF‐Xplorer prototype, Volta Medical). STD was captured on the central section of the catheter (analog output) or on every channel (digital output) (Figure, yellow circles). Signals were transmitted in real time through a dedicated protocol and Software API. Then, we modeled STD mapping in the atrium as a finite set of catheter moves. Each catheter location captures STD on a circle of radius R. Circles overlapped to ensure full surface covering, leading to a hexagonal tiling of side R (Figure). The number of catheter moves was estimated as the ratio between the total mapped area and the area of a single hexagon tile.

**Results:** When compared to analog communication, the implementation of STD detection by the digital communication mapping system‐AI software proved reliable. Increasing the number of available bipoles enabled better selection of neighbors used in STD AI computation: 10.1± 3 mm distance between neighbors in analog vs 6.8± 1.5 mm in digital. Bipole density was similar (2.7 bipoles/cm^2^ in analog vs 2.8 in digital), however, the area covered by each catheter move increased from 4.5 to 10.2 cm^2^ in digital. Using a perfect hexagonal tiling model, the number of catheter moves required to capture STD on a given surface was reduced by a factor of 2.25.

**Conclusions:** Digital communication provides a more suitable configuration for STD adjudication, enabling better selection of neighboring bipoles as well as fewer catheter moves.

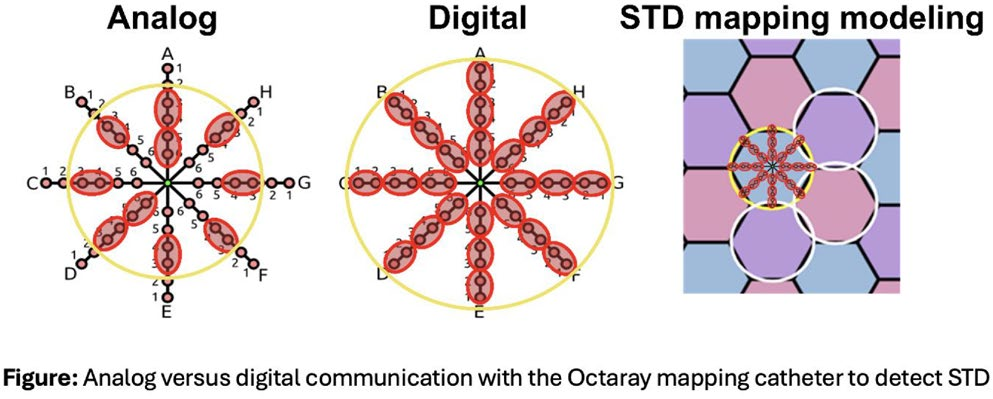

## 82 RISK FACTORS OF ATRIAL FIBRILLATION IN PATIENTS WITH WOLFF‐PARKINSON‐WHITE SYNDROME AND THE RESULT OF LONG‐TERM FOLLOW‐UP AFTER RADIOFREQUENCY CATHETER ABLATION

### 82.1 RAHUL SINGHAL

#### 82.1.1 Fortis Hospital Jaipur, Jaipur, India

**Introduction:** Atrial Fibrillation(AF) in Wolff‐Parkinson‐White(WPW) syndrome is a potential threat. Aim of the study to analyse the risk factors of AF in patients with WPW syndrome and to identify possible predictors of AF recurrence after successful ablation.

**Methods:** Total of 246 WPW syndrome underwent radiofrequency catheter ablation. Multiple factors such as sex, age, location and the number of accessory pathway, duration of AF history, shortest ECG RR interval during AF and size of left atrium were analyzed to define the risk factors of AF or the predictors for AF recurrence.

**Results:** Of total patients 46 had documented ECG of AF. Compared to patients without AF, patients with AF had more men (32/46 vs 14/200,P=0.001),older age (41.2vs32.6, P=0.001), higher incidence of multiple accessory pathways (12/46 vs 34/200,P=0.002). No difference noted between the locations of accessory pathways. In AF patients success rate of catheter ablation was 93.5% (43/46). Two patients had recurrence of WPW requiring the second ablation procedure. All patients were successfully followed up. 5/46 patients (10.85%) had documented ECG of AF. In 3 with recurrent AF, frequency and duration of AF and ventricular rate during AF were greatly improved compared with before ablation. The possible predictors for AF recurrence were examined. Patients with AF recurrence were more females (2/11 vs 1/35, P=0.02), older age (61.6 vs 43.2 years, P=0.05), more patients with associated cardiac diseases (3/15 vs 0/31, P=0. 001), larger LA size(40.46 vs 32.7mm, P0.003).

**Conclusions:** Males, elderly and multiple accessory pathways are the risk factors of AF in patients with WPW Syndrome. Radiofrequency catheter ablation of accessory pathway can cure WPW syndrome and AF spontaneously in most patients during a long period of follow‐up. Females, older age, associated cardiac diseases, longer duration of AF history and larger left atrial diameter found to be good predictors for the recurrence of AF.

## 83 PREDICTIVE & ASSOCIATIVE ROLE OF COMBINING SYSTEMIC INFLAMMATION RESPONSE INDEX WITH OBESITY INDICES IN HEART FAILURE: A STUDY BASED ON US NATIONAL DATA

### 83.1 CHUTAWAT KOOKANOK^1^, METHAVEE POOCHANASRI^1^, SETHAPONG LERTSAKULBUNLUE^1^, NARATHORN KULTHAMRONGSRI^2^, VARATHPAVEE BHURIVETH^1^, SORAWIS NGAOHIRUNPAT^3^, NICHA WAREESAWETSUWAN^4^, VITCHAPONG PRASITSUMRIT^4^, KAMONLUK RODSOM^4^, NISHA WANICHWECHARUNGRUANG^5^, VORAMOL ROCHANAROON^6^, TATCHAYA KANTHAJAN^7^, EKAMOL TANTISATTAMO^8^, **ADIVITCH SRIPUSANAPAN** ^9^

#### 83.1.1 ^1^Phramongkutklao College of Medicine, Bangkok, Thailand,^2^Department of Cardiovascular Medicine, Mayo Clinic, Phoenix, AZ,^3^Samutsakorn Hospital, Samutsakorn, Thailand,^4^Faculty of Medicine, Siriraj Hospital, Mahidol University, Bangkok, Thailand,^5^Central Chest Institute of Thailand, Bangkok, Thailand,^6^Rayong Hospital, Rayong, Thailand,^7^Faculty of Medicine,Srinakharinwirot University, Bangkok, Thailand,^8^Irvine Medical Center,Division of Nephrology, Hypertension and Kidney Transplantation, Department of Medicine, University of California Irvine School of Medicine, Orange, CA,^9^Chiang Mai University, Chiang Mai, Thailand

**Introduction:** Heart failure imposes a significant strain on healthcare systems worldwide. Considering the strong associations between inflammation and obesity with heart failure, we are exploring the use of available and cost‐effective indices, including the Systemic Inflammation Response Index (SIRI) along with body mass index (BMI) and waist‐to‐hip ratio (WHR) to predict this condition.

**Methods:** In our study, we analyzed data from 9,450 adults aged 18 to 80 years obtained from the NHANES 2017‐2018 dataset. We utilized the SIRI, calculated by multiplying the count of neutrophils by monocytes, then dividing the resulting value by the count of lymphocytes to predict heart failure via Receiver Operating Characteristic (ROC) analysis with various models. Initially, we evaluated the predictive ability of SIRI alone. Subsequently, we investigated the effectiveness of combining SIRI with BMI, WHR, and both BMI and WHR to determine the most suitable predictive index. Additionally, we employed three logistic regression models to examine the association between the most predictive inflammatory indices and heart failure.

**Results:** Four ROC analysis models were conducted, with the last ones being the most predictive, yielding an AUC of 0.70 (95% CI: 0.66‐0.74), an ideal cutoff point of 31.90, sensitivity of 0.64, and specificity of 0.63 [Fig. 1(a‐d)]. All logistic regression models revealed associations between the index derived from SIRI, BMI, and WHR and heart failure. In the first model, this new index was utilized independently, while the second model integrated hs‐CRP and ferritin levels. The third model included additional covariates such as age, gender, race, education, asthma, diabetes, hypertension, and eGFR. The results showed AOR of 3.07 (95% CI: 2.22‐4.26), 2.90 (95% CI: 2.08‐4.05), and 1.77 (95% CI: 1.24‐2.52) for each respective model.

**Conclusions:** The newly developed inflammatory‐obesity index demonstrates robust associations and predictive abilities for heart failure. Its affordability and accessibility render it suitable for disease screening.

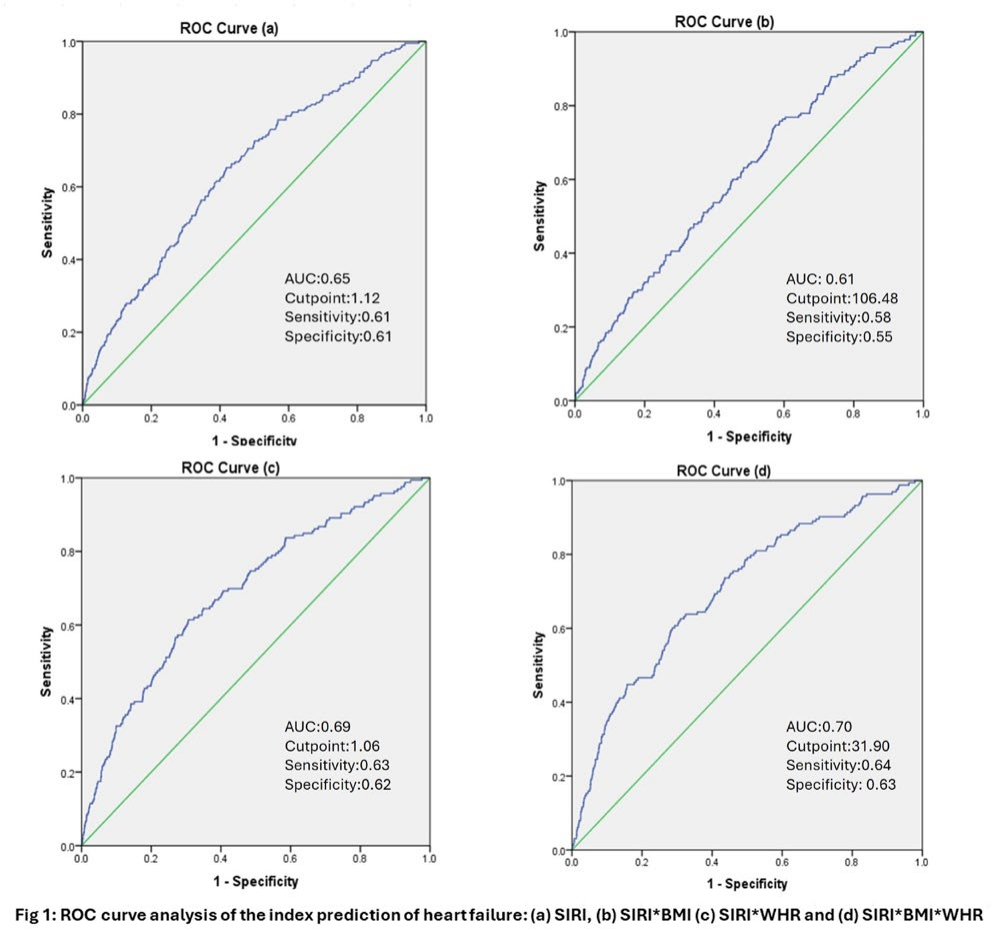

## 84 SEGMENTAL EVALUATION OF PREDICTIVE VALUE OF LEFT ATRIAL EPICARDIAL ADIPOSE TISSUE FOLLOWING CATHETER ABLATION FOR ATRIAL FIBRILLATION

### 84.1 **SHINICHI TACHIBANA** ^1^, OSAMU INABA^1^, SHIN MEGURO^1^, KENTARO NAKATA^1^, TOSHIKI MICHISHITA^1^, YUHEI ISONAGA^1^, HIROAKI OHYA^1^, TAKAMITSU TAKAGI^1^, YUKIHIRO INAMRA^1^, AKIRA SATO^1^, TETSUO SASANO^2^

#### 84.1.1 ^1^Japan Red Cross Saitama Hospital, Saitama, Japan,^2^Tokyo Medical and Dental University Hospital, Tokyo, Japan

**Introduction:** Left atrial epicardial adipose tissue (LA‐EAT) is associated with atrial tachyarrhythmias (AF/AT) recurrence after catheter ablation (CA) for atrial fibrillation (AF). However, no previous studies have evaluated the predictive value of AF/AT recurrence for each part of LA‐EAT.

**Methods:** The subjects of the current study comprised of consecutive patients undergoing initial CA for AF at Japan Red Cross Saitama Hospital (Saitama, Japan) from June to December 2022. In the patients with an eGFR≥30 mL/min/1.73m^2^ on blood tests, multidetector row computed tomography (MDCT) was performed preoperatively. The slice data of the MDCT image were reconstructed into a 3‐dimensional volume rendering using computer software (ZAIO station2, ZAIOSOFT, Tokyo, Japan), in which LA‐EAT was measured and defined as the tissue with CT values in the range of ‐190 to ‐30 Hounsfield units around the LA. LA‐EAT was divided into three regions: anterior‐EAT, posterior‐EAT, or interatrial septal adipose tissue (IAS‐AT).

**Results:** 350 patients (29.4% female, age 67.2 ± 10.8 years, paroxysmal AF (Paf) 53.7%) were analyzed. During a mean follow‐up of 351 ± 109 days, 56 patients (16.0%) experienced AF/AT recurrence. The mean LA‐EAT volume was 20.7±11.1ml and LA‐EAT≥26.8ml was an independent risk factor for AF/AT recurrence (HR 2.21 (95% confidence interval (CI):1.24‐3.93), P=0.007). Receiver operating characteristic analyses revealed the area under the curve of IA‐EAT was 0.652 (95%CI: 0.573‐0.732) with an optimal cut‐off point of 1.3ml (sensitivity:0.740, specificity:0.497), which was significantly better than anterior‐ or posterior‐EAT in predicting recurrent AF/AF. Multivariate analysis showed IAS‐AT was an independent predictor for AF/AT recurrence in patients with persistent AF (Pef) (HR 3.52 (95%CI, 1.52‐8.13); P=0.003), but not in patients with Paf.

**Conclusions:** LA‐EAT is a predictor for AF/AT recurrence after CA for AF, in which IAS‐AT was significantly better in predicting recurrent AF/AT than other parts of LA‐EAT. IAS‐AT was an independent predictor of AF/AT recurrence in patients with Pef.

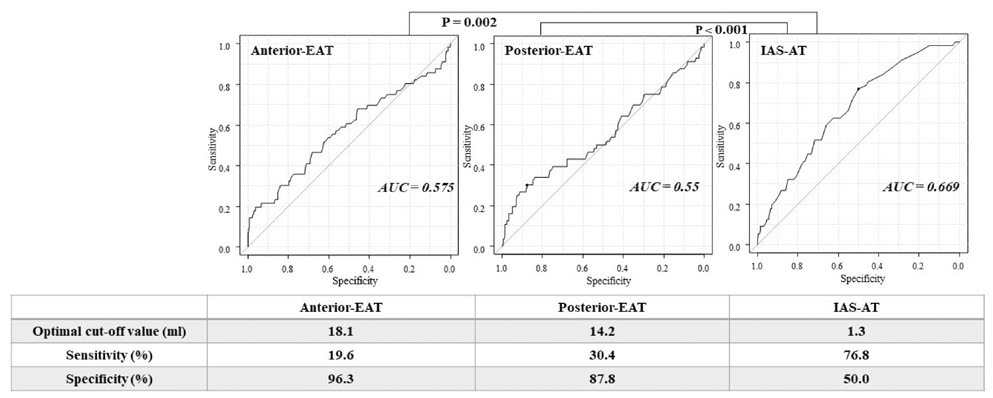

## 85 RADIOFREQUENCY CATHETER ABLATION FOR LONG STANDING PERSISTENT ATRIAL FIBRILLATION IN OVER 360 PATIENTS

### 85.1 **TAKAMITSU TAKAGI**, KENTARO NAKATA, SHINICHI TACHIBANA, HIROAKI OHYA, YUKIHIRO INAMURA, AKIRA SATO, OSAMU INABA

#### 85.1.1 Japanese Red Cross Saitama Hospital, Saitama, Japan

**Introduction:** Catheter ablation for long standing persistent atrial fibrillation (LSAF, lasting duration >12 months) is challenging and its indication should be appropriately determined. The purpose of this study was to identify predictors of sinus rhythm maintenance after ablation and to examine the cut‐off for AF lasting duration for which ablation improves prognosis.

**Methods:** We enrolled 361 patients (63.4±10 years old, female 75) who underwent radiofrequency catheter ablation for LSAF between 2016 and 2022 at our institution. Circumferential pulmonary veins isolation (PVI), posterior wall isolation (PWI) and cavotricuspid isthmus ablation were basically performed. If needed, non‐PV trigger ablation, other linear ablation and substrate modification were added. Clinical parameters and outcomes were evaluated.

**Results:** PVI was successfully performed in all patients and 91% of patients received PWI. After lesion set ablation, 87% of patients underwent induction of immediate recurrence AF with high‐dose ISP infusion, of which 28% had the non‐PV trigger and were ablated. During a median follow‐up period of 1.1 years, 42% of patients had recurrent arrhythmia and 28% received repeat procedures (mean 1.3 procedures). After the last procedure, recurrent arrhythmia was observed in 25% of patients. Multivariate analyses revealed that AF lasting duration was the only significant predictor for first AF recurrence (OR 1.01, 95% CI 1.01‐1.02; p<0.001). When comparing the persistent AF group (<12 months) and LSAF groups divided by AF duration with propensity score matching analysis, patients with AF duration of 36 months or more had significantly worse outcomes.

**Conclusions:** In this study of over 360 cases of LSAF ablation, long duration of AF was independent predictors of worse clinical outcomes. Patients with AF of more than 36 months duration have a significantly lower rate of maintenance of sinus rhythm after ablation than those with less than 36 months duration.

## 86 OBSERVATIONS ON THE USE OF 2 DYNAMIC ATRIOVENTRICULAR DELAY ALGORITHMS TO OPTIMIZE LEFT BUNDLE BRANCH PACING CARDIAC RESYNCHRONIZATION THERAPY

### 86.1 **JANNAH LEE TARRANZA**, ERIC TIEN SIANG LIM, JULIAN CHEONG KIAT TAY, GERMAINE JIE MIN LOO, JONATHAN ONG WEI SHENG, HOOI KHEE TEO, XUANMING PUNG, KAH LENG HO, DANIEL THUAN TEE CHONG, CHI KEONG CHING

#### 86.1.1 National Heart Center Singapore, Singapore, Singapore

**Introduction:** Dynamic adjustment of the atrioventricular (AV) delay is desirable due to diurnal variation of AV conduction; however, manufacturer‐specific dynamic AV delay algorithms (Autoadapt, SyncAV and AdaptivCRT) have been studied in cardiac resynchronization therapy (CRT) cases which use coronary sinus (CS) leads and not left bundle branch (LBB) pacing leads.We present two cases of LBB‐pacing CRT and highlight our observations on the use of dynamic AV delay algorithms to optimize QRS morphology and duration in such cases.

**Methods:** N/A

**Results:** Case 1: A 62‐year‐old man with non‐ischemic dilated cardiomyopathy and LBBB with QRS duration (QRSd) of 201ms was referred for CRT. He received a LBB pacing‐optimized CRT (LOT‐CRT) implantation. Post‐implant optimization of the QRS showed elimination of the terminal r in V1 and reduction in the QRSd from 201ms to 112ms with biventricular pacing. Adaptive AV reduction parameter needed to be increased to 0.9 to maintain the optimized QRS.Case 2: A 70‐year‐old female with ischemic cardiomyopathy and LBBB with a QRSd of 170ms was referred for CRT. Post‐implant optimization of the QRS showed that with biventricular pacing via the LBB and CS leads together with manual adjustment of the AV delay, it was possible to eliminate the terminal r in V1 and reduce QRSd to 100ms. We observed that the nominal SyncAV of ‐50ms needed to be increased to ‐90ms to maintain this optimized QRS.

**Conclusions:** We show that adjusting the AV delay to allow RBB conduction to fuse with LBB‐pacing eliminates the terminal r wave associated with LBBP, reduces QRSd and leads to QRS morphologies closely resembling native conduction. Dynamic AV delay algorithms such as Autoadapt and SyncAV may be used to help overcome the normal diurnal variation of AV conduction; however, its nominal settings which have been optimized for CS leads need to be adjusted for LBB‐pacing leads. We note that the proprietary Medtronic AdaptivCRT which does not allow adjustment of the dynamic AV delay may be less suitable for this purpose, and implanters should bear this in mind when choosing the pulse generator for such cases.

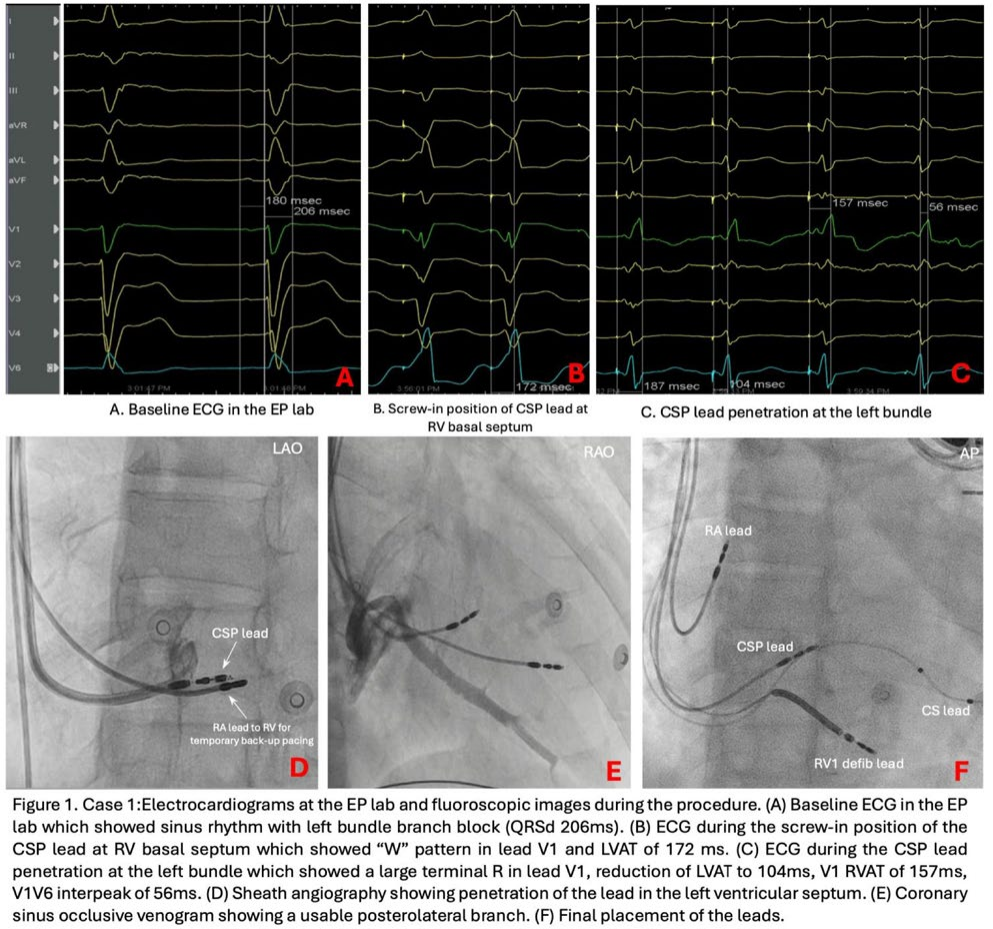

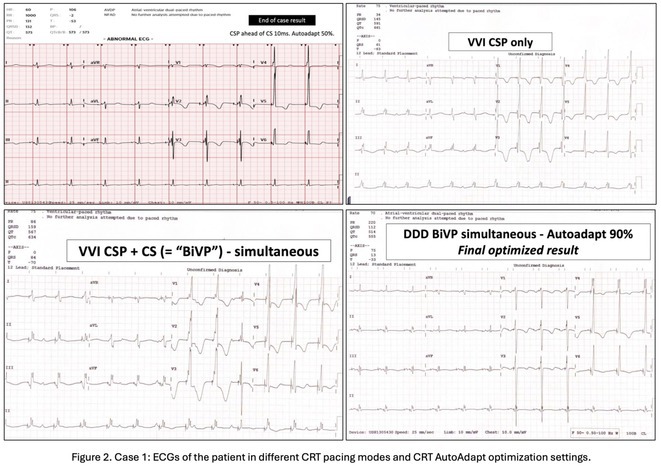

## 87 PREDICTIVE VALUE OF INDUCIBILITY TEST FOLLOWING CATHETER ABLATION OF ATRIAL FIBRILLATION: A SYSTEMATIC REVIEW AND META‐ANALYSIS

### 87.1 **SABRINA TELAGA** ^1^, NABIEL HAYKAL^1^, OMAR SYARIF^1^, M. FAHD ABDURRAHMAN^1^, SHALIHANA RAMADITA^1^, SHAKIRA AMIRAH^1^, JAUDA HANOON^1^, SUNU RAHARJO^2^

#### 87.1.1 ^1^University of Indonesia, Jakarta, Indonesia,^2^Department of Cardiology & Vascular Medicine, University of Indonesia, Jakarta, Indonesia

**Introduction:** Although catheter ablation (CA) is an effective treatment for atrial fibrillation (AF) patients, the predictive value of non‐inducibility post‐ablation remains controversial. We aim to evaluate the predictive value of non‐inducibility post‐CA with long‐term atrial arrhythmia recurrence.

**Methods:** We conducted a systematic review and meta‐analysis based on PRISMA using PubMed, Cochrane, Scopus, and ProQuest. Cohort studies of AF ablation were included. The relative risk of atrial arrhythmia recurrence at follow‐up was assessed as the primary outcome using a fix‐effects model.

**Results:** Seventeen cohort studies with non‐inducible AF (n=2067) and inducible AF (n=1391) post‐CA were analyzed. Overall, at a median follow‐up of 12 months, non‐inducible AF post‐CA was associated with a lower risk of atrial arrhythmia recurrence compared to inducible AF (RR 0.68 [95% CI 0.58‐0.79]) (Figure 1). This finding was consistent for both paroxysmal AF (RR 0.67 [95% CI 0.59‐0.76]) and non‐paroxysmal AF (RR 0.76 [95% CI 0.61‐0.94]). Interestingly, the inducibility test using isoproterenol has a better predictive value (RR 0.29 [95% CI 0.16‐0.52]) than atrial burst pacing (RR 0.68 [95% CI 0.59‐0.79]), while an inducibility test with adenosine has no predictive value (RR 1.0 [95% CI 0.67‐1.50]).

**Conclusions:** Non‐inducibility of AF post‐catheter ablation is associated with lower atrial arrhythmia recurrence, and the isoproterenol induction test has the best predictive value of atrial arrhythmia recurrences.

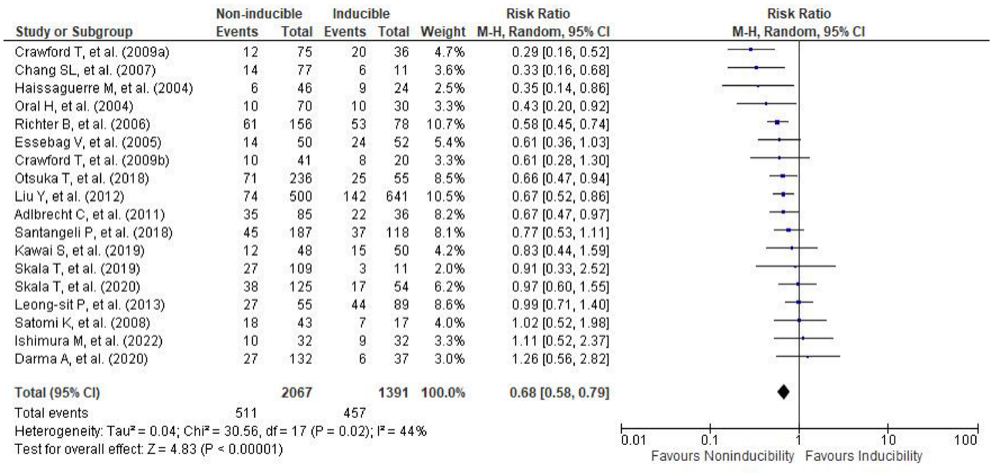

## 88 FEASIBILITY OF NOVEL DOUBLE BALLOON ULTRA SHORT DURATION STEAM POWER ABLATION TECHNOLOGY FOR ATRIAL FIBRILLATION

### 88.1 **VENKAT N. THOLAKANAHALLI** ^1^, KEVIN HOLBROOK^2^, BRAD TRAEGER^2^, BO CLAYMORE^3^, HOLGER FRIEDERICH^2^

#### 88.1.1 ^1^VA Medical Center University of Minnesota, Minneapolis, MN,^2^Aquaheart Inc, Santa Ana, CA,^3^Resolution Medical, Fridley, MN

**Introduction:** AF is a major global cause of morbidity and heart failure (HF) with a prevalence of 50 million as of 2020. Rhythm management is class I recommendation in HF and early AF intervention improves clinical outcomes. Many techniques for PVI being cornerstone therapy available, have merits and limitations, hence the quest for newer technologies continues. We’d like to demonstrate this novel steam power ablation (SPA) technique for PVI which are well known in other areas of medicine.

**Methods:** Using Aquaheart double balloon ablation catheter (ABA) (Figure.1A), dosimetry testing using chicken breast tissue as well as porcine liver in saline bath at 37 deg C was performed. Thermocouples were used to measure tissue temperature. Single treatments were applied at 10, 20, 30, and 40 seconds. Double treatment dose durations in same order with a delay of 10‐ and 60‐seconds were tested (Figure.1C). Tissue temperatures and lesion dimensions were measured. ABA coagulation in heparinized blood bath at 37 deg C was assessed. SPA using ABA was similarly used in 6 porcine and 4 canine models with steam infused through inner balloon to a temperature of ~ 80 deg C with outer balloon inflated in right and left atria and the lesions were assessed using voltage maps and necropsy.

**Results:** The lesion size observed on bench testing was predictable and incremental by duration and stacking with tissue temperature consistently between 50 and 60 deg C (Figure.2). SPA demonstrated predictable lesions without collateral damage (Figure.1B). There were no tissue charring or steam pops noted. Thrombus layer was only seen in bench testing over 50 seconds duration. Similar results were observed in acute animal studies as well.

**Conclusions:** This SPA is a single shot novel technology which is ultra‐short duration with unique predictable lesion characteristics effective for PVI. Chronic animal studies currently are ongoing. Further human studies will help confirm our conclusions derived from the acute studies.

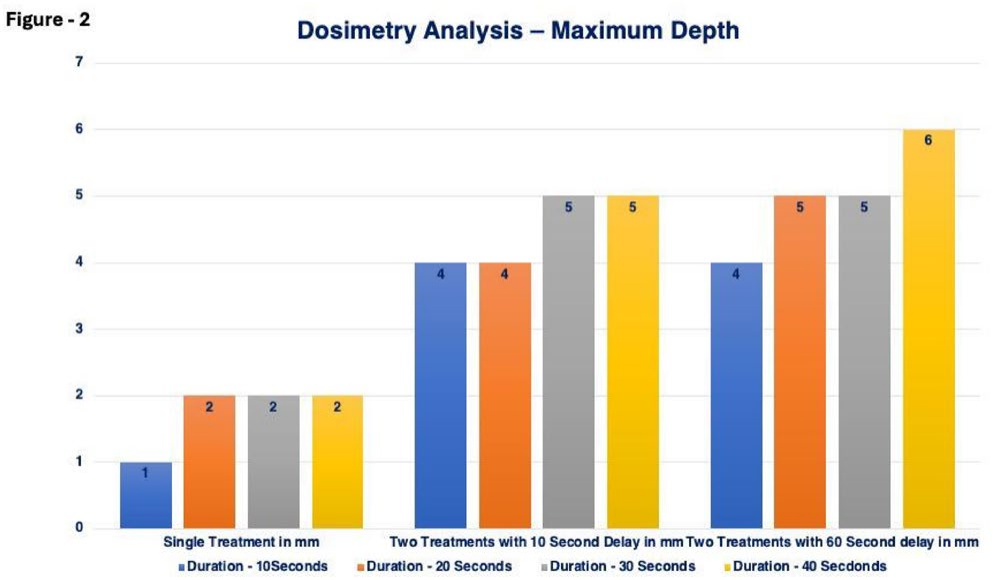

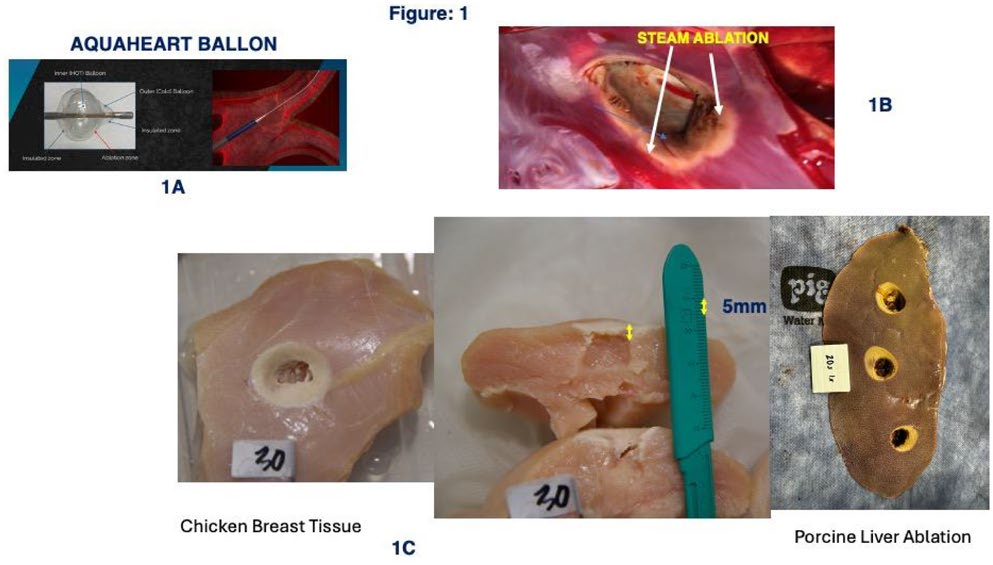

## 89 IS PREMATURE VENTRICULAR COMPLEX ABLATION A CURE FOR SYSTOLIC HYPERTENSION? A PILOT STUDY

### 89.1 **VENKAT THOLAKANAHALLI** ^1^, BALAJI KRISHNAN^2^, ASHWINI SANKAR^3^, STEPHANIE LI^4^, AKSHAY KRISHNA^3^, SMITHA MURTHY^1^, STEFAN BERTOG^1^

#### 89.1.1 ^1^VA Medical Center, University of Minnesota, Minneapolis, MN,^2^Park Nicollet Medical Center, Minneapolis, MN,^3^University of Minnesota, Minneapolis, MN,^4^SUNY Downstate Medical Center, Brooklyn, NY

**Introduction:** The impact of catheter ablation for frequent PVCs and post ablation systolic blood pressure (SBP) has not been previously assessed. Based on our previous study PVC ablation has been associated with improvement in diastolic function. We would like to share the results of this pilot study demonstrating one‐year consistent reduction of SBP after PVC ablation.

**Methods:** This retrospective study included patients with a PVC burden ≥10%/24h who underwent radiofrequency ablation (RFA) and were subsequently followed for > 1 year. Patients with successful ablation (group A) while those with unsuccessful ablation (group B) were compared. Successful PVC ablation was defined as > 80% PVC burden reduction on 24‐hour Holter monitor. Office‐based BP measurements were used for analysis.

**Results:** Eighty‐five patients (age 69 ± 11 [SD] years, 100% male) were included in the study. Of these, 58 (68%) were in group A, and 27 (32%) patients in group B. No statistical difference noted between groups on day one, 20.1± 11.6 vs. 12.2 ± 10.8 (p=0.094). A significant difference was noted at 3 months 12.4± 10.5 vs. 5.2±12.3 (p=0.0096) (Figure 1). Although this effect did not seem to persist at 1 year 15.6± 10.4) vs 11.5± 13.8 (p=0.1237) but there was a significant difference in reduction in the total number of blood pressure medications (p=0.012), ACEI (p=0.0285), and calcium channel blockers (p=0.0285) used at 12 months in group A. Overall the SBP significantly dropped compared between pre‐ablation and one year follow‐up in group A. Binominal logistic regression analysis identified a diagnosis of resistant hypertension, successful RFA, PVC burden as predictors of a >20 mm Hg reduction in systolic blood pressure following RFA.

**Conclusions:** Radiofrequency PVC ablation is associated with a substantial and sustained blood‐pressure reduction in patients with resistant hypertension. The next step is to design a randomized trial comparing medical therapy versus ablation therapy for PVCs who have hypertension on two or more anti‐hypertensive drug therapy.

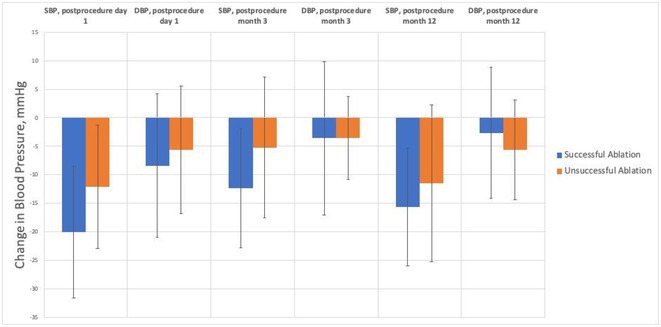

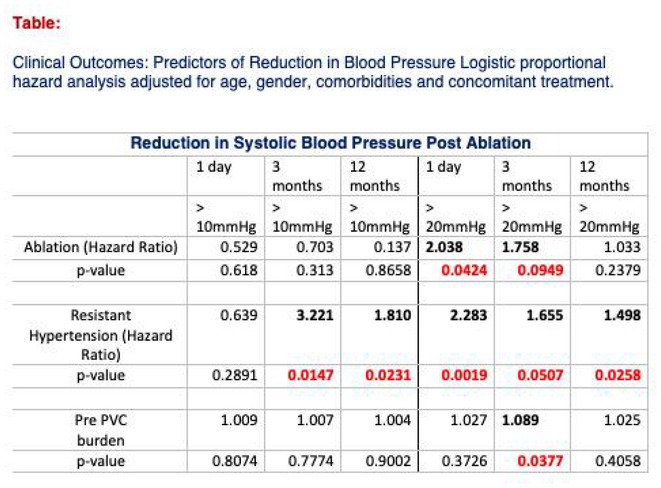

## 90 LV‐ONLY CARDIAC RESYNCHRONIZATION THERAPY WITH ELECTROCARDIOGRAM PHYSIOLOGIC OPTIMIZATION: A PARADIGM SHIFT

### 90.1 TRAN THONG

#### 90.1.1 Systolic Medical Systems, Beaverton, OR

**Introduction:** In patients with left bundle branch block and good A‐RV conduction, LV‐only (LVo) CRT was recognized in the 2013 ESC guidelines as non‐inferior to bi‐ventricular (biV) CRT with increased service life, reduced atrial fibrillation risk. LVo programs need individualized AV delay optimization.

An LVo algorithm with ECG‐based optimization was developed where the Autonomic Nervous System (ANS), which regulates cardiac output (CO) in a closed loop feedback system, is recruited to synchronize the ventricles when the patient exercises.

**Methods:** The optimal LV AV delay, derived from sensing and LV threshold tests at follow‐ups, allows the peak intrinsic A‐RV depolarization to coincide with the peak LVp depolarization. The AV delay is then shortened for the LVp peak to occur just before the A‐RV peak.When patients exercise, the ANS shortens A‐RV to synchronize depolarizations yielding a small CO boost, enabling increased ANS modulations. This is a paradigm shift in CRT!Since the RV lead is not used, a dual chamber (RA &LV) CRT (dcCRT) was proposed.In an LV lead implantation training in July 2023, patients were implanted dcCRTs. In November, 100‐day follow‐ups were performed on 15 dcCRT patients.

**Results:** ‐ LVEF Fig. at top: ΔLEVF: +2 to +29%, +14.0± 8.9%, med +15.0%. 9 patients with ΔLEVF >+10%.

Thoracic impedance, Z, is measured 182 times/day. 2 patients had Z turned OFF. Increased Z in all 13 patients reflect reduced lung fluid due to improved LV blood flow: effective therapy!

‐ Z Fig. at bottom: ΔZ: +25.0±5.1 Ω, med +23.7 Ω. 3 patients with ΔLEVF<+10%, have ΔZ>+20 Ω!

At the 100‐day follow‐ups, using +10% ΔLVEF or +20Ω ΔZ as criteria for super‐response (SR), SR of 12/15 (80%) to 12/13 (92.3%)!

**Conclusions:** The follow‐ups of the ECG optimized LVo CRT of 15 patients with dcCRT show results superior to reported biV studies, thanks to the optimization. This is just at 100 days! This indicates a potentially superior alternative to biV CRT.While the study was done with dcCRT devices, results are applicable to 3 chamber CRT‐P/‐Ds with LVo pacing, including already implanted devices!

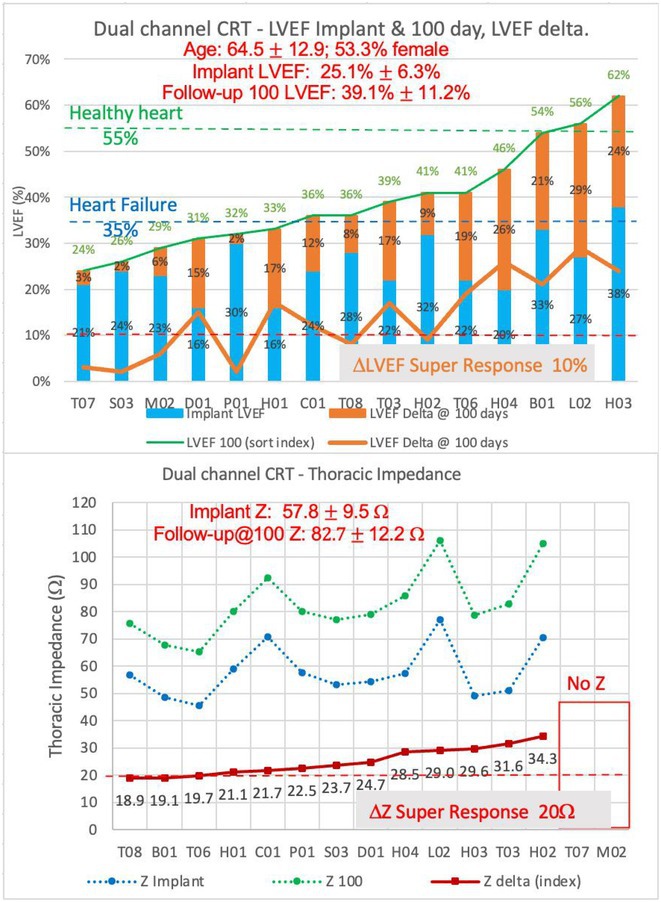

## 91 THE EFFECT OF CATHETER ABLATION OF TYPICAL ATRIAL FLUTTER ONATRIAL REMODELLING ANDVENTRICULARFUNCTION

### 91.1 **ADRIANA TOKICH** ^1,2^, LUKAH Q. TUAN^1,2,1^, NATASHA JONES‐LEWIS^1,3^, LORI BELL^1,3^, JENISH SHROFF^1,3^, ANUGRAH NAIR^1,3^, RAJEEV K. PATHAK^1,3^

#### 91.1.1 ^1^Canberra Heart Rhythm, Canberra, Australia,^2^Australian National Univeristy, Canberra, Australia,^3^Australian National University, Canberra, Australia

**Introduction:** Cavo‐tricuspid isthmus (CTI) dependent atrial flutter (AFL) is one of the most common atrial arrhythmias involving the right atrium (RA). Radiofrequency catheter ablation has been widely used as a therapy of choice and it is curative. Patients with AFL often have dilated RA and have reduced function. However, there is limited data on the effect of this intervention on cardiac size and function. CTI dependent ablation for patients with AFL will improve tricuspid valve function, bi‐atrial enlargement, and ventricular function.

**Methods:** A prospective study was conducted on 156 patients who underwent CTI dependent ablation for clinical typical AFL at a single institution between 2017 and 2023.Echocardiographic data were analysed at baseline prior to ablation, and at early follow‐up within 1‐year post‐ablation. Follow‐up echocardiographic data was available for 153 patients.

**Results:** Of the 156 patients with CTI‐AFL, 139 had typical counter‐clockwise flutter. Other 17 had scar dependent or atypical RA flutter. The mean age was 72.2±7.1 years old with 22% female. The average left ventricular (LV) ejection fraction (EF) significantly improved on follow‐up echo (41±11 to 54±2%, p=0.003). 123 (79%) patients had an increased EF of 20% or more. The prevalence of moderate to severe tricuspid regurgitation (TR) was 18% (n=28) at baseline and 5% (n=8) at follow‐up with no significant difference (p=0.051). Echocardiography also showed improvement of RA size in 51% and left atrial (LA) size in 43.2% of the patients.

**Conclusions:** Patients who underwent CTI dependent AFL ablation showed an improvement in cardiac size and function at follow‐up evaluation. This immediate improvement was sustained at 12 months. These findings suggest that restoration of sinus rhythm from atrial flutter is associated with improvement in TR severity, RA size, LA size, and LVEF.

## 92 TIPS AND TRICKS FOR BROKEN CATHETER REMOVAL

### 92.1 LE UYEN PHUONG TRAN

#### 92.1.1 Cho Ray Hospital, Ho Chi Minh city, Viet Nam

**Introduction:** Sudden broken catheter while doing electrophysiology studies is rare, but it may happen and need appropriate skills to be safely removed without damaging blood vessels and avoid vascular surgery.

**Methods:** We describe 2 cases in which the ablation catheters were broken while doing electrophysiology studies. We used snares with some tips to withdraw the catheters by introducing another large bore sheath in the other femoral vein to easily catch to tip of the broken catheters.

**Results:** If we tried to pull out the broken catheter immediately, the cover of the catheter would be peeled and stuck at the tip of the sheath and may tear the vessel walls, which need to be repaired by surgery. To safely remove the broken catheter, we inserted another large bore sheath available in EP labs in the contralateral femoral vein and modified the tip of the sheath to make it become bigger. Then we used a snare to catch the very distal tip of the catheter and withdraw it to that large bore sheath. Then we pulled the whole system out of the femoral vein and cut the broken catheter more proximally from the broken line. The proximal catheter was then taken out easily.

**Conclusions:** There are necessary tips to remember in electrophysiology labs to help us overcome unpredicted complications.

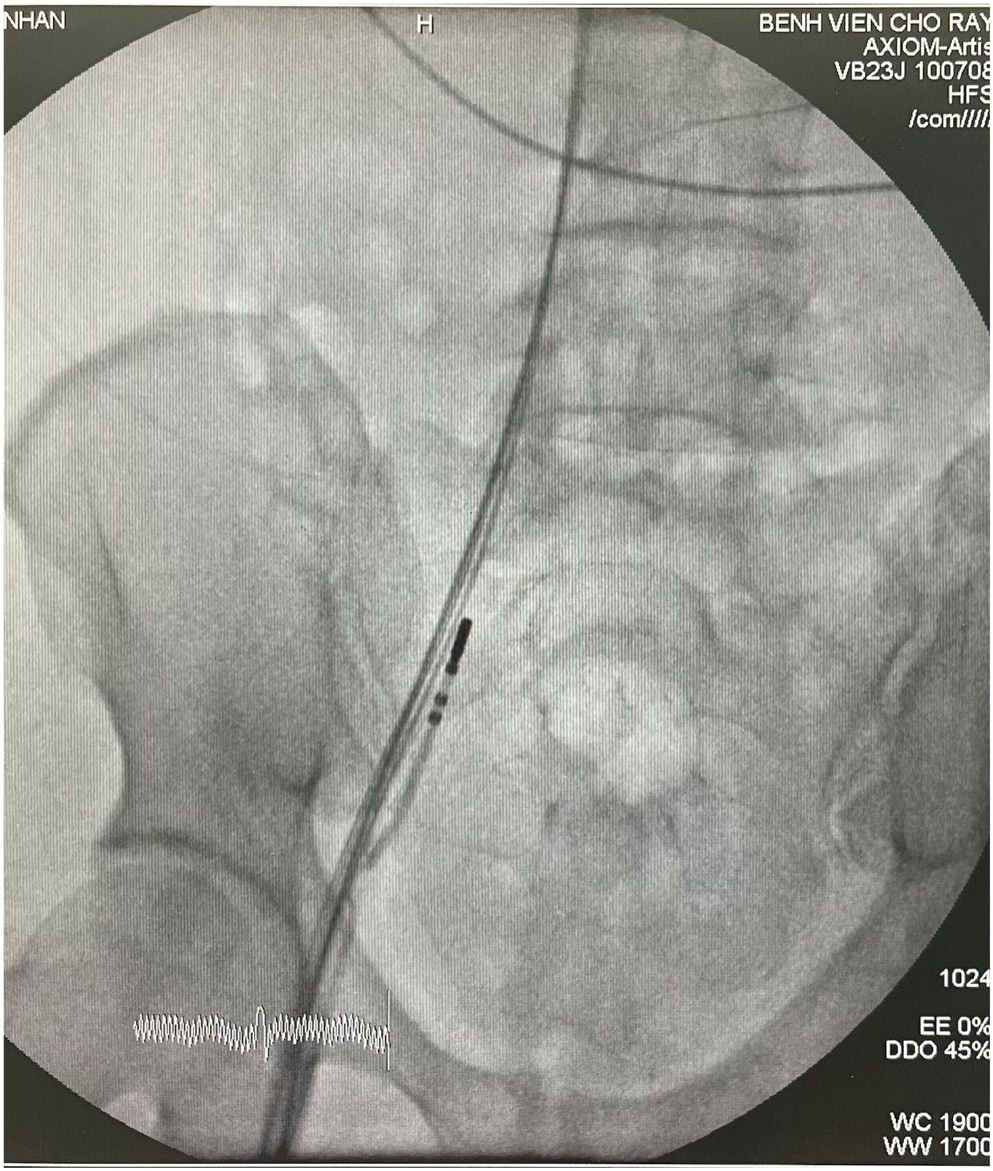

Chair

**S. Tsao**;

Children's Hospital Hong Kong, Hong Kong, Hong Kong

## 93 MITRAL ISTHMUS ABLATION WITH COMBINED RADIOFREQUENCY AND ETHANOL ABLATIONS TO VISUALIZED MARSHALL VEIN ON CARTO‐UNIVU

### 93.1 **AYAKA USUI** ^1^, SHU YAMASHITA^1^, YASUHIRO SHIRAI^1^, TAKESHI SASAKI^1^, KYOJI HAYASHI^1^, RYOTARO FUNAYAMA^1^, SAYUMI NOZAKI^1^, SHOTA SEGAMI^1^, JUNICHI DOI^1^, KAZUTO HAYASAKA^1^, SHIGEO SHIMIZU^1^, SHINSUKE MIYAZAKI^2^, TETSUO SASANO^2^

#### 93.1.1 ^1^National Hospital Organization Disaster Medical Center, Tokyo, Japan,^2^Tokyo Medical and Dental University, Tokyo, Japan

**Introduction:** The musculature around the vein of Marshall (VOM) may cause failed mitral isthmus (MI) ablation, which requires ethanol ablation to the VOM (EA‐VOM). Visualizing the VOM on 3D anatomical map may be helpful for both radiofrequency ablation (RFA) and EA‐VOM. This study investigates the impact of visualizing the VOM on 3D map with CARTO‐UNIVU on creating a MI block by combining RFA and EA‐VOM in patients with persistent atrial fibrillation (PEF).

**Methods:** This study consists of 383 patients who underwent de novo AF ablation to treat PEF. In all patients, MI ablation was performed by a combination of endocardial left atrial (LA) RFA, epicardial RFA within the coronary sinus (CS) and EA‐VOM in addition to pulmonary vein isolation and LA roof ablation using cryoballoon, and superior vena cava isolation and cavo‐tricuspid itshmus ablation by RFA. CS venogram was performed with a 7F guiding catheter via the internal jugular vein or a 5Fr JR catheter via the femoral vein to identify the presence of VOM, followed by visualization of VOM on 3D map with CARTO‐UNIVU. EA‐VOM was performed in patients with VOM despite the completion of MI block and endocardial ablation targeting at VOM on CARTO UNIVU was also performed.

**Results:** MI block was successfully created in 374 of 383 patients (97.9%). CS venogram identified the presence of VOM in 325 patients (84.9%), of whom EA‐VOM was attempted in 288 (88.6%). Consequently, EA‐VOM was performed without severe complications in all patients (100%). Regarding the outcomes of EA‐VOM, the average number of ethanol infusions was 2.6±0.6 and the average volume of ethanol was 5.0±2.1ml. At 1 year of follow‐up, 83.1% of the patients in this study were free from recurrences of atrial arrhythmias. Mild pericarditis was observed in 7 patients (1.8%), however no cardiac tamponade requiring drainage was observed.

**Conclusions:** MI block was successfully created in most patients with anatomical guidance by CARTO‐UNIVU in both endocardial RFA targeting at VOM and EA‐VOM. Visualization of the VOM on 3D maps may improve the outcomes of MI ablation.

## 94 STELLATE GANGLION BLOCK TECHNIQUE FOR REFRACTORY VENTRICULAR TACHYCARDIA:A SYSTEMATIC REVIEW AND META‐ANALYSIS

### 94.1 **RUAN VLOK** ^1^, SRISA BODDUPALL^2^, ROHAN KAUL^3^, LACHLAN DONALDSON^1^, ADAM LEE^3^, JAMIE CHAM^3^

#### 94.1.1 ^1^The George Institute, Sydney, Australia,^2^Liverpool Hospital, Sydney, Australia,^3^Campbelltown Hospital, Sydney, Australia

**Introduction:** Refractory ventricular arrhythmias (VA) are a medical emergency. Stellate Ganglion Block (SGB) has been proposed as a means of neuromodulation to treat refractory VA. The evidence for the role of SGB is limited. It is unknown whether unilateral or bilateral, or single dose or continuous infusion SGB are associated with better outcomes or increased complications.

**Methods:** We performed a study level meta‐analysis of studies that report rates of VA suppression, short‐term mortality, need for repeat SGB and SGB related complications in refractory VT. Our primary objective was to describe the incidence clinically significant VA suppression following SGB. Subgroup analyses were performed for unilateral vs bilateral SGB and continuous infusion vs bolus dose SGB. Four databases were systematically reviewed by two authors.

**Results:** 26 studies met our inclusion criteria. All studies were observational, and no low risk of bias studies were included. Successful arrhythmia suppression in the first 24hrs following SGB occurred in 348 of 468 patients (ES 0.80 [0.72‐0.88] *I* ^*2*^= 77.96%). More patients managed with continuous infusion SGB (0.94 [0.86‐1.02] *I* ^*2*^ *=0.0*) achieved successful arrhythmia suppression than patients managed with bolus dose SGB (0.78 [0.69‐0.88] *I* ^*2*^ *=*77.90), p=0.01. Unilateral vs bilateral SGB was not associated with a difference in arrhythmia suppression. Unilateral vs bilateral and continuous infusion vs bolus SGB were not associated with differences in the incidence of mortality or the need for repeat SGB.

**Conclusions:** SGB was associated with a high proportion of patients achieving clinically significant arrhythmia suppression in the first 24hrs following SGB. Continuous infusion SGBs are associated with a higher proportion of patients achieving successful arrhythmia suppression.

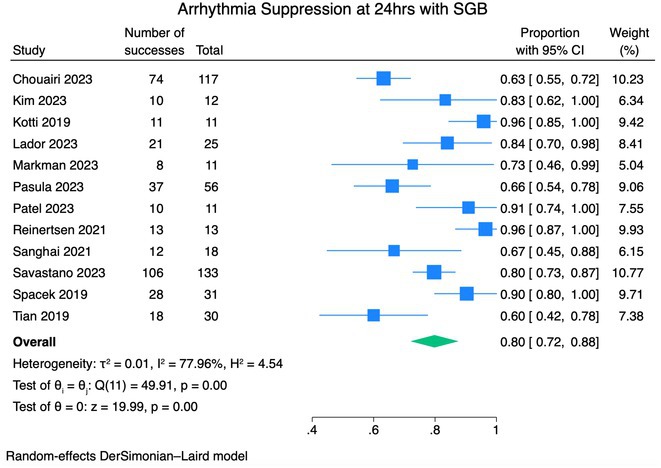

## 95 ZERO‐FLUOROSCOPY CATHETER ABLATION OF OUTFLOW TRACT PREMATURE VENTRICULAR CONTRACTIONS IN 12 WEEKS OF PREGNANCY: A CASE REPORT

### 95.1 **YOGA WARANUGRAHA** ^1,2,3^, MUHAMMAD MAHDI ALATTAS^2^

#### 95.1.1 ^1^Universitas Brawijaya, Malang, Indonesia,^2^Persada Hospital, Malang, Indonesia,^3^Universitas Brawijaya Hospital, Malang, Indonesia

**Introduction:** Frequent premature ventricular contractions (PVCs) in pregnancy may cause poor outcomes. For drug‐refractory or poorly tolerated PVCs, radiofrequency catheter ablation (RFCA) using non‐fluoroscopic approach should be considered to avoid further maternal and fetal adverse effects, preferably after the first trimester.

**Methods:** N/A

**Results:** A 33‐year‐old female in 12 weeks of pregnancy came to the emergency room due to severe palpitations and chest discomfort. The patient also experienced four syncope episodes in the last month. The electrocardiogram (ECG) revealed PVCs with bigeminy episodes originating from the free wall of the right ventricular outflow tract (RVOT). Holter showed a PVC burden of 34%. After all reversible causes of PVC were excluded, zero‐fluoroscopy catheter ablation was conducted to prevent further maternal and fetal complications. An 8F sheath was inserted in the right femoral vein. After that, bi‐directional irrigated‐tip ablation catheter was advanced from the femoral vein to the RVOT for PVC mapping and RFCA. The electroanatomic cardiac mapping system guided catheter placement and PVC mapping. The inferior vena cava (IVC)‐right atrium (RA) junction, superior vena cava (SVC)‐RA junction, and tricuspid rim were identified and used as the anatomical reference to advance the ablation catheter to the RVOT. The earliest local activation time (LAT) of ‐59 milliseconds was found in the anterior free wall part of RVOT. RFCA was performed several times to eliminate PVCs (Power: 20 to 30 watts, duration: 30 seconds, flow rate: 10 to 20 mL/minute). Multiple injections of 10 mcg of Epinephrine during the awake state were given to induce PVC. No PVCs were noted during 15 minutes of observation. No complications occurred.

**Conclusions:** Although guidelines recommend that catheter ablation be performed after the first trimester of pregnancy, this case report shows that zero‐fluoroscopy catheter ablation for RVOT PVCs is safe and beneficial when performed in the first trimester.

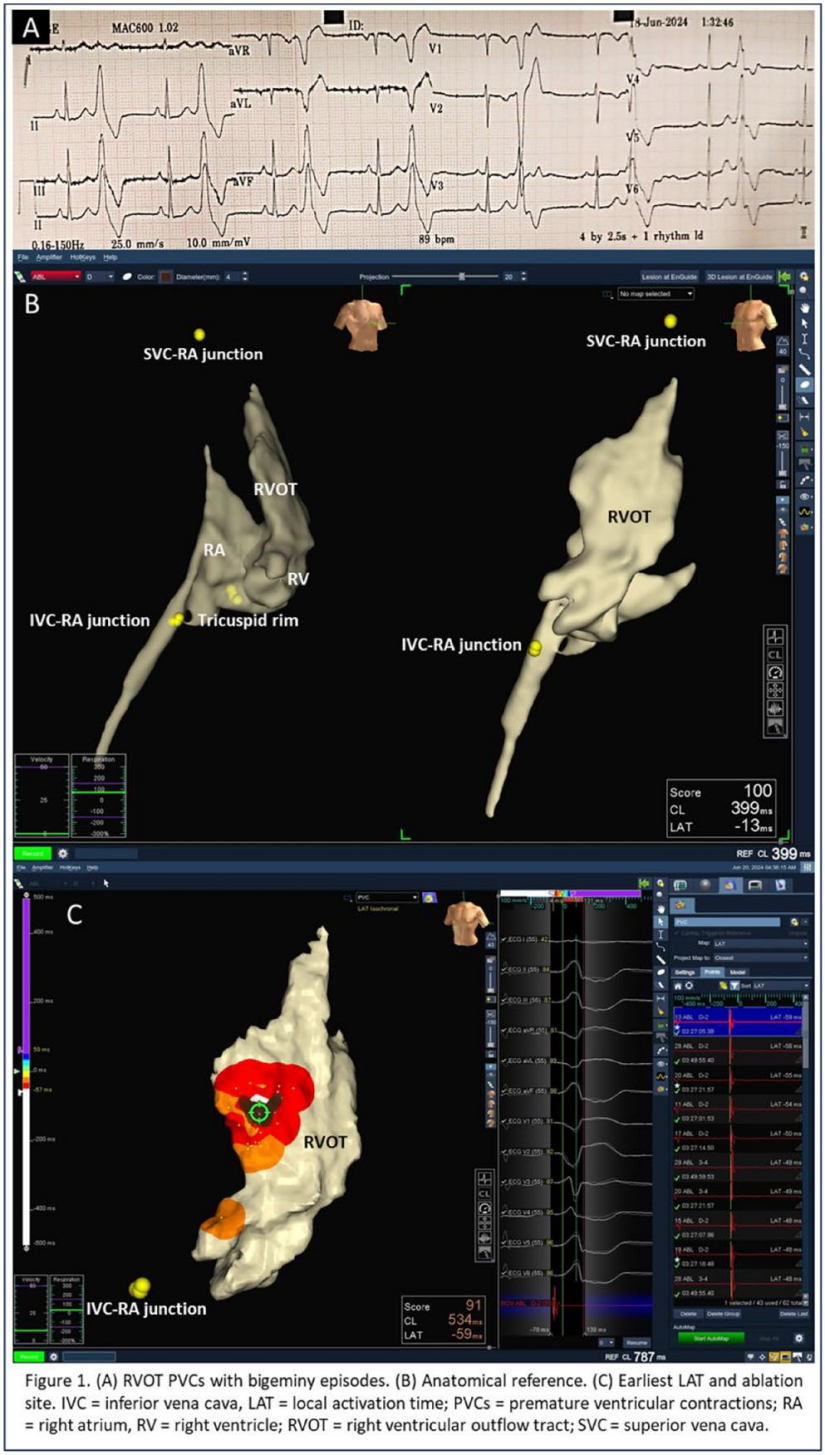

## 96 ENHANCEMENT OF LEFT ATRIAL FUNCTION IN PACEMAKER PATIENTS: INVESTIGATING THE ROLE OF LEFT ATRIAL STRAIN RESERVOIR DYNAMICS IN SINUS NODE DYSFUNCTION AND ATRIOVENTRICULAR NODE DYSFUNCTION

### 96.1 **AGUNG PRASETYO WICAKSONO**, FERA HIDAYATI, ERIKA MAHARANI, LUCIA KRIS DINARTI

#### 96.1.1 Department of Cardiology & Vascular Medicine, Faculty of Medicine, Public Health and Nursing, Universitas Gadjah Mada – Dr. Sardjito Hospital, Sleman, Yogyakarta, Indonesia

**Introduction:** Permanent pacemaker (PPM) improves clinical outcomes and quality of life in those with bradyarrhythmias. Dysnchrony of contractions in PPM patients may affect the structure and mechanoelectricity of the myocardium, leading to pacemaker‐induced cardiomyopathy. Left atrial strain reservoir (LASr) may detect early myocardial remodeling. The effect of PPM implantation on LASr alterations is currently unknown and has not been extensively investigated.

**Methods:** This is an observational study with a single pretest‐postest design by using ALEKA registry from sinus node dysfunction (SND) / atrioventricular node dysfunction (AVND) patients who underwent PPM implantation at Dr. Sardjito Hospital between November 2022 ‐ August 2023. LASr was assessed from 2D speckle tracking echocardiography prior to PPM implantation and 3‐12 months subsequently.

**Results:** Sixty one individuals with a median age of 70 years had fulfilled the inclusion and exclusion criteria, temporary pacemaker (TPM) insertion was needed in 44% (n=27), 56% (n=34) were female, 54% (n=33) with high ventricular pacing burden, 64% (n=39) with double chamber PPM, and 36% (n=22) with DDDR mode. For a median evaluation period of 8 months, there was a significant increase in LASr (19.15±7.98% and 22.57±8.78%, p=0.008, 0.94‐5.90 CI 95%). Multivariate analysis showed the change in LASr was affected by a condition of TPM insertion was needed prior to PPM implantation with regression coefficient 7.755 (p=0.001, 3.14 ‐ 12.37 CI 95%). Subject with a condition of TPM insertion was needed prior to PPM implantation showed lower baseline of LASr (15.93%±5.06% and 21.71±8.96%, p=0.004, 1.91‐9.65 CI 95%).

**Conclusions:** PPM implantation enhance LASr in symptomatic SND/AVND patients. Patients with a condition of TPM insertion was needed prior to PPM implantation affect the significant enhancement of LASr due to lower baseline of LASr.

## 97 EPICARDIAL‐TO‐ENDOCARDIAL ACTIVATION GRADIENTS AND CONDUCTION BLOCK DURING ATRIAL FIBRILLATION IN THE HUMAN POSTERIOR LEFT ATRIUM

### 97.1 **CHRISTOPHER WONG** ^1^, NITISH BADHWAR^2^, ANSON LEE^2^, RAMIN BEYGUI^3^, RANDALL LEE^3^

#### 97.1.1 ^1^University of Adelaide and Royal Adelaide Hospital, Adelaide, Australia,^2^Stanford University, Palo Alto, CA,^3^University of California, San Francisco, San Francisco, CA

**Introduction:** While emerging evidence supports 3D myocardial activation during atrial fibrillation (AF), human studies remain limited. We thus undertook electroanatomic and electrophysiologic characterization of the endocardial and epicardial posterior left atrium (PLA) during AF in humans.

**Methods:** Patients with symptomatic nonparoxysmal AF who had unsuccessful antiarrhythmic or catheter ablation therapy referred for hybrid epicardial‐endocardial AF ablation and left atrial appendage ligation underwent high‐density mapping of the PLA with grid catheters.

**Results:** Twenty patients (14 men, median 67 years) were included. All patients in sinus rhythm had similar endocardial and epicardial PLA bipolar voltage maps with synchronous, one‐to‐one endocardial‐epicardial activity. A majority (90%) of patients in AF demonstrated greater epicardial compared to endocardial PLA bipolar voltage during AF. Asynchronous endocardial‐epicardial PLA AF activation during simultaneous endocardial‐epicardial mapping was universal. Episodes of more rapid epicardial compared to endocardial PLA AF activation were observed in all simultaneous endocardial‐epicardial recordings, while only a third of patients had episodes of more rapid endocardial compared to epicardial PLA AF activation. Conduction block between the endocardial and epicardial surfaces was common during organized AF, with instances of isolated or multiple blocked beats, Wenckebach conduction, and sustained endocardial PLA entrance block with ongoing epicardial AF observed. Epicardial‐to‐endocardial block was also observed during sinus rhythm.

**Conclusions:** Endocardial‐epicardial PLA asynchrony is universal during persistent AF characterized by: 1) greater epicardial compared to endocardial PLA bipolar voltages during AF, 2) more frequent epicardial‐to‐endocardial PLA activation gradients during AF and 3) conduction block between the epicardial and endocardial surfaces during AF and sinus rhythm. These data strongly support the potential importance of the epicardial PLA with implications for ablation therapies.

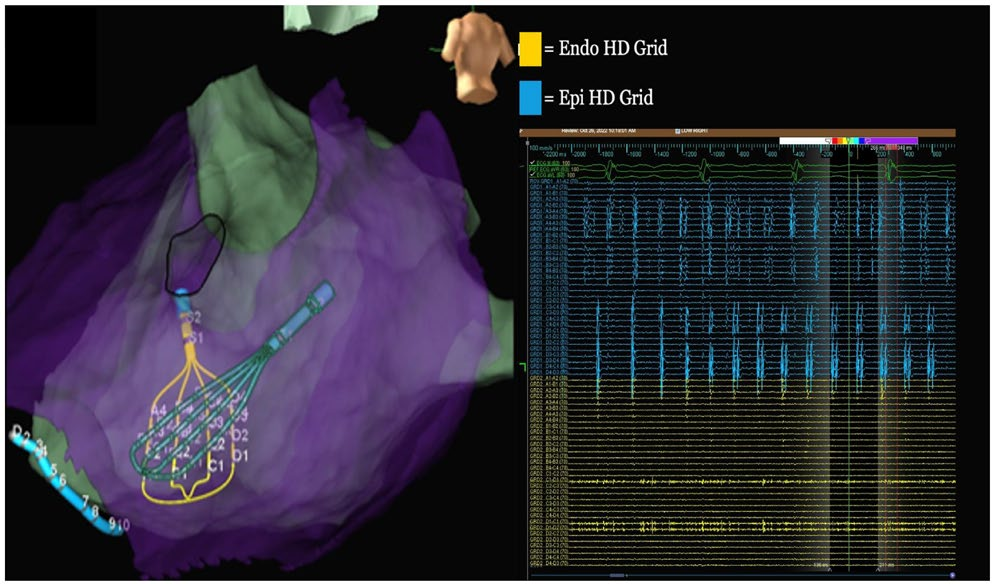

## 98 INSIGHTS INTO MITRAL ISTHMUS ABLATION IN THE TREATMENT OF PERSISTENT ATRIAL FIBRILLATION WITH A STEPWISE APPROACH UTILIZING PULSED FIELD ABLATION AND RADIOFREQUENCY ABLATION

### 98.1 **KELVIN CHEOK KENG WONG**, PAUL LIM, WEE SIONG TEO

#### 98.1.1 Mount Elizabeth Hospital, Singapore, Singapore

**Introduction:** Mitral Isthmus (MI) ablation is an important part of the ablation strategy for persistent atrial fibrillation (AF). However, it is technically challenging using radiofrequency (RF) ablation. The use of pulsed field ablation (PFA) has the potential to improve success.

**Methods:** In this case series, we report our experience in 16 patients undergoing MI ablation as part of the ablation strategy for AF. MI ablation was performed after pulmonary vein and posterior walI isolation using PFA (Farapulse; Boston Scientific) guided by 3‐D electroanatomical mapping. After administering IV GTN, MI ablation was performed using a stepwise approach: 1. PFA delivered in the flower configuration to the MI area at the 4‐5 o'clock position of the mitral annulus. 2. Endocardial mapping of the MI area during left atrial appendage pacing 3. Repeat pulsed PFA targetting areas with residual electrograms (emphasis on achieving good contact) 4. Endocardial ablation using RF (if necessary) 5. Epicardial ablation in distal coronary sinus (CS) using RF.

**Results:** MI block was achieved in all patients. The mean time for MI ablation was 23 mins. The mean number of PFA applications was 11 +/‐ 5. PFA alone achieved block in 3/16 patients (19%). Endocardial RF ablation was required for block in 6/16 patients (38%). Epicardial CS ablation was required for block in 7/16 patients (44%). Compared to historical data, a smaller percentage of patients required epicardial ablation to achieve MI block. A short learning curve was needed to achieve effective PFA in the MI area. In 2 patients, MI block was achieved with PFA, but recurrence of conduction eventually necessitated epicardial ablation. In 1 patient, there was epicardial disconnection but no endocardial block after PFA. There were no complications observed.

**Conclusions:** In this case series, it is feasible and safe to perform MI ablation utilizing both PFA and RF ablation. Further studies are needed to evaluate the efficacy of this stepwise approach.

## 99 EXAMINING THE EFFECTIVENESS AND RISKS OF LV TRIGGERED PACING: INSIGHTS FROM A CLINICAL CASE

### 99.1 **JULIET YOUNG**, ALEX DASHWOOD, HUI CHEN HAN

#### 99.1.1 Monash ‐ Victorian Heart Hospital, Clayton, Australia

**Introduction:** Interrogation of a cardiac resynchronisation therapy defibrillator (CRTD) revealed intermittent left ventricular (LV) fusion pacing during sustained ventricular tachycardia (VT), captured on an electrocardiogram (ECG).

**Methods:** N/A

**Results:** Triggered LV and biventricular (BiV) fusion pacing algorithms aim to increase CRT therapy delivery in patients with competing intrinsic conduction. However, the initial research models used to develop these algorithms often fail to replicate real‐world scenarios where fusion pacing typically occurs (e.g., resting conditions only, in patients who are all sinus rhythm with 1:1 atrioventricular (AV) conduction). With advancements in modern device algorithms that reduce the necessity for fusion pacing during AV conduction, in real‐life situations, triggered pacing often occurs during conducted atrial fibrillation or ventricular ectopy. Furthermore, recent investigations demonstrate no benefit of fusion pacing algorithms over pure intrinsic conduction. Additionally, case reports suggest that fusion pacing may be arrhythmogenic. We present a case study where triggered pacing exceeded programmed expectations, notably during a VT episode where rates reach up to 145 bpm, surpassing the programmed upper limit of 130 bpm. This rate violation can be attributed to Biotronik's triggered pacing algorithm, which exclusively factors in LV sensing into its timing cycle. In this specific scenario, LV undersensing occurs due to low amplitude resulting from infarcted myocardial tissue. Consequently, despite the ongoing VT, in the example shown the device is delivering LV triggered pacing at rates between 46‐70 bpm, which falls within the programmed maximum rate.

**Conclusions:** This case highlights inappropriate fusion pacing algorithm functioning, exceeding programmed expectations. Until research establishes clear benefits of fusion pacing algorithms in heart failure patients, cautious consideration or disabling of these algorithms is warranted.

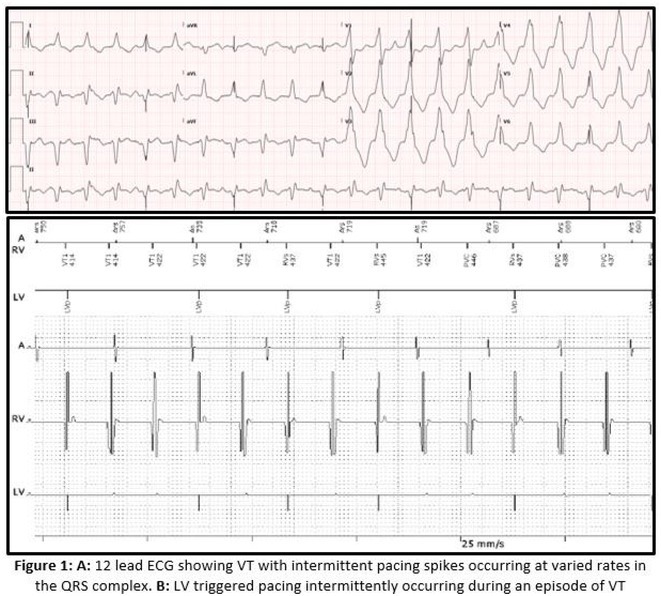

## 100 POST‐PROCEDURE ANTIBIOTIC PROPHYLAXIS FOR CARDIAC ELECTRICAL DEVICE IMPLANTATION (ABXFREE)

### 100.1 **CHIH‐CHIEH YU** ^1^, KUAN‐HUNG YEH^2^, HUNG‐YU CHANG^3^, YING‐CHIEH LIAO^4^, LIAN‐YU LIN^1^, LI‐TING HO^1^, JU‐YI CHEN^5^

#### 100.1.1 ^1^National Taiwan University Hospital, Taipei, Taiwan,^2^Taipei Tzu Chi Hospital, Taipei, Taiwan,^3^Cheng‐Hsin General Hospital, Taipei, Taiwan,^4^Changhuan Christian Hospital, Changhuan, Taiwan,^5^National Cheng Kung University, Tainan, Taiwan

**Introduction:** Post‐procedural prophylactic antibiotic (PPPA) administration is a widespread practice in Asia Pacific countries, particularly in the implantation or replacement of the cardiac implantable electric device (CIED). Despite its common use, previous retrospective studies have suggested a lack of efficacy associated with PPPA. Therefore, we conducted a multicenter, prospective, randomized clinical trial to further elucidate the efficacy of PPPA in this setting.

**Methods:** Patients who underwent CIED implantation or generator replacement were consecutively enrolled. All participants received pre‐procedural, single‐dose, parenteral antibiotic prophylaxis, and were subsequently randomized into two groups: group A received no PPPA, while group B received 3 days of post‐procedural oral antibiotics. Patients with end‐stage renal disease requiring regular dialysis or those undergoing cardiac resynchronization therapy were excluded. The primary outcome measure was hospitalization due to CIED‐related infection.

**Results:** This study involved five hospitals across northern, middle, and southern Taiwan. Between August 2019 and December 2022, a total of 294 patients were consecutively enrolled, comprising 162 (55.1%) new implant procedures and 132 (44.9%) generator replacements. Permanent pacemaker implantation accounted for the majority of procedures (256, 86.8%). CIED infection occurred in 1 (0.6%) patient in group A and 3 (2.2%) patients in group B. However, there was no statistically significant difference between the two groups (p= 0.295). Subgroup analysis identified dual‐chamber implantable cardioverter‐defibrillator (p=0.011) and pneumothorax occurrence (p=0.004) as potential risk factors for infection.

**Conclusions:** This study revealed a notably low overall infection rate, and there was no discernible benefit of PPPA in preventing CIED related infections. These results are consistent with previous studies and contribute further substantiation to this matter.

Chair

**Y. Yuniadi**;

National Cardiovascular Center Harapan Kita, Indonesia

Chair

**E. Zado**;

PA

Chair

**J. C. Zerpa**;

HCor ‐ Hospital do Coracao, Sao Paulo, Brazil

